# Shaping food system governance: mapping the national food and nutrition legislation landscape across Europe

**DOI:** 10.3389/fpubh.2026.1850537

**Published:** 2026-06-05

**Authors:** Nicoló Scarsi, Abdelrahman Taha, Doriana Lacalaprice, Giorgia Perazzoni, Luisa Soru, Salvatore Di Grande, Daniele D'Anna, Andrea Staffieri, Marius Geantǎ, Roberta Pastorino, Leonardo Villani, Stefania Boccia

**Affiliations:** 1 Section of Hygiene, Department of Life Sciences and Public Health, Università Cattolica del Sacro Cuore, Rome, Italy; 2 Department of Medicine and Surgery, University of Perugia, Perugia, Italy; 3 Center for Innovation in Medicine, Bucharest, Romania; 4 Department of Woman and Child Health and Public Health, Fondazione Policlinico Universitario A. Gemelli IRCCS, Rome, Italy; 5 UniCamillus—Saint Camillus International University of Health and Medical Sciences, Rome, Italy

**Keywords:** food environments, food systems, nourishing, nutrition policy, policy mapping, public health nutrition, scoping review

## Abstract

**Introduction:**

National nutrition policies play a pivotal role in promoting healthier dietary choices and reducing the consumption of unhealthy foods, thereby contributing to improved public health outcomes. Understanding their scope and implementation is essential for guiding regional health policies and strategies to improve food systems.

**Methods:**

We conducted a scoping review to map national-level nutrition policies, legislation, and regulatory instruments related to food production, labeling, marketing, pricing, and consumption across 31 European countries. We systematically searched PubMed, Scopus, FAOLEX, Google, and the Global Database on the Implementation of Food and Nutrition Action for records published between 2014 and 2024. Policy actions were categorized according to the NOURISHING framework.

**Results:**

Out of 6,299 screened records, we identified 379 national food and nutrition policy actions. Finland, France, Spain, and Sweden showed the broadest domain coverage, with policy actions identified in nine or more of the 10 NOURISHING policy areas, whereas Cyprus, Poland, and Latvia showed limited coverage, with five or fewer areas represented. The most regulated domain was food supply chain governance (32%), followed by nutritional quality of the whole food supply (19%) and labeling (15%). In contrast, the integration of nutrition services in healthcare settings (1%), and the use of economic incentives (1%), were underrepresented.

**Conclusion:**

European countries showed marked variation in the adoption of nutrition policies. While progress has been made in food safety and information provision, significant gaps remain in behavior-shaping policies. These disparities may reinforce existing health inequalities. Coordinated EU-level strategies, stronger enforcement mechanisms, and broader uptake of evidence-based interventions are needed to support a more equitable and effective transformation of food systems across the region.

## Introduction

Dietary habits are a key determinant of health and lie at the core of major public health challenges worldwide. Proper nutrition can prevent the onset of many non-communicable diseases (NCDs) such as obesity, cardiovascular diseases, diabetes, and cancer (1, 2). Conversely, unhealthy diets are a major risk factor for several chronic conditions, contributing to 7.9 million deaths and 187.7 million disability-adjusted life years (DALYs) globally, with the highest burden occurring in densely populated regions (3). The leading global causes of mortality and DALYs from diet-related NCDs include ischemic heart disease, stroke, diabetes mellitus, colorectal cancer, hypertensive heart disease, chronic kidney disease, and cancers of the lung, esophagus, stomach, and breast (3).

Various social, economic, cultural, educational, and environmental factors can influence individuals' food choices (4–6). These include household income and food affordability, availability and accessibility of healthy foods, cultural norms and dietary traditions, nutrition knowledge and health literacy, exposure to food marketing, school and workplace food environments, and broader commercial and regulatory conditions. Given the significant health and economic burden associated with poor dietary habits, governments play a crucial role in shaping consumer behaviors through health and social policies (7–10). This is particularly relevant for children and adolescents, whose dietary habits and food preferences are still developing and may shape dietary trajectories and long-term health outcomes across the life course (11, 12). Policy measures such as sugar taxes, front-of-pack nutritional labeling, and regulations on school meal programmes have shown promising results in improving dietary choices and reducing obesity rates (7, 8, 13–15). Targeted government policies and legislation can help reverse obesogenic food environments, reduce health inequalities, and mitigate the economic burden associated with healthcare costs and productivity losses (7–10).

In Europe, dietary patterns and food-related health outcomes vary significantly across countries, reflecting differences in national policies, food availability, and socioeconomic conditions (16, 17). The implementation of nutritional policies, however, has been inconsistent across European countries, primarily due to variations in enforcement mechanisms and population-level acceptance (18–20).

Despite growing recognition of the role of food and nutrition policies in preventing diet-related non-communicable diseases, comparative evidence on national policy actions across Europe remains fragmented. Existing studies often focus on specific policy areas or selected countries, limiting the ability to compare national policy portfolios and identify gaps across food environments and food systems. A structured mapping of national policy actions using the NOURISHING framework (21), developed by the World Cancer Research Fund International (WCRFI), can help clarify where policy action is concentrated, which domains remain underused, and where coordinated European and national efforts may be needed to strengthen public health nutrition governance. This scoping review aimed to map national-level food and nutrition policy actions addressing food production, sale, labeling, marketing, pricing, and consumption across selected European countries, and to classify these actions using the NOURISHING framework.

## Methods

This scoping review was conducted in accordance with the methodological framework proposed by Arksey and O'Malley (22) and is reported following the Preferred Reporting Items for Systematic Reviews and Meta-Analyses Extension for Scoping Reviews (PRISMA-ScR) guidelines (23). The completed PRISMA-ScR checklist is provided in Supplementary Table S1. This scoping review was guided by a research question aimed at systematically identifying national-level food and nutrition policy actions addressing food production, sale, labeling, marketing, pricing, and consumption across selected European countries, and examining their distribution across the domains of the NOURISHING framework.

The review was conducted as part of the 4P-CAN project (Personalized Cancer Primary Prevention Research through Citizen Participation and Digitally-Enabled Social Innovation), which aims to strengthen cancer primary prevention and reduce inequalities in policy implementation, particularly in Eastern European countries. The research protocol was made publicly available on the Open Science Framework (OSF) platform (doi: 10.17605/OSF.IO/8DXP6) (24).

### Search strategy and definitions

We conducted a comprehensive search across both scientific databases (PubMed and Scopus) and gray literature sources, including FAOLEX (Food, Agriculture, and Natural Resources Management Legislation Database) (25), the Global Database on the Implementation of Food and Nutrition Action (GIFNA) (26), and Google. To identify policy actions targeting diet-related risk factors and promoting healthy dietary habits at the population level, we developed a search query combining keywords related to the main concepts of our research question. The complete search strategy is provided in Supplementary Table S2. In PubMed and Scopus, the search strategy focused on review-type publications because these sources were expected to synthesize existing national policy actions and provide efficient entry points for identifying relevant instruments across multiple countries. Scientific reviews were therefore used primarily as source records for policy identification and reference tracking, rather than as the sole basis for policy inclusion. Policy actions identified through peer-reviewed reviews were subsequently checked against gray literature databases and, where available, official legislative, governmental, FAOLEX, or GIFNA records to confirm their content, status, and most recent version.

Gray literature searches were conducted in FAOLEX, GIFNA, and Google to identify national-level policy actions not captured in scientific databases. In FAOLEX, searches were conducted by country and by relevant food, nutrition, health, agriculture, consumer protection, and food safety categories, with records screened for legislation, regulations, decrees, ordinances, strategies, action plans, and other national instruments relevant to food and nutrition policy. In GIFNA, records were browsed by country and policy area, with attention to national actions addressing food composition, labeling, marketing, public institutions, fiscal measures, nutrition education, healthcare-based nutrition advice, and food system governance. Google searches combined the general food and nutrition policy terms listed in Supplementary Table S2 with each included country name and, where relevant, additional terms such as “national strategy,” “law,” “regulation,” “decree,” “ordinance,” “nutrition action plan,” “food safety,” “food labeling,” “school meals,” and “public health nutrition.” Search results were screened until no additional eligible national-level records were identified. Only records meeting the eligibility criteria and describing formally enacted national-level policy actions in force at the time of data extraction were retained.

For this review, we used the definition of policy actions presented by the World Cancer Research Fund International (WCRFI) (21, 27), defined as those specific instruments put into place by any level of government to achieve a health objective, such as legislation, regulations, decrees, policies, programs, fiscal measures, government-supported voluntary actions, and campaigns. Although this definition can include instruments implemented at any level of government, the present review was restricted to national-level policy actions to allow cross-country comparison across the included European countries.

We piloted the search strategy to ensure the retrieval of relevant records. The search period covered records published, indexed, or made available between January 2014 and January 2024. This time window was selected to capture the most recent decade of scientific and policy documentation while allowing the identification of older national policy actions that remained in force during the review period. Therefore, the 2014–2024 restriction applied to the publication, indexing, or availability date of the sources used to identify policy actions, not to the year in which the policy actions entered into force. Older policy actions were included when they were identified in eligible sources published or updated within the search period and were formally enacted and in force at the time of data extraction.

### Eligibility criteria and assessment

#### Inclusion criteria

We included peer-reviewed reviews and official reports describing national-level policy actions related to food and nutrition, regardless of language. Peer-reviewed reviews were used to identify relevant policy actions and source trails, while legal databases, government sources, and gray literature records were used to verify and complement policy-level information where available. Only policy actions that were formally enacted and in force at the time of data extraction were considered. The in-force status of policy actions was assessed using the most recent and authoritative source available for each policy action. Where possible, official legal texts, government websites, FAOLEX records, GIFNA records, or other official database entries were prioritized over secondary sources. When a policy action was initially identified through a peer-reviewed review, report, or other secondary source, reviewers used that source as an entry point and, where available, checked the policy against official or database sources to confirm its enactment status, content, and most recent version. Policy actions described only as proposed, draft, planned, expired, repealed, or no longer active were excluded. For amended or revised instruments, the most recent version identified as being in force at the time of data extraction was retained. In cases where policies or legislative instruments had undergone amendments or revisions, the most recent version in force was included. The review covered all the 27 EU countries and 4 non-EU countries participating in the 4P-CAN consortium (Montenegro, North Macedonia, Republic of Moldova, and Ukraine).

#### Exclusion criteria

We excluded records if they: (i) referred only to subnational contexts (e.g., regional, provincial, or municipal level); (ii) concerned non-enacted measures (e.g., drafts); (iii) consisted of standards, procedures, decisions, recommendations, or opinions; or (iv) focused solely on alcoholic or sugar-sweetened beverages. Records focused solely on sugar-sweetened beverages were excluded because this policy area has been extensively mapped and evaluated in recent dedicated reviews, particularly in relation to beverage taxation and health outcomes. The present review aimed to characterize broader national food and nutrition policy actions across the food system and food environment, including labeling, marketing, reformulation, public institutions, healthcare-based nutrition advice, education, retail environments, and cross-sectoral governance. Broader policies that included sugar-sweetened beverages as part of a wider food or nutrition policy action were not excluded.

#### Screening of records

We managed the records identified through the search using Rayyan software (28), Google Drive, and Google Sheets. After removing duplicates, eight authors screened titles, abstracts, and full texts according to the eligibility criteria. Two reviewers independently screened all records from academic databases. Seven reviewers screened gray literature records according to the same eligibility criteria, and a separate researcher cross-checked the screening decisions to ensure consistency. Disagreements or uncertain eligibility decisions were resolved through discussion among the reviewers. When consensus could not be reached, a senior reviewer made the final inclusion decision.

### Data extraction and classification

Each included national policy action was assigned a unique identification code, combining the country abbreviation with a sequential number, for example ES_2 for the second policy action identified for Spain. These policy IDs were used throughout the Results to link illustrative country examples to the full policy-level information reported in Supplementary Table S3. When multiple records or sources referred to the same policy action, these were used for verification and source triangulation but did not generate duplicate policy entries. In such cases, the policy action was counted once, and the most complete, official, or up-to-date source available was retained as the primary reference. From each included document or record, we extracted the following information: (i) Source of reference; (ii) Type of source document (e.g., peer-reviewed article, government report, legal text, or database record); (iii) Legal nature of the policy action (e.g. Law, policy, ordinance, and decree); (iv) Year of entry into force; (v) Country; (vi) General description; (vii) Primary aim; (viii) Main institution responsible for promulgation; and (ix) Target population. For each included policy action, the source used for identification or verification was recorded in Supplementary Table S3. Where available, official legal texts, government sources, FAOLEX records, or GIFNA records were prioritized. When policy actions were identified through peer-reviewed reviews or reports, these secondary sources were used as entry points and were checked against official or database sources where possible.

Based on their legal nature, policy actions were classified into three categories: policies, laws, and other legislative instruments. *Policies* were defined as sets of decisions or commitments adopted by public authorities to establish objectives and guiding principles for addressing specific public issues, typically articulated through national strategies, action plans, or policy frameworks and not necessarily legally binding. *Laws* were defined as legally binding rules of conduct enacted by a legislative body (e.g., a parliament) and enforced by public institutions, creating legal obligations for individuals, organizations, and public authorities, with potential sanctions in cases of non-compliance. *Other legislative instruments* included legally binding or quasi-binding acts that do not constitute primary legislation, such as regulations, decrees, ordinances, ministerial directives, or administrative rules adopted under the authority of primary legislation.

The NOURISHING framework was selected because it provides a comprehensive and internationally recognized structure for categorizing government actions that shape food environments, food systems, and nutrition-related behaviors. Its domains cover regulatory, fiscal, informational, educational, health-service, and food supply interventions, making it suitable for mapping the breadth of national food and nutrition policy actions across countries. The framework was also appropriate for this review because it allows heterogeneous policy instruments, including laws, regulations, strategies, programmes, fiscal measures, and voluntary government-supported actions, to be classified within a common analytical structure. The extracted national policy actions were then classified using the *NOURISHING* framework. Each letter in “NOURISHING” corresponds to a specific policy action area: *N* (Nutrition label standards and regulations on the use of claims and implied claims on food), *O* (Offer healthy food and set standards in public institutions and other specific settings), *U* (Use economic tools to address food affordability and purchase incentives), *R* (Restrict food advertising and other forms of commercial promotion), *I* (Improve the nutritional quality of the whole food supply), *S* (Set incentives and rules to create a healthy retail and food service environment), *H* (Harness supply chain and actions across sectors to ensure coherence with health), *I* (Inform people about food and nutrition through public awareness), *N* (Nutrition advice and counseling in healthcare settings), and *G* (Give nutrition education and skills). Classification into NOURISHING domains was performed independently by two trained reviewers using the official framework definitions and predefined domain descriptions. Each policy action was reviewed for its stated aim, target population, targeted products or food components, and main mechanism of action before being assigned to one or more NOURISHING domains. Because some policy actions addressed more than one area of food and nutrition governance, multiple domain classifications were permitted when justified by the policy content. Discrepancies between reviewers were resolved through discussion, and unresolved cases were adjudicated by a senior reviewer.

To support cross-country comparison, we calculated a NOURISHING domain coverage score for each country, defined as the number of NOURISHING policy areas in which at least one national policy action was identified. The score ranged from 0 to 10. Countries were described as showing broad multidomain engagement when policy actions were identified in nine or more NOURISHING policy areas. Countries were described as showing limited coverage when policy actions were identified in five or fewer NOURISHING policy areas. Countries with policy actions in six to eight domains were interpreted as having intermediate coverage and were not highlighted as either broadest or most limited.

## Results

**Figure 1:**
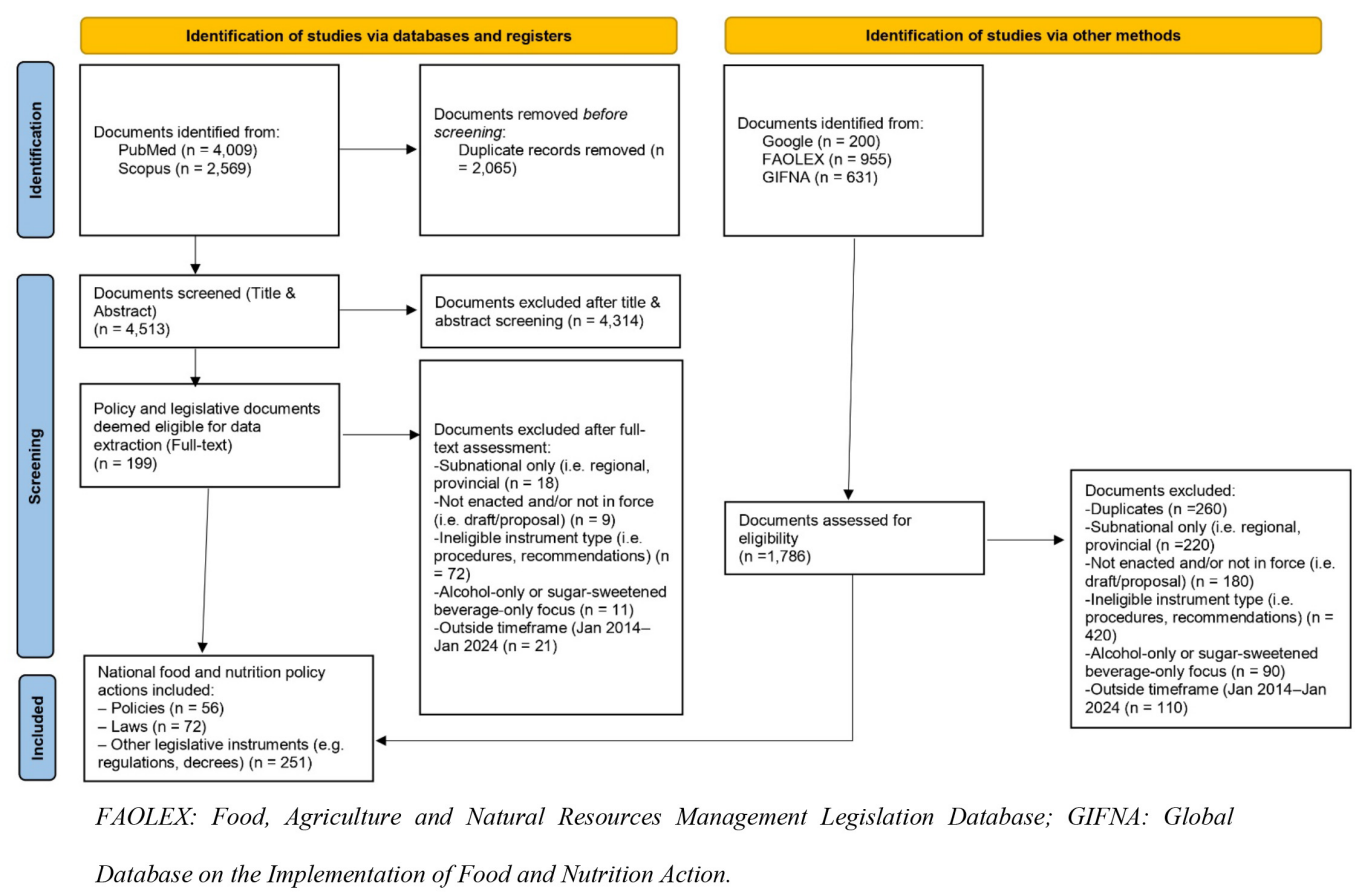
PRISMA flowchart of the identification, screening, and inclusion of national food and nutrition policy and legislative documents. FAOLEX, food, agriculture and natural resources management legislation database; GIFNA, global database on the implementation of food and nutrition action.

After duplicates removal, we retrieved 4,513 records from scientific databases, of which 68 articles met the inclusion criteria and were used as source records for identifying relevant policy actions. In addition, 409 records were identified from gray literature sources, including FAOLEX (*n* = 254; 62%), GIFNA (*n* = 138; 34%), and Google (*n* = 17; 4%). These source records did not correspond one-to-one with policy actions: a single source could describe more than one policy action, and multiple sources could refer to the same policy action. After screening, deduplication, and verification at the policy-action level, a total of 379 unique national food and nutrition policy actions were included and categorized (Figure 1). The included policy actions were unevenly distributed across countries (Supplementary Table S4).

The number of policy actions identified for each country should be interpreted as a measure of policy volume rather than policy breadth. A higher number of policy actions did not necessarily indicate more comprehensive multidomain engagement, because several actions could be concentrated within the same NOURISHING policy area. This was particularly evident for the Republic of Moldova, which accounted for the largest number of included policy actions but was not classified among the countries with the broadest multidomain coverage. Many of the Moldovan policy actions identified were administrative or regulatory instruments related to food additives, dietary supplements, food safety, and supply-chain governance, resulting in a high count of policy actions but a narrower distribution across NOURISHING domains. The Republic of Moldova accounted for the largest share (16.4%; *n* = 62), followed by Germany (6.9%; *n* = 26), Bulgaria (5.5%; *n* = 21), Croatia (5.0%; *n* = 19), and Montenegro (5.0%; *n* = 19). Most other countries contributed between 1 and 4% of the total policy actions, while Cyprus (0.8%; *n* = 3), Austria (1.1%; *n* = 4), and Slovakia (1.1%; *n* = 4) showed the lowest representation.

Based on legal nature, the majority of included policy actions were classified as other legislative instruments, accounting for 60.3% (*n* = 229) of all policy actions (*N* = 379). These primarily included decrees (*n* = 87), regulations (*n* = 73), ministerial orders (*n* = 44), and ordinances (*n* = 24). Laws represented 19.8% (*n* = 75) of policy actions, while policies, including national strategies, action plans, and programmes, also accounted for 19.8% (*n* = 75). The temporal distribution showed a concentration in recent years: 10.8% (*n* = 41) entered into force before 2000 and 21.6% (*n* = 82) between 2000 and 2009, whereas 67.0% were implemented from 2010 onwards, with 39.8% (*n* = 151) entering into force between 2020 and 2024. These earlier policy actions were retained because the search-period restriction applied to the date of the source record rather than to the policy entry-into-force date, and because these actions were identified as still in force at the time of data extraction. With regard to target population, most policy actions addressed the general population, while 18.0% (*n* = 68) explicitly targeted children and adolescents, primarily through school food provision and standards, restrictions on marketing to minors, limits on unhealthy foods in school settings, and nutrition education initiatives.

Regarding targeted products, policy actions most frequently applied to the overall food supply, with nearly half targeting all food products (47.9%, *n* = 187). A smaller set of policy actions targeted institutional settings and specific product groups, most commonly school food products (7.4%, *n* = 29), followed by dietary supplements (5.6%, *n* = 22) and infant food products (3.8%, *n* = 15). The remaining policy actions targeted a wide range of specific products, including animal-origin products, bread/bakery products, poultry meat, organic products, plant protection products, and food-contact materials (31.8%, *n* = 126). Regarding targeted components, almost half of policy actions did not specify a component (49.5%, *n* = 193). Among policy actions that did, targets most commonly related to nutrient composition and ingredient controls, led by nutrients (generic; 11.5%, *n* = 45), salt/sodium (10.0%, *n* = 39), and food additives (8.7%, *n* = 34). The remaining policy actions addressed a broad set of additional components (16.9%, *n* = 68), including trans fats, pesticides/residues, and sugars, as well as less frequent targets such as extraction solvents, contaminants, and nitrates. The full list of included policy actions, extracted characteristics, and corresponding sources or references is provided in Supplementary Table S3.

### Categorization of nutrition policy actions against the NOURISHING framework

The distribution across domains is detailed in Table 1. Overall, policy activity was unevenly distributed across domains, with the strongest concentration in actions across sectors to ensure coherence with health, particularly policy actions addressing food supply chains, food safety, contamination control, and regulatory coherence. In contrast, domains related to the use of economic tools (1.2%; *n* = 10) and the integration of nutrition counseling within healthcare services (1.0%; *n* = 8) were comparatively underrepresented.

**Table 1:** Distribution of national policy action classifications across the NOURISHING framework domains.

NOURISHING macro categories		NOURISHING policy areas	*N*	%
**Food environment**	**N**	*Nutrition label standards and regulations*	118	15%
	**O**	*Offer healthy food in public institutions and other settings*	64	8%
	**U**	*Use economic tools to address food affordability and purchase incentives*	10	1.2%
	**R**	*Restrict food advertising and other forms of promotion*	25	3%
	**I**	*Improve nutritional quality of the whole food supply*	153	19%
	**S**	*Set incentives and rules to create a healthy retail environment*	95	12%
**Food system**	**H**	*Harness food supply chains to create healthier diets*	257	32%
**Behavior change communication**	**I**	*Inform people about food and nutrition through public awareness*	50	6%
	**N**	*Nutrition advice and counseling in health care settings*	8	1%
	**G**	*Give nutritional education and skills*	24	3%
**Total**			**804**	**100%**

N and percentages refer to NOURISHING domain classifications rather than unique policy actions. Because individual national policy actions could be assigned to more than one NOURISHING domain, the total number of domain classifications (N = 804) exceeds the number of unique national policy actions included in the review (N = 379). Percentages were calculated using the total number of domain classifications as the denominator.

An overview of the national policy actions included in the NOURISHING framework by country is reported in Table 2. Based on the NOURISHING domain coverage score, Finland, France, Spain, and Sweden showed broad multidomain engagement, with policy actions identified in nine or more of the ten NOURISHING policy areas. In contrast, Cyprus, Poland, and Latvia showed limited coverage, with policy actions identified in five or fewer policy areas. Countries with policy actions in six to eight domains were interpreted as having intermediate coverage. Together, these patterns highlight substantial cross-country variability in both the scope and strategic orientation of national food and nutrition policies. Policy identifiers shown in square brackets throughout the Results refer to the unique country-specific policy IDs listed in Supplementary Table S3.

**Table 2:** Policy actions included according to the NOURISHING framework, by country.

NOURISHING framework macro categories	NOURISHING framework policy area		AT	BE	BG	HR	CY	CZ	DK	EE	FI	FR	DE	GR	HU	IE	IT	LV	LT	LU	MT	NL	PL	PT	RO	SK	Sl	ES	SE	MD	UA	MK	ME
FOOD ENVIRONMENT	N	Nutrition label standards and regulations on the use of claims and implied claims on food	✓	✓	✓	✓	✓	✓	✓	✓	✓	✓	✓		✓	✓	✓	✓	✓	✓	✓	✓	✓	✓	✓	✓		✓	✓	✓	✓	✓	✓
	O	Offer healthy food and set standards in public institutions and other specific settings	✓		✓	✓		✓		✓	✓	✓	✓	✓	✓	✓		✓	✓	✓	✓			✓	✓	✓	✓	✓	✓	✓	✓		✓
	U	Use economic tools to address food affordability and purchase incentives							✓		✓		✓		✓		✓							✓					✓				✓
	R	Restrict food advertising and other forms of commercial promotion	✓	✓	✓			✓	✓		✓	✓				✓			✓	✓	✓	✓		✓	✓		✓	✓	✓	✓		✓	
	I	Improve nutritional quality of the whole food supply	✓	✓	✓	✓		✓	✓	✓	✓	✓	✓	✓	✓	✓	✓	✓	✓	✓	✓	✓	✓	✓	✓	✓	✓	✓	✓	✓	✓	✓	✓
	S	Set incentives and rules to create a healthy retail and food service environment	✓	✓	✓	✓		✓	✓	✓	✓	✓	✓	✓	✓		✓	✓	✓	✓	✓	✓		✓	✓	✓	✓	✓	✓	✓	✓		✓
FOOD SYSTEM	H	Harness supply chain and actions across sectors to ensure coherence with health	✓	✓	✓	✓	✓	✓	✓	✓	✓	✓	✓	✓	✓	✓	✓	✓	✓	✓	✓	✓	✓	✓	✓	✓	✓	✓	✓	✓	✓	✓	✓
BEHAVIOR CHANGE COMMUNICATION	I	Inform people about food and nutrition through public awareness	✓	✓	✓	✓		✓	✓	✓	✓	✓		✓	✓		✓		✓	✓		✓					✓	✓	✓	✓	✓	✓	
	N	Nutrition advice and counseling in healthcare settings	✓	✓								✓	✓			✓							✓					✓		✓			
	G	Give nutrition education and skills		✓				✓	✓		✓	✓	✓	✓	✓	✓	✓			✓	✓					✓	✓	✓	✓			✓	

European Union (EU) countries (ISO Alpha-2 Code): AT: Austria; BE: Belgium; BG: Bulgaria; HR: Croatia; CY: Cyprus; CZ: Czech Republic; DK: Denmark; EE: Estonia; FI: Finland; FR: France; DE: Germany; GR: Greece; HU: Hungary; IE: Ireland; IT: Italy; LV: Latvia; LT: Lithuania; LU: Luxembourg; MT: Malta; NL: Netherlands; PL: Poland; PT: Portugal; RO: Romania; SK: Slovakia; Sl: Slovenia; ES: Spain; SE: Sweden. Extra EU countries: MD: Republic of Moldova; UA: Ukraine; MK: North Macedonia; ME: Montenegro.

Country-level coverage was assessed using a NOURISHING domain coverage score, defined as the number of NOURISHING policy areas in which at least one national policy action was identified. Broad multidomain engagement was defined as coverage of nine or more policy areas, limited coverage as five or fewer policy areas, and intermediate coverage as six to eight policy areas.

Country-level domain coverage should be interpreted alongside, but separately from, the number of policy actions identified per country, as multiple policy actions may fall within the same NOURISHING policy area.

### Nutrition label standards and regulations on the use of claims and implied claims on food (N)

Policies within the nutrition labeling and claims domain primarily focused on standardizing nutrient claims, warning labels, and consumer information, with front-of-pack labeling widely adopted to support healthier food choices. Overall, national approaches emphasized transparency and harmonization of food information rather than product-specific regulation. Spain exemplifies a comprehensive regulatory approach, having established broad rules to standardize food labeling in support of consumer health and informed decision-making (Supplementary Table S3: ES_2). In contrast, several Nordic countries have implemented the Keyhole front-of-pack labeling scheme, an internationally recognized system designed to guide consumers toward healthier food options across multiple product categories [SE_1, DK_6, LT_3]. Other countries adopted front-of-pack labeling approaches based on the Nutri-Score scheme, including Belgium, France, and Luxembourg, illustrating the wider use of interpretive labeling systems to support consumer understanding of nutritional quality [BE_10, FR_15, LU_3].

### Offer healthy food and set standards in public institutions and other specific settings (O)

Policies within this domain predominantly focused on shaping food environments in public institutions, particularly schools, with the aim of promoting healthier dietary behaviors and preventing childhood obesity and diet-related non-communicable diseases. Most measures targeted children by establishing mandatory nutritional standards for meals provided in educational and childcare settings, as well as by restricting the availability of unhealthy foods. France represents a comprehensive approach, having introduced binding national standards for school meals that define nutritional requirements related to meal composition, portion sizes, and food provision in public canteens [FR_12]. Similarly, several countries adopted regulatory frameworks to improve the nutritional quality of institutional catering, often complemented by broader initiatives supporting healthier food environments, such as school-based nutrition programs and restrictions on the marketing of unhealthy foods to children.

### Use economic tools to address food affordability and purchase incentives (U)

Policies addressing food affordability and purchase incentives were relatively rare across countries, accounting for 1.2% of NOURISHING domain classifications (*n* = 10), indicating limited use of economic instruments to influence dietary behaviors. Where implemented, measures primarily relied on fiscal tools to discourage the consumption of unhealthy foods, particularly through taxes on products high in sugar, salt, or fat. Hungary represents a notable example, having introduced a national public health product tax targeting energy-dense foods and beverages with unfavorable nutritional profiles [HU_7]. Similarly, Portugal applied differential value-added tax rates, imposing higher taxes on sugary and salty processed foods while maintaining reduced rates for non-processed items to encourage healthier choices [PT_2]. Overall, the limited adoption of such measures highlights the underutilization of economic levers in national nutrition policy frameworks.

### Restrict food advertising and other forms of commercial promotion (R)

Policies restricting food advertising and commercial promotion primarily focused on protecting children from exposure to the marketing of unhealthy foods, particularly across television and digital media. Where present, national approaches aimed to limit the promotion of products high in sugar, salt, and fat through a combination of advertising bans, content restrictions, and responsible marketing standards. Portugal represents a binding regulatory approach, having enacted legislation prohibiting the advertising of unhealthy foods during children's television programs, near schools, and on child-oriented digital platforms [PT_7]. In contrast, Spain reinforced responsible food and beverage marketing to children through the PAOS Code, a self-regulatory framework requiring accuracy, age-appropriate messaging, and restrictions on persuasive techniques in advertising directed at children under 12 years of age [ES_4]. Overall, countries relied on a mix of statutory measures and voluntary codes, with substantial variation in regulatory strength and scope.

### Improve nutritional quality of the whole food supply (I)

Policies aimed at improving the nutritional quality of the overall food supply predominantly focused on product reformulation to reduce the content of salt, sugar, and industrially produced trans-fatty acids. Across countries, reformulation strategies were most commonly applied to widely consume staple foods, particularly bread and bakery products, reflecting efforts to achieve population-level dietary improvements. France represents an early and sustained approach to salt reduction, having implemented measures promoting reformulation and the regulated composition of table salt to support public health objectives [FR_3]. In parallel, several countries adopted targeted compositional limits for harmful nutrients, such as Romania, which introduced binding legislation to cap industrial trans-fatty acids in food products, applying equally to domestically produced and imported goods [RO_7]. Overall, these policies illustrate a regulatory focus on modifying the nutritional profile of commonly consumed foods as a key strategy to improve diet quality.

### Set incentives and rules to create a healthy retail and food service environment (S)

Policies aimed at creating healthier retail and food service environments primarily focused on regulatory and incentive-based measures designed to improve the nutritional quality of foods available to consumers across retail and catering settings. National approaches commonly addressed food composition, product standards, and the availability of healthier options, seeking to facilitate healthier purchasing decisions along the food supply chain. Portugal exemplifies an integrated strategy in this domain, having implemented national programmes to reduce sugar, salt, and trans-fat content in processed foods, establish nutritional standards for selected food products, and increase access to healthier food choices [PT_4, PT_5]. Overall, measures in this domain combined product-focused regulation and reformulation-oriented strategies, reflecting heterogeneous but complementary approaches to shaping healthier food environments.

### Harness supply chain and actions across sectors to ensure coherence with health (H)

Policies within this domain primarily focused on aligning food production, distribution, and marketing with public health objectives through cross-sectoral regulatory frameworks. Across countries, the dominant emphasis was on food safety and contamination control, particularly through measures addressing hygiene standards, pesticide residues, and nitrate limits along the food supply chain. Spain exemplifies a comprehensive, system-wide approach, having implemented multiple regulatory measures covering food safety, labeling, composition, and hygiene across production and marketing stages, while ensuring alignment with European Union requirements [ES_6, ES_10]. Similarly, Bulgaria adopted an overarching Food Law regulating food safety across the entire supply chain, incorporating hazard control principles and enforcement mechanisms to ensure compliance from production to retail [BG_6]. Overall, policies in this domain reflect a strong regulatory orientation toward safeguarding food safety and ensuring coherence between agricultural, food, and health sectors.

### Inform people about food and nutrition through public awareness (I)

Policies aimed at informing people about food and nutrition primarily relied on public awareness campaigns, educational initiatives, and communication strategies delivered through schools, media, and national health programs. Across countries, these measures sought to promote dietary recommendations, improve understanding of nutrient content, and support healthier eating behaviors through population-level messaging rather than regulatory intervention. Spain provides a representative example, having implemented a national strategy combining public education, school-based interventions, and awareness campaigns to improve dietary habits and address obesity [ES_3]. Similarly, Belgium adopted policies targeting children and adolescents that integrated nutrition education, physical activity promotion, and media literacy to foster healthier lifestyles [BE_8]. Overall, this domain reflects a widespread reliance on informational and educational approaches to influence dietary behaviors.

### Nutrition advice and counseling in healthcare settings (N)

Policies integrating nutrition services into healthcare systems primarily focused on embedding nutritional counseling and preventive interventions within primary care and broader health service delivery. Across countries, these measures aimed to support obesity prevention, cardiovascular risk reduction, and the management of diet-related non-communicable diseases by strengthening the role of nutrition in clinical practice. Austria provides an illustrative example through its National Nutrition Action Plan, which promoted cross-sectoral collaboration and explicitly supported the integration of nutrition into healthcare delivery pathways [AT_4]. Similarly, Poland incorporated nutritional counseling within its National Health Program as part of a wider package of healthcare services targeting obesity prevention and healthy aging [PL_8]. Overall, policies in this domain reflect a relatively limited but emerging effort to institutionalize nutrition within health systems.

### Give nutrition education and skills (G)

Policies aimed at developing nutrition education and skills primarily focused on integrating nutrition and physical activity content into formal education systems, with particular emphasis on children and adolescents. Across countries, school-based interventions were the dominant approach, seeking to promote healthy lifelong habits through curricular activities, educational programs, and supportive school environments. Luxembourg and Malta provide illustrative examples, having embedded comprehensive health and nutrition education within school curricula to encourage balanced lifestyles from an early age [LU_2, MT_4, MT_5]. Similarly, Belgium adopted a whole-of-society approach, promoting nutrition education through coordinated actions involving schools, families, and local authorities [BE_8]. Overall, while education-based policies are present across several countries, their implementation remains uneven, reflecting variability in national prioritization of nutrition education within broader health strategies.

## Discussion

Our comprehensive analysis of national food and nutrition policy actions across 31 European countries reveals significant heterogeneity in both policy scope and implementation. Using the established NOURISHING framework (21), which categorizes policies into 10 evidence-based domains spanning food environments, food systems, and behavior change communication, this study provides a robust lens through which to examine the current legislative landscape.

The findings underscore the critical role of governmental policies as key instruments for health promotion and dietary risk reduction (7, 8, 10). Countries with broad multidomain policy engagement, defined as policy actions identified in nine or more NOURISHING policy areas, notably Finland, France, Spain, and Sweden, illustrate more diversified national policy portfolios addressing multiple determinants of diet and health. These approaches encompass food supply chain regulation, consumer information measures, restrictions on commercial promotion, and standards for public food provision, reflecting the systematic integration of health objectives across diverse regulatory arenas and strengthening both food environments and consumer empowerment.

Conversely, countries such as Cyprus, Poland, and Latvia showed limited policy coverage, defined as policy actions identified in five or fewer NOURISHING policy areas, particularly with respect to economic tools and the integration of nutrition counseling within healthcare services. Several structural, political, and socio-economic factors may help explain the observed cross-country variability in policy breadth. Differences in governance capacity, administrative organization, regulatory traditions, public health prioritization, and available financial or technical resources can influence whether countries adopt broad, integrated policy portfolios or focus on narrower sets of instruments. Countries with stronger institutional capacity and more established intersectoral coordination mechanisms may be better positioned to implement policy actions across multiple NOURISHING domains, including labeling, public procurement, reformulation, marketing restrictions, and health-service integration. Conversely, countries facing economic constraints, fragmented governance structures, or competing policy priorities may rely more heavily on narrower regulatory instruments or on actions concentrated in specific areas such as food safety, labeling, or supply-chain control. These contextual factors should be considered when interpreting cross-country comparisons, as the number of mapped policy actions alone does not necessarily reflect policy comprehensiveness, implementation quality, or population-level impact. These gaps may contribute to, and potentially exacerbate, existing health disparities, as disadvantaged populations remain disproportionately exposed to unhealthy food environments and marketing pressures. Notably, the observed North–South and East–West gradient in policy adoption mirrors broader socio-economic and health inequalities across Europe (20, 29, 30). This interpretation should be considered cautiously, as country-level policy coverage is shaped by multiple factors, including governance capacity, legal traditions, administrative structure, data availability, and national policy priorities.

The finding that the Republic of Moldova accounted for the highest number of identified policy actions, while not being classified among the countries with broad multidomain engagement, illustrates the importance of distinguishing between policy volume and policy breadth. A high number of policy actions may reflect a more fragmented legal or administrative structure; greater visibility of specific records in the databases searched, or repeated regulatory updates within a narrow policy area. In Moldova, many identified actions related to food additives, dietary supplements, food safety, and supply-chain governance, rather than being evenly distributed across the full range of NOURISHING domains. Therefore, country-level policy counts should not be interpreted as direct indicators of policy comprehensiveness or implementation strength. Domain coverage provides a complementary measure of the breadth of national policy portfolios, while policy counts describe the volume of mapped instruments.

While many countries have enacted policy actions addressing core areas such as food safety and food labeling, their long-term effectiveness may depend on legal form, enforcement design, and implementation capacity. In this review, only 19.8% of included policy actions were classified as laws, while most were classified as other legislative instruments or policies. This distribution suggests that a substantial share of national food and nutrition governance relies on instruments that may vary in legal force, monitoring requirements, and sanctioning mechanisms. Laws and binding regulations can create clearer obligations for public authorities, food operators, schools, retailers, and other actors, particularly when accompanied by defined enforcement responsibilities and penalties for non-compliance. By contrast, strategies, action plans, programmes, and voluntary or quasi-binding instruments may be valuable for agenda-setting, coordination, and gradual policy development, but their effectiveness may be limited when implementation responsibilities, accountability mechanisms, funding, and monitoring systems are weak. This is particularly relevant in areas such as food labeling, advertising, and reformulation, where voluntary industry self-regulation has frequently been associated with weak standards, limited transparency, and inadequate monitoring, resulting in modest or inconsistent impacts on population dietary behaviors and health outcomes (31–34). Policy adoption alone should therefore not be interpreted as evidence of effective implementation, and future policy development should give greater attention to legal enforceability, institutional accountability, and sustained monitoring (32, 33).

The relative underrepresentation of economic tools and nutrition counseling within healthcare settings highlights two important missed opportunities in national food and nutrition policy. Economic instruments, including taxes, subsidies, price incentives, and procurement-linked affordability measures, can influence food purchasing patterns by changing the relative price and accessibility of healthier and less healthy products. Their limited adoption may reflect political sensitivity around taxation, concerns about regressivity and household costs, administrative complexity, and opposition from affected commercial sectors. These barriers are particularly relevant when fiscal measures are framed narrowly as revenue-generating taxes rather than as part of broader health-oriented policy packages that include transparent use of revenues, protections for lower-income households, and complementary measures to improve access to healthier foods (35). Nutrition counseling in healthcare settings was also rarely identified, despite the potential role of primary care and clinical services in supporting prevention, early risk identification, and long-term management of diet-related non-communicable diseases. Its underuse may reflect workforce constraints, limited reimbursement mechanisms, insufficient nutrition training among health professionals, weak integration of dietitians and nutrition specialists into care pathways, and the limited prioritization of prevention within health systems that remain largely treatment-oriented (8, 36).

Counseling is unlikely to be effective as an isolated intervention, but it can contribute to sustained dietary improvement when embedded within primary care, linked to social and community support, and aligned with healthier food environments. Strengthening these domains therefore requires not only adopting additional policies, but also addressing the political, financial, commercial, and health-system barriers that limit their implementation at scale (37–39).

The evidence supporting early-life interventions, including school meal standards, restrictions on food marketing to children, limits on unhealthy competitive foods in schools, and nutrition education programmes, is well established (7, 8, 36, 40). Schools represent a critical policy setting because they reach children during a period in which dietary preferences, food literacy, and health-related behaviors are still developing. In this review, 18.0% of identified policy actions explicitly targeted children and adolescents, primarily through school food provision and standards, restrictions on marketing to minors, and nutrition education initiatives. These measures can contribute to healthier dietary patterns, reduce exposure to unhealthy food environments, and support prevention of diet-related non-communicable diseases from early life. Recent global evidence also suggests that national school food environment policies remain unevenly adopted, particularly for restrictions on food marketing and competitive food sales in schools, and that many existing policies lack explicit monitoring or enforcement provisions (41). This reinforces the importance of moving beyond policy adoption alone toward stronger implementation design, including clear nutritional standards, limits on commercial promotion in and around schools, monitoring mechanisms, and accountability for compliance. School meal programmes, especially when designed to be universal, nutritious, and sustainable, may also offer wider benefits for food security, social justice, and health equity, particularly among vulnerable populations (42, 43). However, fragmented implementation and unequal access risk reinforcing existing inequalities unless school-based measures are accompanied by explicit equity-oriented strategies and coordinated delivery mechanisms (44–46).

From a policy perspective, the observed imbalance across NOURISHING domains suggests that tailored approaches adapting established best practices to local contexts may enhance both feasibility and acceptability. Gradual fiscal reforms, community-based education initiatives, and structured public–private engagement for product reformulation may help bridge current gaps while maintaining political and social support. At the European Union level, more coordinated action could further harmonize standards, facilitate knowledge exchange, and promote more equitable health outcomes across member states (47).

This study has several limitations that should be acknowledged. While it provides a comprehensive overview of national food and nutrition policy actions across 31 European countries, its geographic focus excludes other global regions, limiting broader generalizability. In addition, the review focused on national-level policy actions and excluded subnational policies implemented at regional, provincial, municipal, or local levels. This exclusion was consistent with the study objective of comparing national policy landscapes across countries, but it may have led to the omission of relevant food and nutrition initiatives implemented by decentralized authorities, particularly in countries where public health, education, school food provision, procurement, or local food-environment regulation are partly governed below the national level. Reliance on publicly available legislative documents may also have resulted in the omission of recent, unpublished, or difficult-to-access policy actions. In addition, the exclusion of records focused solely on sugar-sweetened beverages, due to their recent and extensive mapping elsewhere (15, 48–50), restricts a complete assessment of beverage-specific fiscal measures. Sugar-sweetened beverage policies have documented their growing global adoption and their relevance for obesity, diabetes, and other diet-related health outcomes. In this context, the present review complements rather than duplicates the sugar-sweetened beverage literature by broadening the analytical focus to national food and nutrition policy actions across the wider food system and food environment. This allowed the review to capture policy areas that are less frequently synthesized together, including food labeling, reformulation, school and institutional food standards, marketing restrictions, retail and food service environments, healthcare-based nutrition counseling, nutrition education, and cross-sectoral food system governance.

Finally, variations in policy enforcement and implementation quality were not systematically assessed and may influence real-world impact beyond documented policy adoption.

## Conclusion

National nutrition policies represent indispensable instruments for promoting healthier populations and mitigating diet-related disease burdens. This analysis highlights substantial cross-country heterogeneity and uneven policy coverage across NOURISHING domains, underscoring the need to strengthen governance, enforcement, and the strategic integration of nutrition across sectors and healthcare systems. Addressing persistent gaps, particularly in the use of economic tools and the institutionalization of nutrition services within healthcare, will be critical to advancing sustainable food systems, reducing health inequalities, and fulfilling public health commitments across Europe.
