# Supplementary material for: Shaping food system governance: mapping the national food and nutrition legislation landscape across Europe
Source: Front Public Health. 2026 Jun 5;14:1850537. doi: 10.3389/fpubh.2026.1850537 (PMC13279532; doi:10.3389/fpubh.2026.1850537)
Supplement: Supplementary file 1 [file Data_Sheet_1.docx]

**Supplementary materials**

**Supplementary Table S1. Completed PRISMA-ScR checklist**

| **Section** | **Item** | **PRISMA-ScR checklist item** | **Reported in the manuscript** |
| --- | --- | --- | --- |
| **Title** | 1 | Identify the report as a scoping review. | Title page. The manuscript identifies the study as a scoping review mapping national food and nutrition policy actions across Europe. |
| **Abstract** | 2 | Provide a structured summary that includes, as applicable: background, objectives, eligibility criteria, sources of evidence, charting methods, results, and conclusions that relate to the review questions and objectives. | Abstract. The abstract includes the introduction/background, methods, results, and conclusion. |
| **Introduction / Rationale** | 3 | Describe the rationale for the review in the context of what is already known. Explain why the review questions/objectives lend themselves to a scoping review approach. | Introduction. The manuscript describes the public health relevance of dietary risk factors, the role of policy in shaping food environments, and the need to map national food and nutrition policies across Europe. |
| **Introduction / Objectives** | 4 | Provide an explicit statement of the questions and objectives being addressed with reference to their key elements, such as population or participants, concepts, and context. | Introduction and Methods. The manuscript states the aim of mapping national policies addressing food production, sale, and consumption across European countries and classifying these actions using the NOURISHING framework. In the revised manuscript, the research question is explicitly stated in the Methods section. |
| **Methods / Protocol and registration** | 5 | Indicate whether a review protocol exists; state whether and where it can be accessed; and provide registration information, including registration number, if available. | Methods. The manuscript states that the research protocol was made publicly available on the Open Science Framework platform: DOI 10.17605/OSF.IO/8DXP6. |
| **Methods / Eligibility criteria** | 6 | Specify characteristics of the sources of evidence used as eligibility criteria, such as years considered, language, and publication status, and provide a rationale. | Methods, Eligibility Criteria and Assessment. The manuscript describes inclusion and exclusion criteria, including national-level policy actions, formal enactment and in-force status, country coverage, language, source types, and exclusions such as subnational actions and non-enacted measures. |
| **Methods / Information sources** | 7 | Describe all information sources in the search, such as databases and contact with authors to identify additional sources, as well as the date the most recent search was executed. | Methods, Search strategy and definitions. The manuscript reports searches in PubMed, Scopus, FAOLEX, GIFNA, and Google, covering records published or available between January 2014 and January 2024. |
| **Methods / Search** | 8 | Present the full electronic search strategy for at least one database, including any limits used, such that it could be repeated. | Supplementary Table S1. The full search strategy for Scopus, PubMed, and Google is provided. |
| **Methods / Selection of sources of evidence** | 9 | State the process for selecting sources of evidence, including screening and eligibility assessment. | Methods, Screening of records. The manuscript describes duplicate removal, title/abstract/full-text screening, independent screening of academic records, grey literature screening, and consistency checking. In the revised manuscript, disagreement resolution is clarified. |
| **Methods / Data charting process** | 10 | Describe the methods of charting data from the included sources of evidence and any processes for obtaining and confirming data from investigators. | Methods, Data Extraction & Classification. The manuscript describes the extraction process and the variables charted for each policy action. In the revised manuscript, additional clarification is provided on verification against official or database sources where available. |
| **Methods / Data items** | 11 | List and define all variables for which data were sought and any assumptions or simplifications made. | Methods, Data Extraction & Classification. The manuscript lists extracted variables, including source of reference, source document type, legal nature, year of entry into force, country, description, aim, responsible institution, target population, targeted products, and targeted food components. |
| **Methods / Critical appraisal of individual sources of evidence** | 12 | If done, provide a rationale for conducting a critical appraisal of included sources of evidence; describe the methods used and how this information was used in data synthesis. | Not applicable. A formal critical appraisal was not conducted because this was a scoping review aiming to map national policy actions rather than assess intervention effectiveness or estimate pooled effects. |
| **Methods / Synthesis of results** | 13 | Describe the methods of handling and summarizing the data that were charted. | Methods, Data Extraction & Classification. The manuscript describes classification of policy actions according to legal nature and NOURISHING framework domains. Results are summarized descriptively by country, policy type, target population, targeted products/components, and NOURISHING domain. |
| **Results / Selection of sources of evidence** | 14 | Give numbers of sources of evidence screened, assessed for eligibility, and included in the review, with reasons for exclusions at each stage, ideally using a flow diagram. | Results and Figure 1. The manuscript reports the number of records identified from scientific databases and grey literature sources, the number included after screening, and presents the PRISMA flowchart. |
| **Results / Characteristics of sources of evidence** | 15 | For each source of evidence, present characteristics for which data were charted and provide citations. | Results and Supplementary Table S3. The manuscript summarizes the characteristics of included policy actions, and Supplementary Table S3 provides detailed information on each mapped policy action. In the revised supplementary material, source/reference information is added for each policy action. |
| **Results / Critical appraisal within sources of evidence** | 16 | If done, present data on critical appraisal of included sources of evidence. | Not applicable. No formal critical appraisal of individual sources was conducted. |
| **Results / Results of individual sources of evidence** | 17 | For each included source of evidence, present the relevant data that were charted. | Supplementary Table S3. The supplementary material provides the full list of mapped policy actions and extracted characteristics. |
| **Results / Synthesis of results** | 18 | Summarize and/or present the charting results as they relate to the review questions and objectives. | Results. The manuscript presents the distribution of policy actions by country, legal nature, year of entry into force, target population, targeted products/components, and NOURISHING framework domain. |
| **Discussion / Summary of evidence** | 19 | Summarize the main results, including an overview of concepts, themes, and types of evidence available, and link these to the review questions and objectives. | Discussion. The manuscript summarizes cross-country heterogeneity in national food and nutrition policy actions, highlights domains with broader and weaker coverage, and interprets the findings in relation to food system governance and public health nutrition. |
| **Discussion / Limitations** | 20 | Discuss the limitations of the scoping review process. | Discussion, Limitations. The manuscript discusses limitations related to public/secondary sources, translation and interpretation errors, policy classification, and the formal nature of policy mapping. In the revised manuscript, the exclusion of subnational policies is explicitly added as a limitation. |
| **Discussion / Conclusions** | 21 | Provide a general interpretation of the results with respect to the review questions and objectives, as well as potential implications and/or next steps. | Conclusion. The manuscript concludes that European countries share some baseline regulation but differ substantially in the breadth and strength of food and nutrition policy actions, with gaps in key areas requiring further policy attention. |
| **Funding** | 22 | Describe sources of funding for the included sources of evidence, as well as sources of funding for the scoping review. Describe the role of funders of the scoping review. | Funding statement. The manuscript reports support from the 4P-CAN project, funded under Horizon Europe, and includes the funding information in the declarations section. |

**Supplementary Table S2.** The complete search strategy.

| **Database** | **Search Fields** | **Search String** |
| --- | --- | --- |
| Scopus | TITLE-ABS, INDEXTERMS | (TITLE-ABS("systematic review") OR TITLE-ABS("scoping review") OR TITLE-ABS("narrative review") OR TITLE-ABS("literature review") OR TITLE-ABS("umbrella review")) AND (TITLE-ABS(food) OR TITLE-ABS(nutrition) OR TITLE-ABS("dietary pattern") OR TITLE-ABS("food additives") OR TITLE-ABS("eating behaviour") OR TITLE-ABS("unhealthy eating behaviour") OR TITLE-ABS("healthy eating behaviour") OR TITLE-ABS("eating habits")) AND (TITLE-ABS(policy) OR TITLE-ABS(policies) OR TITLE-ABS(ban) OR TITLE-ABS(banned) OR TITLE-ABS(restriction) OR TITLE-ABS(restrictions) OR TITLE-ABS(law) OR TITLE-ABS(laws) OR TITLE-ABS(reform) OR TITLE-ABS(reforms) OR TITLE-ABS(legislation) OR TITLE-ABS(legislations) OR TITLE-ABS(regulation) OR TITLE-ABS(regulations) OR TITLE-ABS(restrict) OR TITLE-ABS(prohibit) OR TITLE-ABS(prohibition) OR TITLE-ABS(prohibitions) OR TITLE-ABS(ordinance) OR TITLE-ABS(tax) OR TITLE-ABS(taxes) OR TITLE-ABS(taxing) OR TITLE-ABS(incentive) OR TITLE-ABS(excise) OR TITLE-ABS(fiscal) OR TITLE-ABS(levied) OR TITLE-ABS(levy) OR TITLE-ABS(price) OR TITLE-ABS(priced) OR TITLE-ABS(prices) OR TITLE-ABS(pricing) OR TITLE-ABS(subsidy) OR TITLE-ABS(subsidies) OR INDEXTERMS("fiscal policy") OR INDEXTERMS("health policy")) |
| PubMed | Title/Abstract, MeSH Terms | ("systematic review"[Title/Abstract] OR "scoping review"[Title/Abstract] OR "narrative review"[Title/Abstract] OR "literature review"[Title/Abstract] OR "umbrella review"[Title/Abstract]) AND (food[Title/Abstract] OR nutrition[Title/Abstract] OR "dietary pattern"[Title/Abstract] OR "food additives"[Title/Abstract] OR "eating behaviour"[Title/Abstract] OR "unhealthy eating behaviour"[Title/Abstract] OR "healthy eating behaviour"[Title/Abstract] OR "eating habits"[Title/Abstract]) AND (policy[Title/Abstract] OR policies[Title/Abstract] OR ban[Title/Abstract] OR banned[Title/Abstract] OR restriction[Title/Abstract] OR restrictions[Title/Abstract] OR law[Title/Abstract] OR laws[Title/Abstract] OR reform[Title/Abstract] OR reforms[Title/Abstract] OR legislation[Title/Abstract] OR legislations[Title/Abstract] OR regulation[Title/Abstract] OR regulations[Title/Abstract] OR restrict[Title/Abstract] OR prohibit[Title/Abstract] OR prohibition[Title/Abstract] OR prohibitions[Title/Abstract] OR ordinance[Title/Abstract] OR tax[Title/Abstract] OR taxes[Title/Abstract] OR taxing[Title/Abstract] OR incentive[Title/Abstract] OR excise[Title/Abstract] OR fiscal[Title/Abstract] OR levied[Title/Abstract] OR levy[Title/Abstract] OR price[Title/Abstract] OR priced[Title/Abstract] OR prices[Title/Abstract] OR pricing[Title/Abstract] OR subsidy[Title/Abstract] OR subsidies[Title/Abstract] OR "fiscal policy"[MeSH Terms] OR "health policy"[MeSH Terms]) |
| Google* | General web search terms | (Food OR nutrition) AND (policy OR policies OR ban OR banned OR restriction OR restrictions OR law OR laws OR reform OR reforms OR legislation OR regulations OR programmes OR actions OR ordinance OR decree OR tax OR taxes OR price OR prices OR “national strategy” OR “nutrition action plan” OR “food safety” OR “food labelling” OR “school meals” OR “public health nutrition”) AND [country name] |

***Note:** For Google searches, the general search string was combined with the name of each included country. Additional country-specific searches were conducted where needed using terms such as “national strategy,” “law,” “regulation,” “decree,” “ordinance,” “nutrition action plan,” “food safety,” “food labelling,” “school meals,” and “public health nutrition.” Search results were screened until no additional eligible national-level records were identified.

**Supplementary Table S3.** List of mapped national food and nutrition policy actions by country, extracted characteristics, and source references (N = 379)

| **ID (ISO Code_n)** | **Main Policy area (NOURISHING)** | **Policy nutrition action name** | **Year of entry into force** | **Legal Nature** | **Description of the main content** | **Aim of the legislation** | **Targeted products** | **Targeted Food components** | **Involving children/adolescent** | **Source / Reference** | **Source Type** |
| --- | --- | --- | --- | --- | --- | --- | --- | --- | --- | --- | --- |
| **AT_1** | Set incentives and rules to create a healthy retail and food service environment | Food Safety and Consumer Protection Act | 2006 | Law | This legislation protects health and consumer interests throughout food production, processing, and distribution, excluding private domestic activities. | To safeguard consumer health and prevent deception by enforcing relevant EU laws. | All food products | Not specified |  | [FAOLEX record: Food Safety and Consumer Protection Act](https://www.fao.org/faolex/results/details/en/c/LEX-FAOC089320) | FAOLEX specific legal record |
| **AT_2** | Improve the nutritional quality of the food supply | Austria trans fatty acids regulation | 2009 | Other (regulation) | The regulation limits Trans Fatty Acids to 2% of total fat content, with multi-ingredient processed foods under 20% fat limited to 4%, and those under 3% fat limited to 10% | To reduce TFA content in food products. | All food products | Trans Fatty Acids |  | [FAOLEX record: Fatty Acid Ordinance](https://www.fao.org/faolex/results/details/en/c/LEX-FAOC090973) | FAOLEX specific legal record |
| **AT_3** | Improve the nutritional quality of the food supply | Less salt is healthier initiative | 2010 | Policy | The policy reduces salt in bread and baked goods by 15% by 2015, with 113 bakery businesses and over 350 branches committed to this reduction. | To reduce the salt content in bread and baked goods. | Bread/bakery products | Salt/sodium |  | [WCRF NOURISHING entry: Less Salt is Healthier programme](https://policydatabase.wcrf.org/wcrf_nourishing_moving/ajax/52536/52536//Salt/0/0/accordion_data?database=0) | WCRF NOURISHING policy database entry |
| **AT_4** | Harness supply chain and actions across sectors to ensure coherence with health | National Nutrition Action Plan (NAP.e) | 2011 | Policy | The policy aims to reduce obesity, promote access to nutritious food, and integrate nutrition into healthcare through collaboration. | To improve public health by promoting better diets and reducing diet-related diseases. | All food products | Not specified |  | [WHO/NCDCCS PDF: Austrian National Nutrition Action Plan](https://extranet.who.int/ncdccs/Data/AUT_B13_nape_kurzfassung_englisch_110103.pdf) | WHO/NCDCCS hosted policy PDF |
| **BE_1** | Nutrition label standards and regulations on the use of claims and implied claims on food | Arrêté royal relatif à la crème. | 1934 | Other (decree) | The decree regulates the classification and labeling of cream products. | To outline specific definitions for various types of cream based on their fat content percentage and prohibiting those that contain antiseptic. | Cream products | Fat |  | [FAOLEX record: Arrêté royal relatif à la crème](https://www.fao.org/faolex/results/details/en/c/LEX-FAOC203636) | FAOLEX specific legal record |
| **BE_2** | Set incentives and rules to create a healthy retail and food service environment | Loi relative à la protection de la santé de consommateurs en ce qui concerne les denrées alimentaires et les autres produits. | 1977 | Law | This law of January 24, 1977 on consumer health protection with regard to foodstuffs and other products defines certain terms, such as “foodstuffs”, “other products”, “trade or placing on the market”, “manufacture or fabrication”, and so on. | The law is aimed at protecting public health, ensuring food safety, preventing fraud and ensuring that consumers receive correct and complete information on food products. | All food products | Not specified |  | [FAOLEX record: Law on consumer health protection concerning foodstuffs](https://www.fao.org/faolex/results/details/en/c/LEX-FAOC010731) | FAOLEX specific legal record |
| **BE_3** | Set incentives and rules to create a healthy retail and food service environment | Royal Decree of 2 September 1985 on bread and other bakery products | 1985 | Other (decree) | This royal decree limits salt in bread to 2% based on dry matter (1.7% on flour or 1.2-1.4% in the final product), excluding imported bread. | To reduce the content of salt in foodstuff. | Bread/bakery products | Salt/sodium |  | [Refli/Justel record: Royal Decree of 2 September 1985 on bread and bakery products](https://refli.be/fr/lex/1985013286) | Official Belgian legal text database record |
| **BE_4** | Harness supply chain and actions across sectors to ensure coherence with health | Arrêté royal du 25 novembre 1991 concernant les solvants d'extraction utilisés dans la fabrication des denrées alimentaires. | 1991 | Other (decree) | This decree regulates extraction solvents used in food manufacturing but excludes those for food additives, vitamins, and nutrients unless specified in the annex. | To regulate the use of extraction solvents in food manufacturing, prohibiting harmful substances within the EU. | All food products | Extraction solvents |  | [FAOLEX record: Royal Decree on extraction solvents used in food production](https://www.fao.org/faolex/results/details/en/c/LEX-FAOC009790) | FAOLEX specific legal record |
| **BE_5** | Nutrition label standards and regulations on the use of claims and implied claims on food | Arrêté ministériel déterminant les formes chimiques et les critères de pureté pour les nutriments qui peuvent être utilisés pour des compléments alimentaires. | 2003 | Other (decree) | This decree specifies the authorized chemical forms and purity criteria for nutrients used in food supplements. | To ensure dietary supplements meet specific safety and quality standards. | dietary supplements | Nutrients |  | [FAOLEX record: Ministerial Decree on chemical forms/purity criteria for nutrients](https://www.fao.org/faolex/results/details/en/c/LEX-FAOC038636) | FAOLEX specific legal record |
| **BE_6** | Harness supply chain and actions across sectors to ensure coherence with health | Arrêté royal relatif à l'autocontrôle, à la notification obligatoire et à la traçabilité dans la chaîne alimentaire. | 2003 | Other (regulation) | The regulation was designed to protect public health by ensuring that food products are safe, properly labeled, and traceable throughout the food supply chain. | The legislation aims to enhance food safety by establishing comprehensive measures for self-monitoring, mandatory reporting, and traceability within the food supply chain. | All food products | Not specified |  | [FAOLEX record: Royal Decree on self-checking, mandatory notification and traceability](https://www.fao.org/faolex/results/details/en/c/LEX-FAOC044346) | FAOLEX specific legal record |
| **BE_7** | Harness supply chain and actions across sectors to ensure coherence with health | Arrêté royal fixant des mesures complémentaires pour l'organisation des contrôles officiels concernant les produits d'origine animale destinés à la consommation humaine. | 2005 | Other (regulation) | The regulation establishes specific rules for official controls on animal origin products intended for human consumption. | To protect public health by ensuring animal products are safely inspected and free from contaminants before reaching consumers. | Animal-origin products | Not specified |  | [FAOLEX record: Royal Decree on official controls of animal-origin products](https://www.fao.org/faolex/results/details/en/c/LEX-FAOC061179) | FAOLEX specific legal record |
| **BE_8** | Inform people about food and nutrition through public awareness | Policy for promotion of healthy nutritional habits and physical activity of children and adolescents | 2005 | Policy | This policy emphasizes the importance of education, community involvement, and coordinated efforts among families, schools, and local authorities to create a supportive environment that promotes long-term health and critical awareness of media and commercial influences. | To enhance the well-being of children and adolescents by promoting healthy eating habits, regular physical activity, and critical awareness of commercial influences. | All food products | Not specified | X | [WHO NLIS country profile: healthy nutrition and physical activity policy for children/adolescents](https://apps.who.int/nutrition/landscape/report.aspx?iso=bel&print=1) | WHO NLIS policy record |
| **BE_9** | Restrict food advertising and other forms of commercial promotion | Decree on advertising to children | 2007 | Other (decree) | The decree prohibits advertisements targeting children and young people from encouraging or justifying the excessive consumption of unhealthy foods. | To protect children from being influenced by advertisements that promote or normalize excessive consumption of foods containing fats, trans fats, salt or sodium, and sugar. | Unhealthy food products | fats, trans fats, salt or sodium, and sugar | X | [Belgium Fevia Food Advertising Code PDF](https://www.g-regs.com/downloads/BEFoodandDrinkFeviaCode.pdf) | Sectoral food advertising code / policy document |
| **BE_10** | Nutrition label standards and regulations on the use of claims and implied claims on food | Nutri-Score Labeling Policy | 2019 | Policy | The voluntary Nutri-Score front-of-pack nutrition labelling system represents a front-of-pack labelling system providing a synthetic information system based on colours and letters (from green/A to red/E). | To allow consumers to see and compare at a glance the nutritional value of pre-packaged foodstuffs. | All food products | Nutrients |  | [Belgian FPS Public Health: Royal Decree on use of the Nutri-Score logo](https://www.health.belgium.be/fr/organisation-politique/reglementation-documents-politique/arrete-royal-1-mars-2019) | Official government regulation page |
| **BE_11** | Harness supply chain and actions across sectors to ensure coherence with health | Arrêté du Gouvernement flamand relatif à la production biologique et à l'étiquetage des produits biologiques. | 2021 | Other (decree) | The decree regulates all aspects of organic production, preparation, and distribution, including catering, setting standards for soil management, pest control, animal welfare, and labeling to ensure consumer trust. | To ensure organic products meet EU standards for quality, traceability, and integrity. | Organic products | Not specified |  | [FAOLEX record: Flemish Government Decree on organic production and labelling](https://www.fao.org/faolex/results/details/en/c/LEX-FAOC207282) | FAOLEX specific legal record |
| **BE_12** | Harness supply chain and actions across sectors to ensure coherence with health | Arrêté royal concernant la mise dans le commerce de nutriments et de denrées alimentaires auxquelles des nutriments ont été ajoutés. | 2021 | Other (decree) | The Royal Decree of May 30, 2021, establishes regulations for nutrients and nutrient-enriched foods in Belgium. | To set on the market of nutrients and foods to which nutrients have been added. | All food products | Nutrients |  | [FAOLEX record: Royal Decree on nutrients and foods with added nutrients](https://www.fao.org/faolex/results/details/en/c/LEX-FAOC203679) | FAOLEX specific legal record |
| **BE_13** | Nutrition label standards and regulations on the use of claims and implied claims on food | Arrêté royal relatif à la fabrication et au commerce de compléments alimentaires contenant d'autres substances que des nutriments et des plantes ou des préparations de plantes. | 2021 | Other (regulation) | The regulation sets strict standards for the manufacturing and sale of food products with plant ingredients, covering production, hygiene, labeling, and safety. | To ensure safety and compliance in their respective areas/one for dietary supplements and the other for food products containing plant ingredients. | All food products | Plant ingredients. |  | [FAOLEX record: Royal Decree on dietary supplements containing other substances](https://www.fao.org/faolex/results/details/en/c/LEX-FAOC206944) | FAOLEX specific legal record |
| **BE_14** | Harness supply chain and actions across sectors to ensure coherence with health | Arrêté royal relatif au contrôle de la qualité du lait cru et à l'agrément des organismes interprofessionnels. | 2021 | Other (regulation) | It sets standards for the control of raw milk quality and accredits interprofessional organizations. It mandates procedures for sampling and transporting milk samples to ensure food chain safety in line with EU regulations. | To establish a robust framework for the official control of raw milk quality, ensuring food safety within the dairy supply chain. | Milk | Not specified |  | [FAOLEX record: Royal Decree on raw milk quality control](https://www.fao.org/faolex/results/details/en/c/LEX-FAOC205762) | FAOLEX specific legal record |
| **BG_1** | Set incentives and rules to create a healthy retail and food service environment | National Food and Nutrition Action Plan (NFNAP) 2005-2010 | 2005 | Policy | The NFNAP focuses on three key areas: (i) Nutrition: Promoting healthier dietary habits and improving nutritional standards for the entire population; (ii) Food Safety: Ensuring safe practices in food production, handling, and consumption to prevent foodborne diseases; (iii) Food Security: Ensuring reliable access to adequate, safe, and nutritious food for all. | The NFNAP aims to reduce foodborne illnesses and diet-related chronic diseases through a comprehensive and coordinated approach. | All food products | Not specified |  | [Bulgarian Journal of Public Health PDF: Food and Nutrition Action Plan 2005-2010](https://ncpha.government.bg/uploads/pages/3020/BGJPH_2012_02.pdf) | National public health institute journal / policy documentation |
| **BG_2** | Offer healthy food and set standards in public institutions and other specific settings | Ordinance No. 37 of july 21, 2009 on healthy feeding of pupils | 2009 | Other (ordinance) | This ordinance outlines the requirements for providing healthy nutrition in various school related settings. It applies to school cafeterias, buffets, vending machines, and specialized institutions for social services. | To regulate and ensure the provision of healthy and balanced nutrition for students within school environments. | School food products | Nutrients | X | [JRC School Food Policy Factsheet: Bulgaria](https://joint-research-centre.ec.europa.eu/system/files/2017-07/jrc-school-food-policy-factsheet-bulgaria_en.pdf) | European Commission JRC policy factsheet |
| **BG_3** | Improve the nutritional quality of the food supply | Bulgarian white brined cheese 2010 | 2010 | Policy | This policy establishes the specific physicochemical standards and production processes for Bulgarian white brined cheese, ensuring consistent quality and safety in its production. This regulates aspects such as fat content, salt levels, and energy value. | This policy aims to outline the physicochemical parameters and technological processes required to produce Bulgarian white brined cheese. The policy also details the energy value of the cheese, and the salting process. | White brined cheese. | Fat, salt, and energy value |  | [Scientific article documenting Bulgarian National Standard BDS 15:2010](https://pmc.ncbi.nlm.nih.gov/articles/PMC7996614/) | Peer-reviewed source documenting national standard |
| **BG_4** | Improve the nutritional quality of the food supply | Standards for bread and flour | 2011 | Policy | This policy specifies the maximum allowable level of culinary salt in food products, stating that it should not exceed 1 gram per 100 grams of the product. | The policy aims to control the salt content in food items, thereby contributing to better public health outcomes and adherence to nutritional guidelines. | All food products | Salt |  | [AgroDealsEU standards page: Standards for bread and flour “Bulgaria”](https://agrodealseu.net/knowledge-and-tips/standards) | Policy/standards information page |
| **BG_5** | Offer healthy food and set standards in public institutions and other specific settings | National Food and Nutrition Action Plan: Ordinance for healthy nutrition in school | 2012 | Other(ordinance) | This ordinance sets mandatory nutritional standards for food provided to school-aged children in canteens and other facilities, such as accommodation and sports centers. It specifies maximum salt limits for various foods, including bread, cheeses, meat products, and traditional Bulgarian foods like lutenica. | To lower the salt content in food provided within the school environment. | Bread, cheeses, meat products, and traditional foods. | Salt | X | [JRC School Food Policy Factsheet: Bulgaria](https://joint-research-centre.ec.europa.eu/system/files/2017-07/jrc-school-food-policy-factsheet-bulgaria_en.pdf) | European Commission JRC policy factsheet |
| **BG_6** | Harness supply chain and actions across sectors to ensure coherence with health | Bulgarian Law on food | 2020 | Law | The law covers food safety and sets out the responsibilities of food business operators and workers in production, processing, and distribution. It sets standards for packaging, labeling, advertising, and production practices, including Hazard Analysis and Critical Control Points (HACCP). | This law aims to ensure a high level of protection of the health and interests of consumers with regard to food; ensure the application of laws of the European Union and of national measures in food sector. | All food products | Not specified |  | [FAOLEX record: Bulgarian Food Law](https://www.fao.org/faolex/results/details/en/c/LEX-FAOC201800) | FAOLEX specific legal record |
| **BG_7** | Harness supply chain and actions across sectors to ensure coherence with health | Law on the management of the agro-food chain. | 2020 | Law | This legislation oversees plant health, animal health, veterinary medicines, feed, food safety, GMOs (excluding environmental release), and biological production. These collaborate to ensure compliance with EU regulations, maintain food safety, and protect consumer interests. | To regulate and ensure the safety, quality, and traceability of agricultural and food products throughout the production, distribution, and marketing processes. | All food products | Not specified |  | [FAOLEX record: Law on the Management of the Agri-Food Chain](https://www.fao.org/faolex/results/details/en/c/LEX-FAOC207010) | FAOLEX specific legal record |
| **BG_8** | Set incentives and rules to create a healthy retail and food service environment | Decree No. 170 of 23 April 2021 on the adoption of an Ordinance on the requirements for certain partially or completely dehydrated milk intended for human consumption. | 2021 | Other (decree) | This decree establishes specific criteria regarding names, composition, characteristics, labeling, sampling methods, and analytical procedures for certain types of dehydrated milk meant for human consumption. | The aim ot this decree is to adopt regulations for specific partially or completely dehydrated milk intended for human consumption. | Dehydrated milk | Not specified |  | [FAOLEX record: Decree No. 170 on dehydrated milk](https://www.fao.org/faolex/results/details/en/c/LEX-FAOC202459) | FAOLEX specific legal record |
| **BG_9** | Set incentives and rules to create a healthy retail and food service environment | Decree No. 248 of 26 July 2021 on the adoption of an Ordinance on specific requirements for caseins and caseinates intended for human consumption. | 2021 | Other (decree) | This Decree enacts Regulations governing precise criteria for caseins and caseinates meant for human consumption, derived from the provisions of the national Food Law. | To stipulate specific standards concerning names, composition, attributes, labeling, sampling methods for chemical analysis, and analysis techniques for composition. | Creams products | Caseins and caseinates |  | [FAOLEX record: Decree No. 248 on caseins and caseinates](https://www.fao.org/faolex/results/details/en/c/LEX-FAOC204322) | FAOLEX specific legal record |
| **BG_10** | Set incentives and rules to create a healthy retail and food service environment | Decree No. 260 of 29 July 2021 on the adoption of an Ordinance on specific requirements for dairy products. | 2021 | Other (decree) | The decree outlines detailed regulations concerning the production, labeling, and trade of dairy products in Bulgaria. | The aim of the decree is to set out conditions to protect consumers and maintain product integrity in the market. | Dairy products | Not specified |  | [FAOLEX record: Decree No. 260 on dairy products](https://www.fao.org/faolex/results/details/en/c/LEX-FAOC204320) | FAOLEX specific legal record |
| **BG_11** | Improve the nutritional quality of the food supply | Decree No. 302 of 17 September 2021 on the adoption of an Ordinance on the requirements for coffee and chicory extracts. | 2021 | Other (decree) | The decree establishes regulations on the production, labeling, and quality standards for coffee and chicory extracts (% dry matter by weight). | To establish requirements for the labeling, composition, and analysis methods of coffee and chicory extracts. | Coffee and chicory extracts. | Not specified |  | [FAOLEX record: Decree No. 302 on coffee and chicory extracts](https://www.fao.org/faolex/results/details/en/c/LEX-FAOC205165) | FAOLEX specific legal record |
| **BG_12** | Improve the nutritional quality of the food supply | Decree No. 367 of 19 October 2021 adopting the Ordinance on the requirements for cocoa and chocolate products. | 2021 | Other (decree) | The Ordinance shall determine the requirements for the names, composition, characteristics and labelling of cocoa and chocolate products. | To establish comprehensive regulations governing the production, processing, labeling, and commercialization of cocoa and chocolate products. | Cocoa and chocolate products (e.g chcolate bars, baked goods) | Cocoa and chocolate |  | [FAOLEX record: Decree No. 367 on cocoa and chocolate products](https://www.fao.org/faolex/results/details/en/c/LEX-FAOC207029) | FAOLEX specific legal record |
| **BG_13** | Nutrition label standards and regulations on the use of claims and implied claims on food | Decree No. 97 of 19 March 2021 on the adoption of an Ordinance on the provision of information to consumers about food. | 2021 | Other (decree) | The adopted Ordinance shall determine: 1. batch identification requirements for food; 2. national measures for consumer food information, including packaging and labeling. | Decree on the adoption of an Ordinance on the provision of information to consumers about food. | All food products | Not specified |  | [FAOLEX record: Decree No. 97 on food information to consumers](https://www.fao.org/faolex/results/details/en/c/LEX-FAOC201767) | FAOLEX specific legal record |
| **BG_14** | Improve the nutritional quality of the food supply | Ordinance No. 10 of 26 April 2021 on specific requirements to produce food of animal origin in slaughterhouses. | 2021 | Other(ordinance) | This ordinance defines specific requirements to produce food of animal origin in slaughterhouses, these provisions shall apply to large and small ruminants, and some mammals. | The ordinance sets out specific requirements to produce food of animal origin in slaughterhouses. | Animal-origin products | Not specified |  | [FAOLEX record: Ordinance No. 10 on animal-origin food in slaughterhouses](https://www.fao.org/faolex/results/details/en/c/LEX-FAOC202457) | FAOLEX specific legal record |
| **BG_15** | Harness supply chain and actions across sectors to ensure coherence with health | Ordinance No. 11 of 7 June 2021 on terms and conditions for processing foods with ionizing radiation and requirements for them. | 2021 | Other(ordinance) | This ordinance regulates the processing of food using ionizing radiation. It outlines maximum permissible doses of radiation, procedural requirements, and mandates labeling of irradiated foods. | To set terms and conditions for processing foods with ionizing radiation and requirements for them. | Irradiated food products | Not specified |  | [FAOLEX record: Ordinance No. 11 on foods processed with ionizing radiation](https://www.fao.org/faolex/results/details/en/c/LEX-FAOC204059) | FAOLEX specific legal record |
| **BG_16** | Harness supply chain and actions across sectors to ensure coherence with health | Ordinance No. 5 of 9 February 2021 on the authorization for the placing on the market and use of plant protection products. | 2021 | Other(ordinance) | This ordinance regulates the conditions and procedures for: authorizing the placement and use of plant protection products (PPP), and renewing or modifying permits for PPP. | Ordinance on the authorization for the placing on the market and use of plant protection products. | Plant protection products | Not specified |  | [ECOLEX record: Ordinance No. 5 on plant protection products](https://www.ecolex.org/details/legislation/ordinance-no-5-of-9-february-2021-on-the-authorization-for-the-placing-on-the-market-and-use-of-plant-protection-products-lex-faoc200849/) | ECOLEX/FAOLEX specific legal record |
| **BG_17** | Harness supply chain and actions across sectors to ensure coherence with health | Ordinance No. 8 of 23 February 2021 on the terms and conditions for control over plant protection products, trade, repackaging, storage and use. | 2021 | Other(ordinance) | This ordinance sets the conditions and procedures for controlling plant protection products (PPP) in terms of their general use, trade, repackaging, storage, and application. | This ordinance, in compliance with the Bulgarian Law on Plant Protection, aims to set control over plant protection products, trade, repackaging, storage and use. | Plant protection products | Not specified |  | [FAOLEX record: Ordinance No. 8 on plant protection products control](https://www.fao.org/faolex/results/details/en/c/LEX-FAOC201169) | FAOLEX specific legal record |
| **BG_18** | Offer healthy food and set standards in public institutions and other specific settings | Regulation No– 2 of 20 January 2021 on the specific requirements for the safety and quality of food offered in kindergartens, school canteens and retail outlets on the territory of schools and kindergartens, as well as for food offered at organized events for children and students. | 2021 | Other (regulation) | This regulation defines the specific requirements for the safety and quality of food offered in childcare facilities, school canteens and in points of sale in the territory of schools and childcare facilities. | To ensure the safety and quality of food provided to children and young students in childcare facilities, school canteens, and at events. | School food products | Not specified | x | [FAOLEX record: Regulation No. 2 on food safety and quality in kindergartens and schools](https://www.fao.org/faolex/results/details/en/c/LEX-FAOC200267) | FAOLEX specific legal record |
| **BG_19** | Harness supply chain and actions across sectors to ensure coherence with health | Bulgaria National Development Programme 2030. | 2021 | Policy | Strategic national development program defining Bulgaria's goals and priorities specifically related to food security, safety standards, agricultural sustainability, and nutrition until 2030. | Guide medium- and long-term development policies across all government sectors. | All food products | Not specified |  | [Ministry of Finance PDF: National Development Programme BULGARIA 2030](https://www.minfin.bg/upload/46720/National%2BDevelopment%2BProgramme%2BBULGARIA%2B2030.pdf) | Official government policy PDF |
| **BG_20** | Improve the nutritional quality of the food supply | Decree No. 42 of 1 April 2022 on the adoption of an Ordinance on the requirements for the use of extraction solvents in the production of food and food ingredients. | 2022 | Other (decree) | This regulation establishes guidelines for the use of extraction solvents in the production of food and food ingredients. | To ensure the safety of food and food ingredients by regulating the use of extraction solvents. | All food products | Extraction solvents |  | [FAOLEX record: Decree No. 42 on extraction solvents](https://www.fao.org/faolex/results/details/en/c/LEX-FAOC208565) | FAOLEX specific legal record |
| **BG_21** | Nutrition label standards and regulations on the use of claims and implied claims on food | Decree No. 18 of 2 February 2023 on the adoption of an Ordinance on the requirements for certain sugars intended for human consumption. | 2023 | Other (decree) | The Ordinance defines necessary requirements as regards the names, characteristics, composition and quality, labelling and methods of analysis of certain sugars intended for human consumption. | To ensure food quality and labelling in relation to sugar content. | All food products | Sugar |  | [FAOLEX record: Decree No. 18 on sugars intended for human consumption](https://www.fao.org/faolex/results/details/en/c/LEX-FAOC214522) | FAOLEX specific legal record |
| **HR_1** | Nutrition label standards and regulations on the use of claims and implied claims on food | Ordinance on the indication of nutritional values ​​of food | 2005 | Other(ordinance) | This ordinance mandates nutritional information only for foods with nutritional claims on their labels, packaging, or advertising. | To ensure foods with nutritional claims provide standardized information, helping consumers make informed choices | Food products with nutritional claims on their labels | Nutrients |  | [Narodne novine: Pravilnik o navođenju hranjivih vrijednosti hrane (NN 29/2009)](https://narodne-novine.nn.hr/clanci/sluzbeni/2009_03_29_644.html) | Official gazette legal text |
| **HR_2** | Harness supply chain and actions across sectors to ensure coherence with health | Regulation on Food for Infants and Toddlers and Processed Cereal Based Food for Infants and Toddlers | 2008 | Other (regulation) | This Regulation sets out conditions for these products to ensure they are nutritionally appropriate for infants transitioning from breastfeeding and for young children as they gradually adjust to a regular diet. | To ensure that the composition, labelling, and advertising of initial and follow-on formulas, processed cereal-based foods, and baby foods meet specific standards. | Infant food products | Nutrients | X | [FAOLEX record: Regulation on baby food for infants and young children and processed cereal-based food](https://www.fao.org/faolex/results/details/en/c/LEX-FAOC128446) | FAOLEX specific legal record |
| **HR_3** | Inform people about food and nutrition through public awareness | Normative for nutrition of students in primary school | 2013 | Policy | This policy provides age- and gender-based recommendations for daily energy, nutrient, vitamin, and mineral intake to help plan school nutrition. It includes guidelines on meal timing and energy distribution, meal frequency based on school hours, and recommended food types per meal period. | To provide comprehensive guidelines for ensuring proper nutrition for students during their time at primary school. | School food products | Nutrients | X | [Narodne novine: Normativi za prehranu učenika u osnovnoj školi (NN 146/2012)](https://narodne-novine.nn.hr/clanci/sluzbeni/2012_12_146_3164.html) | Official gazette policy/standard text |
| **HR_4** | Improve the nutritional quality of the food supply | Strategic Plan for Salt Intake Reduction. | 2015 | Policy | The Strategic Plan set goals to reduce average daily salt intake from 11.6 grams to 9.3 grams by 2019, representing an average annual reduction of 4%. | This plan aims to significantly decrease the prevalence of hypertension and other cardiovascular and cerebrovascular diseases by reducing the level of salt intake. | All food products | Salt |  | [WHO NLIS/Gina country profile: Strategic Plan for Salt Intake Reduction](https://apps.who.int/nutrition/landscape/report.aspx?iso=hrv&print=1) | WHO NLIS/Gina policy record |
| **HR_5** | Set incentives and rules to create a healthy retail and food service environment | National Strategy for the Implementation of School Fruit and Vegetable Scheme | 2017 | Policy | The scheme targets nearly 450,000 students, including 290,000 in primary and 160,000 in secondary schools. It focuses on providing fresh, locally sourced fruits and vegetables during the school year, excluding products with added sugars or unhealthy additives. | To improve children's eating habits and raise public awareness about the importance of healthy nutrition for their development. The scheme seeks to enhance dietary patterns and foster a greater appreciation for nutritious foods. | School food products | Nutrients | X | [WHO NLIS/Gina country profile: National Strategy for the Implementation of School Fruit and Vegetable Scheme](https://apps.who.int/nutrition/landscape/report.aspx?iso=hrv&print=1) | WHO NLIS/Gina policy record |
| **HR_6** | Improve the nutritional quality of the food supply | Ordinance on Cereals and Cereal Products | 2018 | Other(ordinance) | The ordinance stipulates that the salt content in baked bread that is prepared and ready for consumption must not exceed 1.4% of the total weight of the bread. | To ensure that bread products contribute to a healthier diet by limiting excessive salt intake, reducing the risk of diet-related health problems. | Bread | Salt |  | [FAOLEX record: Regulation on cereals, bakery products, pasta and pasta products](https://www.fao.org/faolex/results/details/en/c/LEX-FAOC123557) | FAOLEX specific legal record |
| **HR_7** | Harness supply chain and actions across sectors to ensure coherence with health | Regulation on edible oils and fats | 2019 | Other (regulation) | This Regulation prescribes the food quality requirements in the production and placing on the market of edible oils of plant origin and of fats of plant and animal origin, including dietary supplements. | To establish and prescribe the quality standards and requirements for the production and marketing of edible oils and fats of both plant and animal origin in Croatia. | Plant and animal origin food products | Edible oils and fats. |  | [FAOLEX record: Regulation on edible oils and fats](https://www.fao.org/faolex/results/details/en/c/LEX-FAOC184776) | FAOLEX specific legal record |
| **HR_8** | Improve the nutritional quality of the food supply | Regulation on fruit jams, jellies, marmalades and sweetened chestnut puree. | 2019 | Other (regulation) | This Regulation prescribes the quality requirements which must be met, in terms of production, composition, names, labeling and placing on the market, by all sweet fruit preserves and spreads. | The aim of this regulation is to establish the quality standards and requirements for the production and marketing of fruit jams and similars. | Fruit jams and spreads | Sweet fruit preserves |  | [FAOLEX record: Regulation on fruit jams, jellies, marmalades and sweetened chestnut puree](https://www.fao.org/faolex/results/details/en/c/LEX-FAOC190436) | FAOLEX specific legal record |
| **HR_9** | Harness supply chain and actions across sectors to ensure coherence with health | Regulation determining the composition of raw milk. | 2020 | Other (regulation) | This determines the chemical composition, physical properties and quality of raw milk from domestic animals used in the production of drinking milk and dairy products. | To regulate the determination of the composition of raw milk. | Raw milk | Not specified |  | [FAOLEX record: Regulation determining the composition of raw milk](https://www.fao.org/faolex/results/details/en/c/LEX-FAOC200188) | FAOLEX specific legal record |
| **HR_10** | Harness supply chain and actions across sectors to ensure coherence with health | Regulation on conditions for monitoring, labelling and packaging of genetically modified organisms and products consisting of or containing genetically modified organisms or a combination of genetically modified organisms. | 2020 | Other (regulation) | This Regulation prescribes objectives and the general principles for the purpose of drawing up plans for monitoring, labelling and packaging of genetically modified organisms (GMOs). | To establish clear objectives and principles for the effective monitoring, labeling, and packaging of genetically modified organisms (GMOs) to ensure safety and transparency. | Genetically modified products | Genetically modified organisms |  | [FAOLEX record: Regulation on conditions for monitoring, labelling and packaging of GMOs and GMO products](https://www.fao.org/faolex/results/details/en/c/LEX-FAOC200578) | FAOLEX specific legal record |
| **HR_11** | Nutrition label standards and regulations on the use of claims and implied claims on food | Regulation on the national quality system of agricultural and food products "Proven quality". | 2020 | Other (regulation) | "Proven quality" is a national quality system intended for labeling agricultural and food products with special characteristics in multiple sectors of food supply chain. (Specifications from Article 99 paragraph 1 of the Act). | To provide a labelling system for food agricultural products. | Agricultural food products. | Not specified |  | [FAOLEX record: Regulation on the national quality system of agricultural and food products "Proven quality"](https://www.fao.org/faolex/results/details/en/c/LEX-FAOC193393) | FAOLEX specific legal record |
| **HR_12** | Harness supply chain and actions across sectors to ensure coherence with health | Regulation on the production of flour and bakery products. | 2020 | Other(ordinance) | This Ordinance sets flour yield minimums, usage limits for bakery products, and requirements for traceability, record-keeping, and reporting to the Ministry of Agriculture. | To regulate flour yields, usage, and ensure proper documentation and traceability in the milling and bakery sectors. | Bakery products | flour |  | [FAOLEX record: Regulation on the production of flour and bakery products](https://www.fao.org/faolex/results/details/en/c/LEX-FAOC192598) | FAOLEX specific legal record |
| **HR_13** | Nutrition label standards and regulations on the use of claims and implied claims on food | Regulation on marketing standards for eggs. | 2021 | Other (regulation) | The national regulation establishes standards for the production, labeling, packaging, import, and export of eggs. | The regulation ensures that eggs produced and imported meet specified quality and safety criteria before they are placed on the market for consumption or processing. | Eggs | Not specified |  | [FAOLEX record: Regulation on marketing standards for hatching eggs and offspring of domestic poultry](https://www.fao.org/faolex/results/details/en/c/LEX-FAOC203472) | FAOLEX specific legal record |
| **HR_14** | Harness supply chain and actions across sectors to ensure coherence with health | Regulation on the conditions, criteria and manner of granting aid under measure IV.4. "Processing of fishery and aquaculture products". | 2021 | Other (regulation) | This Regulation support the measure IV.4 "Processing of fishery and aquaculture products". The support focuses on investments in processing fishery and aquaculture products. | It sets conditions, criteria and manner of granting aid under measure IV.4. "Processing of fishery and aquaculture products". | fishery and aquaculture products | Not specified |  | [FAOLEX record: Regulation on granting aid under measure IV.4, “Processing of fishery and aquaculture products”](https://www.fao.org/faolex/results/details/en/c/LEX-FAOC200661) | FAOLEX specific legal record |
| **HR_15** | Harness supply chain and actions across sectors to ensure coherence with health | Regulation on the mandatory submission of data in the milk and milk products sector. | 2021 | Other (regulation) | The regulation prescribes mandatory reporting of raw cow, sheep and goat milk production quantities in the dairy sector. | To ensure compliance with EU regulations, and to provide accurate data on milk production for market and policy analysis. | Raw cow, sheep and goat milk | Not specified |  | [FAOLEX record: Regulation on the mandatory submission of data in the milk and milk products sector](https://www.fao.org/faolex/results/details/en/c/LEX-FAOC206792) | FAOLEX specific legal record |
| **HR_16** | Harness supply chain and actions across sectors to ensure coherence with health | Regulation on cereals and cereal products. | 2022 | Other (regulation) | The regulation prescribes general quality requirements for cereals intended for the final consumer for immediate consumption as well as cereal products that are placed on the market. | To set food security and quality standards for cereals. | Cereal products | Cereal |  | [FAOLEX record: Regulation on cereals and cereal products](https://www.fao.org/faolex/results/details/en/c/LEX-FAOC212462) | FAOLEX specific legal record |
| **HR_17** | Set incentives and rules to create a healthy retail and food service environment | Regulation on market standards for poultry meat. | 2022 | Other (regulation) | The regulation establishes comprehensive standards and procedures for ensuring the quality and safety of poultry meat. | To implement and enforce Regulation (EC) No. 543/2008 within Croatia, which sets detailed rules for the application of market standards for poultry meat across the European Union. | Poultry meat | Not specified |  | [FAOLEX record: Regulation on market standards for poultry meat](https://www.fao.org/faolex/results/details/en/c/LEX-FAOC210837) | FAOLEX specific legal record |
| **HR_18** | Harness supply chain and actions across sectors to ensure coherence with health | Law on food hygiene and microbiological criteria for food | 2022 | Law | This text regulates different food hygiene and microbiological criteria related topics and issues, such as: direct placing of food on the market (internal and international trade also), good practices, labelling requirements and special hygiene rules for food of animal origin. | To regulate different food hygiene and microbiological criteria related topics and issues. | All food products | Not specified |  | [FAOLEX record: Law on food hygiene and microbiological criteria for food](https://www.fao.org/faolex/results/details/en/c/LEX-FAOC211321) | FAOLEX specific legal record |
| **HR_19** | Harness supply chain and actions across sectors to ensure coherence with health | Law on food | 2023 | Law | This Law establishes the competent authorities and the tasks of the competent authorities, the obligations of subjects in the food and animal feed business, official controls, and prescribes administrative measures and misdemeanor provisions. | To promote food safety and quality. | All food products | Not specified |  | [FAOLEX record: Law on food](https://www.fao.org/faolex/results/details/en/c/LEX-FAOC214447) | FAOLEX specific legal record |
| **CY_1** | Harness supply chain and actions across sectors to ensure coherence with health | Infant Formula and Follow-on Formula Regulations | 2001 | Policy | This policy outlines the requirements for formulae and follow-on formulae intended for infants and young children. | To establish comprehensive standards for the production and marketing of infant and follow-on formulae. These standards are intended to ensure the safety, nutritional adequacy, and proper labeling of such products. | Infant food products | Not specified | X | [FAOLEX record: Infant Formula and Follow-on Formula Regulations, 2001](https://www.fao.org/faolex/results/details/en/c/LEX-FAOC085180) | FAOLEX specific legal record |
| **CY_2** | Nutrition label standards and regulations on the use of claims and implied claims on food | The Poultry Meat Marketing Specifications Law, no. 110 of 2020. | 2020 | Law | Matters covered by this Law include: (i) classification and labelling of poultry meat and exceptions; (ii) prohibition from receiving and possessing poultry meat not classified and marked, for the purpose of sale; (iii) powers of the competent Authority. | This legislation aims to regulate the labelling and safety of food products. | Poultry meat | Not specified |  | [FAOLEX record: The Poultry Meat Marketing Specifications Law, no. 110 of 2020](https://www.fao.org/faolex/results/details/en/c/LEX-FAOC211491) | FAOLEX specific legal record |
| **CY_3** | Harness supply chain and actions across sectors to ensure coherence with health | Law no. 39 of 2021 on the Preservation of Stocks of Raw Materials for Animal Feed and Grain for Human Use. | 2021 | Law | This law regulates the management and control of feed raw material and grain stocks for animal and human consumption. It covers areas like stock responsibilities, inventory management, stock replenishment, and financial resources. | The aim of this legislation is to promote food security along the supply chain. | Animal and human consumption products. | Feed raw material and grain stocks |  | [FAOLEX record: Law no. 39 of 2021 on the Preservation of Stocks of Raw Materials for Animal Feed and Grain for Human Use](https://www.fao.org/faolex/results/details/en/c/LEX-FAOC211511) | FAOLEX specific legal record |
| **CZ_1** | Harness supply chain and actions across sectors to ensure coherence with health | Act on foodstuffs and tobacco products | 1997 | Law | This Act lays down, in accordance with the legislation of the European Community, obligations of entrepreneurs dealing with production of foodstuffs and tobacco products and marketing of them and establishes public inspection controlling obligations specified in this Act. | This legislation aims to ensure that Czech food laws are in full compliance with EU standards, enhancing food safety, consumer protection, and regulatory oversight. | All food products | Not specified |  | [FAOLEX record: Act on foodstuffs and tobacco products](https://www.fao.org/faolex/results/details/en/c/LEX-FAOC094484) | FAOLEX specific legal record |
| **CZ_2** | Offer healthy food and set standards in public institutions and other specific settings | Decree No. 107/2005 on school meals | 2005 | Other (decree) | The decree regulates catering requirements for children, students, and others in full-day or boarding services. | To ensure the overall well-being of students in the educational system. | School food products | Not specified | X | [FAOLEX record: Decree no. 107/2005 on school meals](https://www.fao.org/faolex/results/details/en/c/LEX-FAOC174750) | FAOLEX specific legal record |
| **CZ_3** | Nutrition label standards and regulations on the use of claims and implied claims on food | Decree no. 366/2005 on requirements applicable to certain frozen foods. | 2005 | Other (decree) | The decree establishes requirements for certain frozen foods, including labeling, minimum technological requirements, storage, handling, transportation, and sampling. | To set quality and safety standards for the management of frozen foods, ensuring they are properly labeled and handled throughout all stages of the food chain. | Frozen foods | Not specified |  | [FAOLEX record: Decree no. 366/2005 on requirements applicable to certain frozen foods](https://www.fao.org/faolex/results/details/en/c/LEX-FAOC222954) | FAOLEX specific legal record |
| **CZ_4** | Harness supply chain and actions across sectors to ensure coherence with health | Decree on veterinary and sanitary requirements for animal products not covered by directly applicable regulations of the European Community | 2007 | Other (decree) | The decree outlines the veterinary and hygiene requirements for animal products that are not directly regulated by European Community regulations. | To ensure food safety and public health by establishing comprehensive standards and procedures for the handling, processing, and sale of these products. | Animal products | Not specified |  | [FAOLEX record: Decree on veterinary and sanitary requirements for animal products not covered by directly applicable EU regulations](https://www.fao.org/faolex/results/details/en/c/LEX-FAOC094445) | FAOLEX specific legal record |
| **CZ_5** | Offer healthy food and set standards in public institutions and other specific settings | HEALTH 2020 - National Strategy for Health Protection and Promotion and Disease Prevention | 2014 | Policy | This policy promotes healthier food environments through reformulation of products and improved school nutrition programs. It also focuses on increasing nutritional literacy, providing dietary advice, and enhancing healthcare capacity to address malnutrition. Monitoring, evaluation, and research on nutritional status are key components. | To improve public health by promoting proper nutrition and a healthy lifestyle through targeted interventions. | All food products | Nutrients | X | [Ministry of Health PDF: Health 2020 - National Strategy for Health Protection and Promotion and Disease Prevention](https://mzd.gov.cz/wp-content/uploads/wepub/8690/21944/Health%202020%20%E2%80%93%20National%20Strategy%20for%20Health%20Protection%20and%20Promotion%20and%20Disease%20Prevention.pdf) | Official government policy PDF |
| **CZ_6** | Restrict food advertising and other forms of commercial promotion | Decree 282/2016 on requirements for food that may be advertised, offered or sold in schools | 2016 | Other (decree) | The policy sets school food standards, banning sweeteners, caffeine, trans fats, and energy drinks, while permitting only low-sugar, low-fat items like fruits, vegetables, and select juices. | To establish nutritional standards for food sold in schools. | School food products | Sweeteners, caffeine, trans fats and sugar | X | [FAOLEX record: Decree on food requirements for which advertising is allowed and which can be offered for sale at schools](https://www.fao.org/faolex/results/details/en/c/LEX-FAOC174736) | FAOLEX specific legal record |
| **CZ_7** | Harness supply chain and actions across sectors to ensure coherence with health | Decree on requirements for milled cereal products, pasta, bakery products, confectionery products and doughs. | 2020 | Other (decree) | This decree regulates: a) information methods and classifications for cereal products, b) product types, c) quality standards and weight deviations, d) storage, handling, and technological requirements. | To set rules for products made from ground cereals, pastries and certified products. | Cereal products | Cereals |  | [FAOLEX record: Decree on requirements for milled cereal products, pasta, bakery products, confectionery products and doughs](https://www.fao.org/faolex/results/details/en/c/LEX-FAOC200399) | FAOLEX specific legal record |
| **CZ_8** | Harness supply chain and actions across sectors to ensure coherence with health | Decree on requirements for preserved fruit and preserved vegetables, nuts, mushrooms, potatoes and their products and bananas. | 2021 | Other (decree) | The decree specifies safety requirements to prevent contamination, spoilage, or other hazards during production, storage, and distribution. | To ensure that consumers receive safe, high-quality preserved foods that meet established standards of hygiene, safety, and nutritional value. | All food products | Not specified |  | [FAOLEX record: Decree on requirements for preserved fruit and vegetables, nuts, mushrooms, potatoes and bananas](https://www.fao.org/faolex/results/details/en/c/LEX-FAOC209676) | FAOLEX specific legal record |
| **CZ_9** | Harness supply chain and actions across sectors to ensure coherence with health | Decree on the list of foodstuffs according to § 9b par. 1c of the Food and Tobacco Products Act. | 2021 | Other (decree) | Specifies and categorizes food products according to criteria set forth in the Food and Tobacco Products Act. | To provide clear definitions and guidelines regarding the categorization and regulation of foodstuffs. | All food products | Not specified |  | [FAOLEX record: Decree on the list of foodstuffs according to § 9b par. 1c of the Food and Tobacco Products Act](https://www.fao.org/faolex/results/details/en/c/LEX-FAOC209671) | FAOLEX specific legal record |
| **CZ_10** | Harness supply chain and actions across sectors to ensure coherence with health | Decree on food requirements | 2023 | Other (decree) | This policy sets technological, marketing, and food safety standards for meals, including temperature limits for oils, meal storage, and microbiological safety. | The regulation aims to ensure food safety by setting standards to prevent microbiological and chemical risks from improper handling and storage. | All food products | Not specified |  | [FAOLEX record: Decree on food requirements](https://www.fao.org/faolex/results/details/en/c/LEX-FAOC220443) | FAOLEX specific legal record |
| **DK_1** | Set incentives and rules to create a healthy retail and food service environment | Food Act (No. 46 of 2017) | 1998 | Law | This Act serves as the legal foundation for ensuring that all aspects of food production and distribution in Denmark adhere to high standards of safety, quality, and transparency, thereby protecting consumers and maintaining fair practices within the food industry. | To promote healthy eating habits in Denmark. | All food products | Not specified |  | [FAOLEX record: Food Act (No. 46 of 2017)](https://www.fao.org/faolex/results/details/en/c/LEX-FAOC133881) | FAOLEX specific legal record |
| **DK_2** | Improve the nutritional quality of the food supply | Trans Fatty Acids regulation | 2004 | Other (regulation) | This regulation sets a 2% iTFA limit for oils and fats in both domestic and imported foods/effectively banning TFA in 2003. A transitional 5% iTFA limit was instated for the remainder of 2003, and the 2% limit came into full effect on January 1, 2004. | To reduce levels of artificial TFAs in food. | Domestic and imported food products | Trans Fatty Acids |  | [FAOLEX/ECOLEX record: Order No. 1427 on content of trans fatty acids in oils and fats](https://www.fao.org/faolex/results/details/en/c/LEX-FAOC150333) | FAOLEX specific legal record |
| **DK_3** | Harness supply chain and actions across sectors to ensure coherence with health | Nordic plan of action on better health and quality of life through diet and physical activity | 2006 | Policy | This plan represents a collective Nordic contribution to the global dialogue on preventing obesity and encouraging healthy living by promoting better dietary practices and physical activity, highlighting the role of Nordic cooperation in enhancing the effectiveness of health initiatives. | To enhance health and quality of life across the Nordic countries through improved diet and physical activity, aiming to leverage regional cooperation to create synergistic benefits in promoting healthy lifestyles. | All food products | Not specified |  | [Nordic Council of Ministers publication: A better life through diet and physical activity](https://www.norden.org/en/publication/better-life-through-diet-and-physical-activity) | Official Nordic policy publication |
| **DK_4** | Restrict food advertising and other forms of commercial promotion | Code of responsible food marketing communication to children | 2008 | Policy | The policy seeks to ensure that such marketing practices comply with Danish laws and ethical standards, especially concerning foods high in sugar, fat, or salt, in order to protect children from potentially harmful advertising. | To regulate advertising and sponsorship practices related to food products targeting children under 13 years of age. | Children food products | Sugar, fat and salt | X | [FoodDrinkEurope: Danish Forum of Responsible Food Marketing Communication](https://www.fooddrinkeurope.eu/industry-action/danish-forum-of-responsible-food-marketing-communication/) | Policy-specific stakeholder/industry source |
| **DK_5** | Nutrition label standards and regulations on the use of claims and implied claims on food | The Whole Grain Logo | 2009 | Policy | The Whole Grain Logo may be applied to products containing significant amounts of whole grains, including products such as rye bread, wheat bread, flour, cereals, crispbread, and breakfast cereals. To qualify for the Whole Grain Logo, products must also meet the Keyhole label's nutritional criteria. | The aim of this policy is to encourage healthier eating habits by identifying products that are high in whole grains and adhere to standards for fat, sugar, salt, and dietary fiber content. | Bread, wheat bread, flour, cereals, crispbread, and breakfast cereals. | Whole grains |  | [Whole Grain Partnership: The whole grain logo](https://fuldkorn.dk/en/about-us/the-whole-grain-logo/) | Official programme/partnership page |
| **DK_6** | Nutrition label standards and regulations on the use of claims and implied claims on food | Keyhole labeling policy | 2009 | Policy | The Keyhole labeling policy belongs to a Joint Nordic Initiative driven by the desire to promote healthier food choice and provide consumers with more information about nutritional content of the products. | To allow consumers to find and compare at a glance the nutritional quality of pre-packaged foodstuffs. | All food products | Nutrients |  | [FAOLEX record: Order No. 238 on the use of the Keyhole symbol on food products](https://www.fao.org/faolex/results/details/en/c/LEX-FAOC195933) | FAOLEX specific legal record |
| **DK_7** | Restrict food advertising and other forms of commercial promotion | Regulation on Infant Formula and Follow-on Formulas for Infants and Young Children | 2012 | Other (regulation) | The policy regulates advertising and product presentation, covering product form, packaging, placement, and display environment. | To regulate the marketing and advertising practices for infant formula. | Infant food products | Not specified | X | [FAOLEX record: Order No. 1198 on infant formulas for infants](https://www.fao.org/faolex/results/details/en/c/LEX-FAOC127660) | FAOLEX specific legal record |
| **DK_8** | Use economic tools to address food affordability and purchase incentives | Act on Taxation of Chocolate and Confectionary Products, etc. (Chocolate Tax Act) - No. 1010 of 2018 | 2018 | Law | The present Act establishes a series of taxes on chocolate products and other confectionery products. It contains the following Chapters: 1) Tax on chocolate and confectionary products; 2) Tax on raw materials; 3) Coverage tax; 4) General provisions. | To regulate and tax certain confectionery and sugary products to ensure compliance and revenue generation for the state treasury. | Chocolate and other confectionery products | Sugar and sweeteners |  | [FAOLEX record: Act on taxation of chocolate and confectionary products, etc. (Chocolate Tax Act)(No. 1010 of 2018)](https://www.fao.org/faolex/results/details/en/c/LEX-FAOC201640) | FAOLEX specific legal record |
| **DK_9** | Harness supply chain and actions across sectors to ensure coherence with health | Order No. 1349 on on the import of food with special restrictions and penalties for the infringement of EU legislation. | 2019 | Law | The law details restrictions on the importation of specific foodstuffs into Denmark and transit through Denmark to other trading countries. | The aim of the law is to set requirements for documentation, sampling, and testing of imported foodstuffs, particularly focusing on animal products and potential contaminants such as histamine in fish products from specific regions. | Imported animal-based products | Not specified |  | [FAOLEX record: Order No. 1349 on imports of food with special restrictions and penalties](https://www.fao.org/faolex/results/details/en/c/LEX-FAOC195815) | FAOLEX specific legal record |
| **DK_10** | Harness supply chain and actions across sectors to ensure coherence with health | Order No. 406 on veterinary control of imports of food of animal-origin and penalties for the violation of related EU legislation. | 2019 | Other (ministerial order) | This Executive Order encompasses all forms of importation into Denmark of animal-based foods listed in Annex 1, Part I. | To set veterinary control for the Import of Animal-Based Foods and Penalties for Violation of Related EU Regulations. | Imported animal-based products | Not specified |  | [FAOLEX record: Order No. 406 on veterinary control of imports of food of animal-origin](https://www.fao.org/faolex/results/details/en/c/LEX-FAOC195848) | FAOLEX specific legal record |
| **DK_11** | Harness supply chain and actions across sectors to ensure coherence with health | Order No. 726 on the addition to food of certain substances other than vitamins and minerals. | 2020 | Other (ministerial order) | This order specifies the scope and definitions related to the addition of substances to foods, including dietary supplements, for nutritional or physiological purposes. | It aims to regulate the addition of certain other substances than vitamins and minerals to foods. | Dietary supplements | Not specified |  | [FAOLEX record: Order No. 726 on the addition to food of certain substances other than vitamins and minerals](https://www.fao.org/faolex/results/details/en/c/LEX-FAOC195934) | FAOLEX specific legal record |
| **DK_12** | Harness supply chain and actions across sectors to ensure coherence with health | Order No. 1299 on dairy products, etc. | 2022 | Other (ministerial order) | This Order lays down rules on the production and composition, as well as on the labelling, of dairy products. | To regulate the production, composition and labelling of dairy products. | Dairy products. | Not specified |  | [FAOLEX record: Order No. 1299 on dairy products, etc.](https://www.fao.org/faolex/results/details/en/c/LEX-FAOC214945) | FAOLEX specific legal record |
| **DK_13** | Harness supply chain and actions across sectors to ensure coherence with health | Regulation No. 1163 on bread wheat. | 2022 | Other (regulation) | The regulation lays down the criteria that wheat varieties shall meet in terms of nitrogen standard during cultivation, in accordance with the applicable Order on agricultural use of fertilizers for the planning period. | The objective of the law is to ensure food safety and protect consumer health by ensuring that wheat varieties placed on the market are safe, of high quality, and properly labeled. | Wheat-based products | Wheat |  | [FAOLEX/ECOLEX record: Regulation No. 1163 on bread wheat](https://www.fao.org/faolex/results/details/en/c/LEX-FAOC217867) | FAOLEX specific legal record |
| **EE_1** | Set incentives and rules to create a healthy retail and food service environment | Food Act | 1999 | Law | This act lays down the rules for the management of food and raw materials, self-control of operators and state supervision, ensuring food safety and compliance with requirements. | The aim of this law is to regulate the handling of food and raw materials, ensure food safety and compliance with relevant requirements. | All food products | Not specified |  | [Riigi Teataja: Food Act consolidated text](https://www.riigiteataja.ee/en/eli/518102024018/consolide) | Official national legal database |
| **EE_2** | Set incentives and rules to create a healthy retail and food service environment | Food Intended for Particular Nutritional Uses (Regulation No. 436) | 2000 | Other (regulation) | The regulation provides guidelines for manufacturing, handling, and labeling of specialized foodstuffs like Infant and Follow-On Formulae. | To establish standards for foodstuffs catering to specific nutritional needs, ensuring safety, quality, and accurate labeling. | Infant food products | Not specified | X | [FAOLEX record: Governmental Decree No.436 of 1999 on foodstuffs intended for particular nutritional uses](https://www.fao.org/faolex/results/details/en/c/LEX-FAOC037809) | FAOLEX specific legal record |
| **EE_3** | Nutrition label standards and regulations on the use of claims and implied claims on food | Regulation No. 324 on food labelling | 2004 | Other (regulation) | The policy sets the maximum allowable sodium chloride content, by mass percentage, for various food products. | To limit the maximum sodium chloride content in food products. | All food products | Sodium chloride |  | [European Commission PDF: National Salt Initiatives - Estonia reference to Government Regulation No. 324](https://ec.europa.eu/health/ph_determinants/life_style/nutrition/documents/national_salt_en.pdf) | EU policy documentation / secondary source |
| **EE_4** | Offer healthy food and set standards in public institutions and other specific settings | Regulation on health protection requirements for catering facilities in pre-school institutions, schools | 2008 | Other (regulation) | The policy mandates the creation of a 10-day menu for child care institutions, including weekends for 24-hour facilities, according to the daily energy and nutrient needs of different age groups, with specific limits on protein, fat, and sugar intake. | To ensure that children in care institutions receive well-balanced, nutritious meals that meet their specific dietary needs, promoting overall health and development while minimizing exposure to unhealthy foods. | Children food products | Nutrients | X | [FAOLEX record: Ministerial Regulation no. 8 of 2008 concerning health protection requirements for catering in preschools and schools](https://www.fao.org/faolex/results/details/en/c/LEX-FAOC238309) | FAOLEX specific legal record |
| **EE_5** | Set incentives and rules to create a healthy retail and food service environment | Veterinary Act (2021). | 2021 | Law | This Act, among its main functions, regulates the handling of animal-based food products. | To promote food safety. | Animal-based food products. | Not specified |  | [ECOLEX record: Veterinary Act (2021)](https://www.ecolex.org/details/legislation/veterinary-act-2021-lex-faoc211875/) | ECOLEX/FAOLEX specific legal record |
| **FI_1** | Restrict food advertising and other forms of commercial promotion | Finnish Consumer Protection Act (38/1978) | 1978 | Law | The law governs the offering, selling, and marketing of goods and services to consumers by prohibiting deceptive, unfair, and discriminatory practices, requiring transparency and accuracy in information, ensuring marketing practices align with societal values. | This Act applies to the offering, selling and other marketing of consumer goods or services by traders to consumers. | All food products | Not specified |  | [Finlex translation: Consumer Protection Act 38/1978](https://www.finlex.fi/en/legislation/translations/1978/eng/38); [FAOLEX PDF: Consumer Protection Act](https://faolex.fao.org/docs/pdf/fin36898E.pdf) | Official national legal database; FAOLEX legal text |
| **FI_2** | Nutrition label standards and regulations on the use of claims and implied claims on food | Compulsory use of warning labels on high-salt foods | 1991 | Policy | This policy ensures that certain food products are properly labeled as "high in salt" and "low in salt" if their sodium chloride content exceeds, and not, certain thresholds. | To ensure that consumers make informed choices about certain food products in Finland based on salt content. | All food products | Salt |  | [Finlex: Ministry of Trade and Industry Decision on food package labelling 795/1991](https://www.finlex.fi/fi/lainsaadanto/saadoskokoelma/1991/795); [Finnish Food Authority: mandatory food information and high-salt labelling](https://www.ruokavirasto.fi/elintarvikkeet/elintarvikeala/pakkausmerkinnat-ja-markkinointi/pakolliset-elintarviketiedot/) | Official national legal database; official food authority guidance |
| **FI_3** | Nutrition label standards and regulations on the use of claims and implied claims on food | National legislation on compulsory ‘warning labelling’ of high salt foods | 1993 | Law | In accordance to this legislation, foods that are high in salt are required to carry a "high salt content" warning. A "high salt content" must be labelled, if the salt content is more than 1.3% in bread, 1.8% in sausages, 1.4% in cheese, 2.0% in butter, and 1.7% in breakfast cereals or crisp bread. | This legislation aims to inform consumers about the salt content in food products (salt labelling), helping them make healthier dietary choices and reduce sodium intake. | All food products | Salt |  | [Finlex: Decree 1010/2014 on labelling certain foods as high in salt](https://www.finlex.fi/fi/lainsaadanto/2014/1010); [World Action on Salt: Finland salt-labelling legislation summary](https://www.worldactiononsalt.com/worldaction/europe/finland/) | Official national legal database; expert policy database/source |
| **FI_4** | Restrict food advertising and other forms of commercial promotion | Decree 807/1994 - Policy on marketing material on feeding infants and small children | 1994 | Other (decree) | This decree outlines strict guidelines for the creation and distribution of informational and advisory materials related to infant nutrition. | To protect parents and caregivers from misleading or biased information related to infant and child nutrition, ensuring that such materials promote breastfeeding as the superior method of feeding. | Infant food products | Not specified | X | [Finlex: Ministry of Social Affairs and Health Decision 807/1994](https://www.finlex.fi/fi/lainsaadanto/1994/807); [Finlex PDF translation of replacing Decree 267/2010, citing repeal of 807/1994](https://www.finlex.fi/api/media/statute-foreign-language-translation/688137/mainPdf/main.pdf?timestamp=2010-04-15T21%3A00%3A00.000Z) | Official national legal database; official legal translation/PDF |
| **FI_5** | Nutrition label standards and regulations on the use of claims and implied claims on food | Action Plan for Promoting Finnish Heart symbol | 2000 | Policy | It represents the only symbol in Finland giving information about the nutritional quality of food products. | To inform consumers at a glance that a food product with this symbol is a nutritionally better choice among its product group in terms of fat and salt. | All food products | Nutrients |  | [Official Finnish Heart Symbol criteria](https://www.sydanmerkki.fi/en/criteria); [European Commission document: Finnish Heart Association Heart Symbol system](https://food.ec.europa.eu/document/download/e39ed09f-b1da-4e7a-9322-42dba833b8af_en) | Official programme website; European Commission-hosted policy document |
| **FI_6** | Nutrition label standards and regulations on the use of claims and implied claims on food | Decree 1224/2007 of the Ministry of Trade and Industry on amending the regulation of the Ministry of Trade and Industry on food packaging labels] | 2008 | Other (decree) | This policy mandates labeling the salt content in certain foods, by specifying the percentage of sodium chloride by weight on packaging. | To ensure transparency about salt content in food products. | All food products | Salt |  | [Finlex: Decree 1224/2007 amending food package labelling regulation](https://www.finlex.fi/fi/lainsaadanto/saadoskokoelma/2007/1224); [Finlex: underlying food package labelling regulation 1084/2004](https://www.finlex.fi/fi/lainsaadanto/2004/1084) | Official national legal database |
| **FI_7** | Improve the nutritional quality of the food supply | National legislation on products entitled to EU subsidies (School Milk Scheme). | 2009 | Law | This measure promotes the consumption of milk-based products by young people, and to have an educational influence on dietary habits. | To guarantee the sale of reduced-rate milk products to schoolchildren. | Milk products | Not specified | X | [Finlex translation: School Milk Subsidy Decree 595/2001](https://www.finlex.fi/en/legislation/translations/2001/eng/595); [EU Salt Reduction Framework: Finland school milk upper salt limit](https://www.aesan.gob.es/AECOSAN/docs/documentos/nutricion/observatorio/encuesta_estados_miembros_sal.pdf) | Official national legal database; EU/Member State policy report |
| **FI_8** | Harness supply chain and actions across sectors to ensure coherence with health | Government Decree on food hygiene in notified food premises (No. 1367 of 2011). | 2011 | Other (decree) | The Decree addresses self-monitoring, temperature control for perishable foods, conditions for food sales, personal hygiene, animal access, and the transport of raw and non-heat-treated milk. | To ensure food hygiene in notified food premises. | Perishable foods, raw and non-heat-treated milk. | Not specified |  | [Finlex: Decree 1367/2011 on food hygiene in notified food premises](https://www.finlex.fi/fi/laki/alkup/2011/20111367); [FAOLEX listing: Finland food hygiene in notified food premises](https://www.fao.org/faolex/country-profiles/general-profile/see-more/fr/?area=Food+and+nutrition&countryname=Finlande&iso3=FIN&link=aHR0cHM6Ly9mYW9sZXguZmFvLm9yZy9jZ2ktYmluL3htbC5leGU%2FZGF0YWJhc2U9ZmFvbGV4JmFtcDtzZWFyY2hfdHlwZT1xdWVyeSZhbXA7dGFibGU9YWxsJmFtcDtxdWVyeT1BUkVBOkZEIEFORCBDQzpGSU4gQU5EIFQ6QUxMIEFORCBSRVBFQUxFRDpOIEFORCBTVVBFUlM6TiBOT1QgUk86WSBBTkQgWjooTCBSIE0pIE5PVCBaOlAmYW1wO3NvcnRfbmFtZT1Ac3ByZkZEJmFtcDtsYW5nPXhtbGYmYW1wO2Zvcm1hdF9uYW1lPUBYU0hPUlQmYW1wO3BhZ2VfaGVhZGVyPUVYTUxIJmFtcDtwYWdlX2Zvb3Rlcj1FWE1MRg%3D%3D) | Official national legal database; FAOLEX legal database listing |
| **FI_9** | Use economic tools to address food affordability and purchase incentives | Excise duty on sweets and ice-cream | 2011 | Policy | Finland had a sweets tax in 1999–2000, the tax rate amounted to 0.75€/kg for solid tax-liable products. The tax was reintroduced in January 2011 with a new tax rate amounted to 0.95 Euro per kg by weight for confectionery and ice cream. | The aim was to reduce the consumption of candy and ice cream. | Confectionery and ice cream. | Not specified |  | [Finlex: Act 1127/2010 on excise duty on sweets, ice cream and soft drinks](https://www.finlex.fi/fi/lainsaadanto/saadoskokoelma/2010/1127); [European Commission Taxes in Europe: excise duty on sweets, ice-cream and soft drinks](https://ec.europa.eu/taxation_customs/tedb/legacy/taxDetail.html?id=2001%2F1424159141&taxType=Other+indirect+tax) | Official national legal database; European Commission tax database |
| **FI_10** | Nutrition label standards and regulations on the use of claims and implied claims on food | Decree 1010/2014 of the Ministry of Agriculture and Forestry declaring certain foods to be high in salt] | 2016 | Other (decree) | The regulation requires labeling certain food products as "high in salt" when they exceed specified thresholds, such as 1.4% for cheese, 2.0% for sausages and fish products. | To inform consumers about high salt content and promote healthier dietary choices. | All food products | Salt |  | [Finlex: Decree 1010/2014 on labelling certain foods as high in salt](https://www.finlex.fi/fi/lainsaadanto/2014/1010); [Edilex PDF: official statute publication 1010/2014](https://www.edilex.fi/saadoskokoelma/siirry/20141010) | Official national legal database; official statute PDF |
| **FI_11** | Offer healthy food and set standards in public institutions and other specific settings | Government Decree on a subsidy for the distribution of dairy products, fruits and vegetables in school for the academic year 2020-2021(No. 421 of 2020). | 2020 | Other (decree) | This decree contains provisions on the aid for distribution in schools as referred to in the Act on the Market Organization for Agricultural Products (999/2012). | To promote the distribution of Milk Products, Fruits, and Vegetables in Schools. | School food products (Milk Products, Fruits, and Vegetables) | Not specified | X | [ECOLEX/FAOLEX: Government Decree No. 421 of 2020](https://www.ecolex.org/fr/details/legislation/government-decree-on-a-subsidy-scheme-for-the-distribution-of-dairy-products-fruits-and-vegetables-in-school-for-the-academic-year-2020-2021no-421-of-2020-lex-faoc195768/) | ECOLEX/FAOLEX legal record |
| **FI_12** | Harness supply chain and actions across sectors to ensure coherence with health | Decree on food supervision (No. 315 of 2021) | 2021 | Other (decree) | This decree ensures the safety of germinated products and meat production, allowing slaughterhouse staff to assist with inspections under certain conditions. | To enhance food safety, maintain hygiene standards, and ensure proper inspection protocols in primary production and meat processing. | Germinated and meat products | Not specified |  | [Finlex: Decree 315/2021 on food supervision](https://www.finlex.fi/en/legislation/2021/315); [FAOLEX listing: Finland Decree No. 315 of 2021 on food supervision](https://www.fao.org/faolex/highlights/seemore/en/?link=aHR0cDovL2Zhb2xleC5mYW8ub3JnL2NnaS1iaW4veG1sLmV4ZT9kYXRhYmFzZT1mYW9sZXgmYW1wO3NlYXJjaF90eXBlPXF1ZXJ5JmFtcDt0YWJsZT1hbGwmYW1wO3F1ZXJ5PVQ6QUxMIEFORCAoQ0s6KDAwMSAwMDIgODA2KSBPUiBDTUFJTjpbMDAwIFRPIDk5OV0pIEFORCBSRVBFQUxFRDpOIEFORCBBTUVORDpOIEFORCBZVEw6KDIwMjEgMjAyMCAyMDE5IDIwMTgpIE5PVCBSTzpZIE5PVCBaOlAgQU5EIEFSRUE6RkQmYW1wO2ZyZWVfdGV4dD0yMDIxMDQyNSZhbXA7c29ydF9uYW1lPUBzY2FsbCZhbXA7bGFuZz14bWxmJmFtcDtmb3JtYXRfbmFtZT1AWFNIT1JUJmFtcDtwYWdlX2hlYWRlcj1FWE1MSCZhbXA7cGFnZV9mb290ZXI9RVhNTEY%3D&subject=Food+and+nutrition) | Official national legal database; FAOLEX legal database listing |
| **FI_13** | Set incentives and rules to create a healthy retail and food service environment | Food Act (No. 297/2021). | 2021 | Law | It covers all stages of the production, processing, and distribution chain for food and food contact materials, including activities of food business operators and food inspectors. The Act promotes healthy food practices and requires accurate and sufficient information about food products. It excludes primary production for private household use and certain alcoholic beverages. | To protect consumer health and economic interests by ensuring the safety and quality of food products and food contact materials throughout the production, processing, and distribution chain. | All food products | Not specified |  | [Finlex translation: Food Act 297/2021](https://www.finlex.fi/en/legislation/translations/2021/eng/297); [Finlex PDF translation: Food Act 297/2021](https://www.finlex.fi/api/media/statute-foreign-language-translation/687572/mainPdf/main.pdf?timestamp=2021-04-08T21%3A00%3A00.000Z) | Official national legal database; official legal translation/PDF |
| **FR_1** | Harness supply chain and actions across sectors to ensure coherence with health | Décret n°71-644 du 30 juillet 1971 portant application de la loi modifiée du 1er août 1905 sur la répression des fraudes dans la vente des marchandises et des falsifications des denrées alimentaires et des produits agricoles, en ce qui concerne les produits utilisés en agriculture ou en élevage, pouvant être tolérés dans les denrées alimentaires et les boissons | 1971 | Other (decree) | This decree addresses residues from products used in agriculture or livestock farming that may be tolerated in foodstuffs and beverages, prohibiting those products that pose a danger to human health. | To prevent fraud in the sale of foodstuffs and agricultural products. | Agriculture or livestock farming products | Not specified |  | [Légifrance: Décret n°71-644 du 30 juillet 1971](https://www.legifrance.gouv.fr/loda/id/JORFTEXT000000879030) | Official national legal database |
| **FR_2** | Harness supply chain and actions across sectors to ensure coherence with health | Arrêté du 9 mars 1981 relatif au retrait de la consommation humaine des produits de la mer et d'eau douce conservés ou préparés à l'aide de substances non autorisées par la réglementation en vigueur | 1981 | Other (decree) | The decree prohibits the human consumption of seafood and freshwater products that have been preserved or prepared with substances not authorized by current regulations. | This decree aims to protect public health by preventing the marketing of aquatic products treated with non-approved preservatives or additives, thereby guaranteeing consumer safety. | Seafood and freshwater products | Preservatives and additives |  | [Légifrance: Arrêté du 9 mars 1981](https://www.legifrance.gouv.fr/loda/id/JORFTEXT000000654297) | Official national legal database |
| **FR_3** | Improve the nutritional quality of the food supply | Arrêté du 23 juin 1993 relatif au sel alimentaire et aux substances d'apport nutritionnel pouvant être utilisées pour sa supplémentation | 1993 | Policy | The policy ensures controlled addition of iodine and fluoride to table salt to support public health, addressing iodine deficiency and dental health. | To regulate the iodization and fluoridation of table salt to improve public health. | Table salt | Iodine and fluoride |  | [Légifrance: Arrêté du 23 juin 1993](https://www.legifrance.gouv.fr/loda/id/JORFTEXT000000728938/) | Official national legal database |
| **FR_4** | Harness supply chain and actions across sectors to ensure coherence with health | Décret n°93-1074 du 13 septembre 1993 pris pour l'application de la loi du 1er août 1905 en ce qui concerne certaines catégories de pains | 1993 | Other (decree) | The decree sets labeling criteria for bread: only bread kneaded, shaped, and baked on-site can be called "home-made," while additive-free, unfreezed bread meeting specific standards may be labeled "French traditional bread." | To set clear labeling criteria for different bread types, ensuring transparency and quality. | Bread | Not specified |  | [Légifrance: Décret n°93-1074 du 13 septembre 1993](https://www.legifrance.gouv.fr/loda/id/JORFTEXT000000727617/) | Official national legal database |
| **FR_5** | Offer healthy food and set standards in public institutions and other specific settings | French National Nutrition and Health Program (PNNS) | 2001 | Policy | The policy aims to increase healthy nutrients while reducing fats, sugars, and alcohol to curb excessive caloric intake. | To improve population health through strategies to enhance diets. | All food products | Nutrients, fats, sugars, and alcohol |  | [Ministry of Health: PNNS 2001–2005 presentation PDF](https://sante.gouv.fr/IMG/pdf/presentation_generale_programme.pdf) | Official government policy document |
| **FR_6** | Offer healthy food and set standards in public institutions and other specific settings | Loi n° 2004-806 du 9 août 2004 relative à la politique de santé | 2004 | Law | This law bans the installation of vending machines in schools to improve children's dietary habits. | To mitigate childhood obesity and promote healthy eating habits among students. | School food products | Not specified | X | [Légifrance: Loi n°2004-806](https://www.legifrance.gouv.fr/loda/id/JORFTEXT000000787078); [Légifrance: Article 30 on school vending machines](https://www.legifrance.gouv.fr/loda/article_lc/LEGIARTI000006697648/2004-08-11) | Official national legal database |
| **FR_7** | Offer healthy food and set standards in public institutions and other specific settings | Second national nutrition and health programme 2006-2010 | 2006 | Policy | The policy aims to improve fruit and vegetable intake by 25% in around 45% of the population while increasing calcium intake and decreasing total fat intake to under 35% of daily energy. | To improve population health by setting nutritional goals to enhance diets and address health concerns. | All food products | Fruit, vegetable, calcium and fat |  | [Second PNNS 2006–2010, Actions and measures PDF](https://www.irbms.com/download/documents/programme-national-nutrition-sante-pnns-2006-2010.pdf) | Government programme document / archived PDF copy |
| **FR_8** | Harness supply chain and actions across sectors to ensure coherence with health | Arrêté du 30 juin 2008 relatif aux limites maximales applicables aux résidus de chlordécone que ne doivent pas dépasser certaines denrées alimentaires d'origine végétale et animale pour être reconnues propres à la consommation humaine. | 2008 | Other (decree) | This decree sets maximum residue limits (MRLs) for chlordécone in specific foodstuffs of plant and animal origin to ensure they are safe for consumption. | To enforce MRLs for chlordécone in foodstuffs, ensuring food safety and protecting public health from harmful residue levels. | Plant and animal origin food products | Chlordécone |  | [Légifrance: Arrêté du 30 juin 2008](https://www.legifrance.gouv.fr/loda/id/JORFTEXT000019117823) | Official national legal database |
| **FR_9** | Restrict food advertising and other forms of commercial promotion | Loi de modernisation de l'économie | 2008 | Law | This law establishes rules for food advertising, including requirements for mandatory health or nutrition messages in advertising campaigns. | This law aims to ensure that French consumers receive accurate and transparent information about health and nutrition through food advertising. | All food products | Nutrients |  | [Légifrance: Loi n°2008-776 de modernisation de l'économie](https://www.legifrance.gouv.fr/loda/id/JORFTEXT000019283050); [Légifrance: Code de la santé publique, Article L2133-1](https://www.legifrance.gouv.fr/codes/section_lc/LEGITEXT000006072665/LEGISCTA000006171131/2026-03-13); [Légifrance: Arrêté du 27 février 2007 on sanitary information in food/beverage advertising](https://www.legifrance.gouv.fr/loda/id/LEGITEXT000006055557) | Official national legal database |
| **FR_10** | Harness supply chain and actions across sectors to ensure coherence with health | Arrêté du 18 décembre 2009 relatif aux règles sanitaires applicables aux produits d'origine animale et aux denrées alimentaires en contenant | 2009 | Other (regulation) | This article establishes specific sanitary rules for the production, processing, and sale of products of animal origin. It sets hygiene standards for slaughterhouses, cutting plants, and the direct supply of small quantities of primary products to local markets, ensuring compliants with EU regulations. | To maintain food safety by setting additional hygiene requirements for animal products and related foodstuffs. | Animal origin products | Not specified |  | [Légifrance: Arrêté du 18 décembre 2009](https://www.legifrance.gouv.fr/loda/id/JORFTEXT000021533994) | Official national legal database |
| **FR_11** | Inform people about food and nutrition through public awareness | ARPP Advertising Code: Food behaviours recommendation | 2009 | Policy | This policy requires all food and beverage advertisements to promote healthy eating and physical activity, especially for young audiences. Ads must align with the National Nutrition and Health Program’s guidelines, avoiding the promotion of unhealthy behaviors, and should support public health recommendations across all media. | To prevent advertising from undermining efforts to improve dietary practices and food hygiene. | All food products | Not specified |  | [ARPP: Recommandation Comportements alimentaires, September 2009 PDF](https://www.arpp.org/wp-content/uploads/2009/10/Reco_Comp_Alimentaires.pdf) | Professional advertising self-regulatory code / policy document |
| **FR_12** | Offer healthy food and set standards in public institutions and other specific settings | Decree No. 2011-1227 of September 30, 2011, relating to the nutritional quality of meals served as part of school catering | 2011 | Other (decree) | The decree establishes nutritional quality requirements for canteen managers regarding meal diversity, portion sizes, and the provision of water, bread, salt, and sauces. | To ensure the nutritional quality of meals in canteens. | School food products | Nutrients | X | [Légifrance: Décret n°2011-1227](https://www.legifrance.gouv.fr/jorf/id/JORFTEXT000024614716); [Légifrance: Arrêté du 30 septembre 2011 on school meal nutritional quality](https://www.legifrance.gouv.fr/loda/id/JORFTEXT000024614763/) | Official national legal database |
| **FR_13** | Harness supply chain and actions across sectors to ensure coherence with health | Programme National Nutrition Santé (PNNS) 2011-15 | 2011 | Policy | The PNNS 2011-2015 was designed to create a comprehensive approach to improving the nutritional health of the French population, addressing both individual behaviors and broader social determinants of health. | To reduce the prevalence of obesity and overweight in the population, promote healthy eating practices, and encourage physical activity. | All food products | Not specified |  | [Ministry of Health: PNNS 2011–2015 PDF](https://sante.gouv.fr/IMG/pdf/PNNS_2011-2015.pdf) | Official government policy document |
| **FR_14** | Harness supply chain and actions across sectors to ensure coherence with health | Arrêté du 24 avril 2013 relatif à la lutte contre les infections à salmonelles considérées comme dangers sanitaires de première catégorie dans les troupeaux de poulets de chair et de dindes d'engraissement et fixant les modalités de déclaration des salmonelles considérées comme dangers sanitaires de deuxième catégorie dans ces troupeaux | 2013 | Other (ministerial order) | This order establishes a national program to control Salmonella infections in broiler chicken and fattening turkey flocks. It mandates systematic screening for Salmonella, decontamination of infected sites, and management of meat from infected flocks. | To control Salmonella infections in poultry and reduce the risk of contamination in the food supply. | Poultry meat | Salmonella |  | [Légifrance: Arrêté du 24 avril 2013](https://www.legifrance.gouv.fr/loda/id/JORFTEXT000027415222) | Official national legal database |
| **FR_15** | Nutrition label standards and regulations on the use of claims and implied claims on food | Nutri-Score Labeling Policy | 2017 | Policy | It represents a front-of-pack labelling system providing a synthetic information system based on colours and letters (from green/A to red/E), to allow consumers to see and compare at a glance the nutritional value of pre-packaged foods. | The main aim of the Nutri-Score is to guide consumers toward healthier food choices and promote better public health by providing simple, color-coded nutritional information. | Pre-packaged food products | Nutrients |  | [Légifrance: Arrêté du 31 octobre 2017 on the complementary nutrition declaration presentation](https://www.legifrance.gouv.fr/jorf/id/JORFTEXT000035944131) | Official national legal database |
| **FR_16** | Harness supply chain and actions across sectors to ensure coherence with health | LOI no 2018-938 du 30 octobre 2018 pour l’équilibre des relations commerciales dans le secteur agricole et alimentaire et une alimentation saine, durable et accessible à tous | 2018 | Law | This law, divided into 4 sections, regulates the relationship between the agricultural and the food sector. | The law aims to: improve the balance of commercial relations in the agricultural and food sector (Title I), and to provide healthy, sustainable, and accessible food, respectful of animal welfare (Title II). | Agricultural products | Not specified |  | [Légifrance: Loi n°2018-938 (EGAlim)](https://www.legifrance.gouv.fr/loda/id/JORFTEXT000037547946/) | Official national legal database |
| **FR_17** | Harness supply chain and actions across sectors to ensure coherence with health | Programme national de l'alimentation et de la nutrition (PNAN) | 2019 | Policy | This policy promotes healthy eating habits, encourages physical activity, and reduces sedentary behavior. It also aims to reduce health inequalities, aligning with the National Health Strategy 2018-2022 and the "Priority Prevention" Plan. Evaluations are based on public health nutrition goals set by the High Council for Public Health (HCSP). | To improve population health by focusing on nutrition and physical activity as key determinants. | All food products | Not specified |  | [Ministry of Agriculture: PNAN programme page](https://agriculture.gouv.fr/pnan-le-programme-national-de-lalimentation-et-de-la-nutrition); [Ministry of Agriculture: PNAN PDF](https://agriculture.gouv.fr/telecharger/103094?token=c23b9df7da43d8fcc7c22fde15f01658) | Official government policy page and PDF |
| **DE_1** | Harness supply chain and actions across sectors to ensure coherence with health | Act on the marketing of milk, milk products and fats | 1951 | Law | This legislation regulates the production, distribution, quality and marketing of milk and milk products, imposing specific obligations on producers, dairies and traders to ensure proper management of the sector and to protect the quality of products offered to consumers. | This legislation aims to ensure the quality and safety of milk and dairy products. | Milk and dairy products. | Not specified |  | [Gesetze im Internet: Milch- und Fettgesetz (MilchFettG)](https://www.gesetze-im-internet.de/milchfettg/BJNR001350951.html); [Direct PDF: MilchFettG](https://www.gesetze-im-internet.de/milchfettg/MilchFettG.pdf) | Official national legal database; direct statute PDF |
| **DE_2** | Harness supply chain and actions across sectors to ensure coherence with health | Cheese regulation | 1965 | Other (regulation) | This regulation sets standards for the production, marketing, and quality testing of cheese and cheese products. It includes rules on labeling, branding, production processes, and the use of rennet substitutes, with enforcement authority remaining at the federal state level. | To regulate the production, marketing, and quality control of cheese to ensure consistent standards. | Cheese and cheese products | Not specified |  | [Gesetze im Internet: Käseverordnung (KäseV)](https://www.gesetze-im-internet.de/k_sev/BJNR511800965.html); [FAOLEX/Gesetze PDF: Käseverordnung](https://faolex.fao.org/docs/pdf/ger089199.pdf) | Official national legal database; FAOLEX/direct legal PDF |
| **DE_3** | Harness supply chain and actions across sectors to ensure coherence with health | Ordinance on milk products | 1970 | Other(ordinance) | This ordinance regulates the production, packaging, and labeling of dairy products intended for human consumption in Germany, ensuring compliance with set standards and exceptions for milk-based products. | To standardize the production and labeling of milk products for consumer safety. | Dairy products | Not specified |  | [Gesetze im Internet: Milcherzeugnisverordnung (MilchErzV)](https://www.gesetze-im-internet.de/milchv/BJNR011500970.html); [Direct PDF: MilchErzV](https://www.gesetze-im-internet.de/milchv/MilchErzV.pdf) | Official national legal database; direct ordinance PDF |
| **DE_4** | Harness supply chain and actions across sectors to ensure coherence with health | Ordinance on marketing of eggs | 1977 | Other(ordinance) | This ordinance ensures that eggs marketed in Germany comply with both national and European Union standards. | To regulate the marketing and quality of eggs to ensure compliance with standards. | Eggs | Not specified |  | [Gesetze im Internet: Eier-Vermarktungsverordnung (EiMarktV)](https://www.gesetze-im-internet.de/eimarktv/BJNR031380977.html); [Direct PDF: EiMarktV](https://www.gesetze-im-internet.de/eimarktv/EiMarktV.pdf) | Official national legal database; direct ordinance PDF |
| **DE_5** | Harness supply chain and actions across sectors to ensure coherence with health | Federal Act on milk, milk products, margarine and similar products | 1990 | Law | This act regulates the production, handling, and marketing of milk, dairy products, margarine products, mixed fat products, and similar items intended for human consumption. | The legislation aims to ensure that milk and related products meet stringent quality and safety standards, provide clear information to consumers, and comply with European Union regulations. | Milk, dairy products, margarine products, mixed fat products | Not specified |  | [Gesetze im Internet: Milch- und Margarinegesetz (MilchMargG)](https://www.gesetze-im-internet.de/milchmargg/BJNR014710990.html); [Direct PDF: MilchMargG](https://www.gesetze-im-internet.de/milchmargg/MilchMargG.pdf) | Official national legal database; direct statute PDF |
| **DE_6** | Harness supply chain and actions across sectors to ensure coherence with health | Ordinance on commodities | 1992 | Other(ordinance) | This ordinance ensures that materials and objects in contact with food, such as plastics and ceramics, meet strict safety standards, minimizing the migration of harmful substances into food. | To ensure the safety of food-contact materials and protect consumer health. | Food-contact materials | Not specified |  | [Gesetze im Internet: Bedarfsgegenständeverordnung (BedGgstV)](https://www.gesetze-im-internet.de/bedggstv/BJNR008660992.html); [Direct PDF: BedGgstV](https://www.gesetze-im-internet.de/bedggstv/BedGgstV.pdf) | Official national legal database; direct ordinance PDF |
| **DE_7** | Harness supply chain and actions across sectors to ensure coherence with health | Ordinance on maximum residue limits of pesticides and fertilizer, and other substances in and on foodstuffs and tobacco products | 1994 | Other(ordinance) | This ordinance sets maximum residue limits for pesticides, fertilizers, and other substances in or on foodstuffs and tobacco products. | To regulate the maximum amount of residues of pesticides, fertilizers, and other substances in and on foodstuffs and tobacco products. | All food products | Pesticides and fertilizers |  | [Gesetze im Internet: Rückstands-Höchstmengenverordnung (RHmV)](https://www.gesetze-im-internet.de/rhmv_1994/BJNR229900994.html); [Federal Law Gazette record: RHmV 1994](https://www.bgbl.de/xaver/bgbl/start.xav?start=%2F%2F%2A%5B%40attr_id%3D%27bgbl194s2299.pdf%27%5D) | Official national legal database; Federal Law Gazette record |
| **DE_8** | Harness supply chain and actions across sectors to ensure coherence with health | Ordinance on butter and other milk fats | 1997 | Other(ordinance) | This ordinance sets detailed standards for the production, labeling, and marketing of butter and milk fats, including three-quarter fat butter and half-fat butter. It defines specific fat content classifications and quality requirements for these products. | To regulate the production and marketing of butter and milk fats, ensuring compliance with quality standards. | Butter and milk | Fat |  | [Gesetze im Internet: Butterverordnung (ButtV 1997)](https://www.gesetze-im-internet.de/buttv_1997/); [Direct PDF: Butterverordnung](https://www.gesetze-im-internet.de/buttv_1997/ButtV_1997.pdf) | Official national legal database; direct ordinance PDF |
| **DE_9** | Nutrition label standards and regulations on the use of claims and implied claims on food | Act on the Introduction and Use of a Label for Products from Organic Farming | 2001 | Law | This law sets standards for the labeling of organic products, ensuring that only products meeting strict organic criteria can display the eco-label, protecting consumers from misleading claims and promoting trust in organic products. | To regulate the use and implementation of an eco-label for organic farming products. | Organic products | Not specified |  | [Gesetze im Internet: Öko-Kennzeichengesetz (ÖkoKennzG)](https://www.gesetze-im-internet.de/_kokennzg/BJNR344100001.html); [FAOLEX/Gesetze PDF: ÖkoKennzG](https://faolex.fao.org/docs/pdf/ger85364.pdf) | Official national legal database; FAOLEX/direct legal PDF |
| **DE_10** | Harness supply chain and actions across sectors to ensure coherence with health | German Food, Commodities and Feed Code (LFGB) | 2005 | Law | This policy implements EU directives to protect public health and ensure the safety of food and animal feed. It covers production, processing, labeling, and distribution, with strict provisions to prevent health risks and protect consumers from fraud and deception. | To ensure safe use of food, cosmetics, and daily articles; protect consumers from fraud; ensure safe animal feed and high-quality food products. | Food and animal feed products | Not specified |  | [Gesetze im Internet: Lebensmittel- und Futtermittelgesetzbuch (LFGB)](https://www.gesetze-im-internet.de/lfgb/BJNR261810005.html); [BMEL legal reference page: LFGB](https://www.bmleh.de/SharedDocs/ExterneLinks/DE/Rechtsgrundlagen/National/Lebensmittel-undFuttermittelgesetzbuch.html) | Official national legal database; official ministry legal reference |
| **DE_11** | Harness supply chain and actions across sectors to ensure coherence with health | Ordinance on foodstuff provisions to control zoonoses and zoonotic agents | 2007 | Other(ordinance) | This ordinance mandates food business operators to implement measures for the early detection and reporting of zoonoses and zoonotic agents. | To detect and monitor zoonotic diseases and agents in the food supply chain to protect public health. | All food products | Zoonotic agents |  | [Gesetze im Internet: Lebensmittel-Zoonosenverordnung (ZoonoseV)](https://www.gesetze-im-internet.de/zoonosev/BJNR187100007.html); [Direct PDF: ZoonoseV](https://www.gesetze-im-internet.de/zoonosev/ZoonoseV.pdf) | Official national legal database; direct ordinance PDF |
| **DE_12** | Harness supply chain and actions across sectors to ensure coherence with health | Ordinance on hygiene requirements for the production, handling and placing on the market of certain foodstuffs of animal origin | 2007 | Other(ordinance) | This ordinance sets hygiene and safety requirements for the production, processing, and marketing of foodstuffs of animal origin, aligning with EU Regulation No. 853/2004. It covers meat inspections, retail sales, and the handling of primary products. | To implement strict hygiene standards for animal-origin food products throughout the supply chain. | Animal-origin food products | Not specified |  | [Gesetze im Internet: Tierische Lebensmittel-Hygieneverordnung (Tier-LMHV)](https://www.gesetze-im-internet.de/tier-lmhv/BJNR182800007.html); [Direct PDF: Tier-LMHV](https://www.gesetze-im-internet.de/tier-lmhv/Tier-LMHV.pdf) | Official national legal database; direct ordinance PDF |
| **DE_13** | Nutrition label standards and regulations on the use of claims and implied claims on food | Beef Carcass Trade Class Ordinance. | 2008 | Other(ordinance) | This ordinance sets labeling requirements for beef carcasses, specifying category designations, meatiness classes, and fat class indicators. | To standardize beef carcass labeling for transparency in quality and classification. | Beef carcass | Not specified |  | [Gesetze im Internet: Rinderschlachtkörper-Handelsklassenverordnung (RindHKlV)](https://www.gesetze-im-internet.de/rindhklv/BJNR219600008.html); [Gesetze im Internet: RindHKlV § 1](https://www.gesetze-im-internet.de/rindhklv/__1.html) | Official national legal database |
| **DE_14** | Nutrition label standards and regulations on the use of claims and implied claims on food | Organic Farming and the Organic Labelling Law. | 2008 | Law | This law implements EU regulations on organic farming and labeling establishing a framework for the Federal Agency for Agriculture and Food to oversee compliance with EU standards on production, labeling, and processing of organic products. The law includes procedures for approving inspection bodies, licensing for imported organic products, and temporary approvals for agricultural ingredients. | To ensure the authenticity of organic products through strict control and transparency in labeling, while facilitating imports that meet EU organic certification standards. | Organic products | Not specified |  | [Gesetze im Internet: Öko-Landbaugesetz (ÖLG)](https://www.gesetze-im-internet.de/_lg_2009/BJNR235810008.html); [Gesetze im Internet: Öko-Kennzeichengesetz (ÖkoKennzG)](https://www.gesetze-im-internet.de/_kokennzg/BJNR344100001.html) | Official national legal database |
| **DE_15** | Give nutrition education and skills | German National Initiative to Promote Healthy Diets and Physical Activity. | 2008 | Policy | This policy provides actionable recommendations, enhances support structures for healthy living, and fosters collaboration between stakeholders to create a coordinated approach to public health. | To improve dietary habits and physical activity levels nationwide. | All food products | Not specified |  | [Official IN FORM page: National initiative for healthy diet and physical activity](https://www.bmleh.de/DE/themen/ernaehrung/gesunde-ernaehrung/aktionsprogramm-in-form/aktionsprogramm-in-form_node.html); [Official PDF: IN FORM National Action Plan](https://www.bundesgesundheitsministerium.de/fileadmin/Dateien/5_Publikationen/Praevention/Broschueren/IN_FORM_Nationaler_Aktionsplan_zur_Praevention_von_Fehlernaehrung__Bewegungsmangel__UEbergewicht_und_damit_zusammenhaengenden_Krankheiten.pdf) | Official ministry programme page; official policy PDF |
| **DE_16** | Use economic tools to address food affordability and purchase incentives | Coffee Tax Act | 2009 | Law | This law imposes taxes on coffee products, including 2.19 euros/kg for green coffee beans and 4.78 euros/kg for both roasted and instant coffee. | To generate revenue for the federal government and regulate the coffee market | Coffee products | Not specified |  | [Gesetze im Internet: Kaffeesteuergesetz (KaffeeStG)](https://www.gesetze-im-internet.de/kaffeestg_2009/BJNR191900009.html); [Direct PDF: KaffeeStG](https://www.gesetze-im-internet.de/kaffeestg_2009/KaffeeStG.pdf) | Official national legal database; direct statute PDF |
| **DE_17** | Harness supply chain and actions across sectors to ensure coherence with health | Ordinance on the Limitation of Contaminants in Foodstuffs (Contaminants Ordinance - KmV) | 2010 | Other(ordinance) | This ordinance prohibits the circulation of foodstuffs exceeding maximum contaminant levels, including mycotoxins. It covers definitions, treatment of contaminated food, labeling, and official sampling during controls. | To mitigate the presence of contaminants in foodstuff. | All food products | Contaminants |  | [Gesetze im Internet: Kontaminanten-Verordnung (KmV)](https://www.gesetze-im-internet.de/kmv/BJNR028700010.html); [Direct PDF: KmV](https://www.gesetze-im-internet.de/kmv/KmV.pdf) | Official national legal database; direct ordinance PDF |
| **DE_18** | Harness supply chain and actions across sectors to ensure coherence with health | Act on the implementation of unions legislation on the school program for fruit, vegetables and milk. | 2016 | Law | The legislation implements the EU's school fruit, vegetable, and milk program in Germany, outlining federal and state responsibilities for program management. | To implement EU regulations regarding school programs for distributing fruits, vegetables, and milk to children. | School food products (fruit, vegetable, and milk) | Not specified | X | [Gesetze im Internet: Landwirtschaftserzeugnisse-Schulprogrammgesetz (LwErzgSchulproG)](https://www.gesetze-im-internet.de/lwerzgschulprog/BJNR285800016.html); [DIP Bundestag legislative record: school programme for fruit, vegetables and milk](https://dip.bundestag.de/vorgang/gesetz-zur-durchf%C3%BChrung-unionsrechtlicher-vorschriften-%C3%BCber-das-schulprogramm-f%C3%BCr-obst/76151) | Official national legal database; parliamentary legislative record |
| **DE_19** | Nutrition label standards and regulations on the use of claims and implied claims on food | Ordinance implementing EU provisions concerning food information to consumers. | 2017 | Other(ordinance) | This ordinance enforces EU Regulation No. 1169/2011, mandating accurate labeling and provision of essential information on food products for consumers. | To ensure that consumers have access to essential information about the food products they purchase and consume. | All food products | Not specified |  | [Gesetze im Internet: Lebensmittelinformations-Durchführungsverordnung (LMIDV)](https://www.gesetze-im-internet.de/lmidv/BJNR227210017.html); [Direct PDF: LMIDV](https://www.gesetze-im-internet.de/lmidv/LMIDV.pdf) | Official national legal database; direct ordinance PDF |
| **DE_20** | Harness supply chain and actions across sectors to ensure coherence with health | National reduction and innovation strategy for sugar, fats and salt in finished products | 2018 | Policy | This policy aims to reduce sugar, fats, and salt levels in processed foods through a scientifically based strategy. Key targets include a 20% sugar reduction in children's cereals, a 15% reduction in sugar in sweetened dairy products, and lower salt levels in bread and frozen pizzas through agreements with manufacturers. | To reduce sugar, fats, and salt in processed foods within a single, coordinated framework. | Processed foods | Sugar, fats, and salt | X | [Official BMEL PDF: National Reduction and Innovation Strategy](https://www.bmleh.de/SharedDocs/Downloads/DE/_Ernaehrung/Reduktionsstrategie/NationaleReduktionsInnovationsstrategie-Layout.pdf?__blob=publicationFile&v=5); [Official BMEL PDF: second interim report on the strategy](https://www.bmleh.de/SharedDocs/Downloads/DE/Broschueren/nri-zwischenbericht-2.pdf?__blob=publicationFile&v=7) | Official ministry strategy PDF; official monitoring report |
| **DE_21** | Nutrition label standards and regulations on the use of claims and implied claims on food | Nutri-Score Labeling Policy | 2019 | Policy | This front-of-pack labeling system provides consumers with easy-to-read nutritional information using a color-coded scale (from green/A to red/E). It helps consumers quickly compare the nutritional value of pre-packaged foods. | To facilitate informed food choices through clear labeling. | All food products | Nutrients |  | [Gesetze im Internet: LMIDV § 4a extended nutrition labelling / Nutri-Score](https://www.gesetze-im-internet.de/lmidv/__4a.html); [Federal Law Gazette PDF: First Ordinance amending LMIDV, Nutri-Score](https://media.offenegesetze.de/bgbl1/2020/bgbl1_2020_49.pdf) | Official national legal database; Federal Law Gazette PDF |
| **DE_22** | Harness supply chain and actions across sectors to ensure coherence with health | Raw Milk Quality Ordinance. | 2021 | Other(ordinance) | This ordinance sets strict quality standards for raw milk production, collection, transportation, and processing. | To safeguard public health by enforcing high standards in the raw milk supply chain. | Raw milk | Not specified |  | [Gesetze im Internet: Rohmilchgüteverordnung (RohmilchGütV)](https://www.gesetze-im-internet.de/rohmilchg_tv/BJNR004710021.html); [Official legal PDF: RohmilchGütV](https://www.milchbauernservice.de/fileadmin/user_upload/Rohmilchgueteverordnung_11.01.2021.pdf) | Official national legal database; direct legal PDF |
| **DE_23** | Improve the nutritional quality of the food supply | Regulation on the adaptation of national legislation to Union rules on flavourings and foodstuffs containing flavourings. | 2021 | Other (regulation) | This regulation enforces EU standards on the use and labeling of flavorings in food, prohibiting certain flavors in infant food and requiring detailed labeling of smoke flavors and natural flavors. It mandates transparency in flavor content percentages and restricts the term “natural” to cases that meet EU Regulation (EC) No. 1334/2008. | To ensure compliance with EU regulations on flavoring use and labeling for consistent consumer information. | All food products | Flavorings | X | [Gesetze im Internet: Aromendurchführungsverordnung (AromenDV)](https://www.gesetze-im-internet.de/aromendv/BJNR472310021.html); [BMEL legal text page: AromenDV adaptation regulation](https://www.bmleh.de/SharedDocs/Gesetzestexte/DE/AromenDV.html) | Official national legal database; official ministry legal text page |
| **DE_24** | Harness supply chain and actions across sectors to ensure coherence with health | Regulation on the implementation of EU regulations on food additives (food additive implementation regulation - LMZDV). | 2021 | Other (regulation) | This regulation enforces EU standards on food additives, defining permissible substances, approval procedures, and labeling requirements. | To protect public health by regulating safe and transparent use of food additives in line with EU standards. | All food products | Additives |  | [Gesetze im Internet: Lebensmittelzusatzstoff-Durchführungsverordnung (LMZDV)](https://www.gesetze-im-internet.de/lmzdv/BJNR136210021.html); [Gesetze im Internet: LMZDV table of contents](https://www.gesetze-im-internet.de/lmzdv/) | Official national legal database |
| **DE_25** | Nutrition label standards and regulations on the use of claims and implied claims on food | Law Amending the Organic Farming Law and the Organic Labelling Law. | 2021 | Law | This law introduces stricter labeling requirements for organic products used in collective catering facilities, ensuring clearer and more standardized information for consumers. | To include specific regulations for communal dining environments, such as schools and hospitals and to guarantee transparency and uniformity in the labeling of organic products in collective catering settings. | Organic products | Not specified | X | [Federal Law Gazette reference: Law amending ÖLG and ÖkoKennzG, 27 July 2021](https://dejure.org/BGBl/2021/BGBl._I_S._3176); [Bundestag page: new requirements for labelling organic products](https://www.bundestag.de/dokumente/textarchiv/2021/kw20-de-oeko-kennzeichen-842724) | Federal Law Gazette record; parliamentary legislative information |
| **DE_26** | Nutrition label standards and regulations on the use of claims and implied claims on food | Organic Out-of-Home Catering Regulation (Bio-AHVV). | 2023 | Other (regulation) | This regulation sets standards for the use, control, and labeling of organic ingredients in catering services. It ensures that enterprises clearly label the percentage of organic food offered, adhering to certification requirements. | To promote the use of organic food in catering services and ensure transparency for consumers. | Organic products | Not specified |  | [Gesetze im Internet: Bio-AHVV](https://www.gesetze-im-internet.de/bio-ahvv/BJNR1090B0023.html); [ECOLEX/FAOLEX record: Organic Out-of-Home Catering Regulation (Bio-AHVV)](https://www.ecolex.org/fr/details/legislation/organic-out-of-home-catering-regulation-bio-ahvv-lex-faoc223481/?page=6&q=23%2F2018&sortby=newest&type=legislation) | Official national legal database; ECOLEX/FAOLEX legal record |
| **GR_1** | Improve the nutritional quality of the food supply | Food and Drinks Code of Greece | 1971 | Law | This law sets precise limits on salt content in various products: for bread, the salt limit is less than 1.5%. Tomato juice can contain a maximum of 1% salt. For tomato concentrates, the salt content varies ranging from a maximum of 2% to 5%, depending on the product’s tomato solids and packaging size. | To regulate and limit the amount of salt in food products. | All food products | Salt |  | Ministry of Justice / Isokratis consolidated PDF: Κώδικας Τροφίμων και Ποτών: <https://ministryofjustice.gr/wp-content/uploads/2019/10/%CE%9A%CF%8E%CE%B4%CE%B9%CE%BA%CE%B1%CF%82-%CE%A4%CF%81%CE%BF%CF%86%CE%AF%CE%BC%CF%89%CE%BD-%CE%BA%CE%B1%CE%B9-%CE%A0%CE%BF%CF%84%CF%8E%CE%BD.pdf> AADE: Food and Drinks Code current publications page: <https://www.aade.gr/en/chemical-laboratories/food-materials-contact-food/chemical-laboratory/food-and-drinks-code> | Official consolidated legal text; official government code page |
| **GR_2** | Set incentives and rules to create a healthy retail and food service environment | The food code Ministerial Decision 1100/1987 | 1987 | Law | This law establishes comprehensive safety and hygiene standards for foodstuffs and beverages sold, regulating the production, treatment, labeling, and market placement. | To safeguard food safety and hygiene in the production and sale of food and beverages. | All food products | Not specified |  | ECOLEX specific record: Ministerial Decree No. 1100/87 codifying the provisions of the Food Code: <https://www.ecolex.org/fr/details/legislation/ministerial-decree-no-110087-codifying-the-provisions-of-the-food-code-lex-faoc106642/> Ministry of Justice / Isokratis consolidated PDF: Κώδικας Τροφίμων και Ποτών: <https://ministryofjustice.gr/wp-content/uploads/2019/10/%CE%9A%CF%8E%CE%B4%CE%B9%CE%BA%CE%B1%CF%82-%CE%A4%CF%81%CE%BF%CF%86%CE%AF%CE%BC%CF%89%CE%BD-%CE%BA%CE%B1%CE%B9-%CE%A0%CE%BF%CF%84%CF%8E%CE%BD.pdf> | ECOLEX specific legislation record; official consolidated legal text |
| **GR_3** | Improve the nutritional quality of the food supply | Mandatory Salt Limits for Bread, Tomato Juice, and Tomato Concentrates | 2011 | Law | This law establishes mandatory limits on salt content for bread, tomato juice, and tomato concentrates aiming to reduce salt levels in these commonly consumed products. | To improve the nutritional quality of the food supply by reducing salt content. | Bread, tomato juice, and tomato concentrates | Salt |  | Ministry of Justice / Isokratis consolidated PDF: Food and Drinks Code provisions: <https://ministryofjustice.gr/wp-content/uploads/2019/10/%CE%9A%CF%8E%CE%B4%CE%B9%CE%BA%CE%B1%CF%82-%CE%A4%CF%81%CE%BF%CF%86%CE%AF%CE%BC%CF%89%CE%BD-%CE%BA%CE%B1%CE%B9-%CE%A0%CE%BF%CF%84%CF%8E%CE%BD.pdf> World Action on Salt, Sugar & Health: Greece mandatory salt targets summary: <https://www.worldactiononsalt.com/worldaction/reformulation/salt-reduction/> | Official consolidated legal text; policy database/technical summary |
| **GR_4** | Offer healthy food and set standards in public institutions and other specific settings | Health Ordinance Y1c/G.P. house 73828/1 -8-06 regarding hygiene rules and determining products available from public and private school canteens | 2013 | Other(ordinance) | The ordinance aims to protect public health by addressing obesity and chronic diet-related diseases through the establishment of a school canteen, serving as an educational tool for promoting healthy eating principles. | To promote healhty dietary habits in school environment. | School food products | Not specified | X | Athens Bar Association legal information bank: YA DY1G/G.P./OIK.93828/2006, FEK 1183/B/31.8.2006: <https://www.dsanet.gr/Epikairothta/Nomothesia/ya93828_06.htm> e-Nomothesia specific page: YA DY1G/G.P./OIK.93828/2006: <https://www.e-nomothesia.gr/kat-ekpaideuse/skholika-kulikeia/ya-du1ggp-oik-93828-2006.html> | Specific legal-text database page; specific legal information page |
| **GR_5** | Offer healthy food and set standards in public institutions and other specific settings | 2135/2013 Rules for school hygiene, determination of available products | 2013 | Law | This law mandates that only fresh, nutritious, and minimally processed foods be sold in school canteens. It specifies allowed items, focusing on fruits, vegetables, dairy, and whole grains, while banning unhealthy options like sugary drinks, processed snacks, and genetically modified foods | To ensure that food and beverages sold in school canteens promote healthy eating. | School food products | Nutrients, processed and GMO ingredients | X | Greek Ministry of Health direct PDF: Υ.Δ. Υ1γ Γ.Π.οικ.81025 (ΦΕΚ 2135 Β 2013): <https://www.moh.gov.gr/articles/health/dieythynsh-dhmosias-ygieinhs/tmhma-ygeionomikwn-kanonismwn-dhmosias-ygeias/c336-nomothesia/ethnikh-nomothesia/ypoyrgeio-ygeias-tmhma-ygeionomikwn-kanonismwn/1561-trofima-sta-sxolika-kylikeia?fdl=9190> Greek Ministry of Health school-canteen legislation page: <https://www.moh.gov.gr/articles/health/dieythynsh-dhmosias-ygieinhs/tmhma-ygeionomikwn-kanonismwn-dhmosias-ygeias/c336-nomothesia/ethnikh-nomothesia/ypoyrgeio-ygeias-tmhma-ygeionomikwn-kanonismwn/1561-trofima-sta-sxolika-kylikeia> | Official government PDF; official ministry policy/legal page |
| **GR_6** | Improve the nutritional quality of the food supply | Salt Reduction Strategy 2016-2020 | 2016 | Policy | This policy built on four key pillars: assessing salt intake in the population, raising public awareness through targeted campaigns, setting realistic salt content limits for food products, and reformulating products to reduce salt. It also includes systematic monitoring and evaluation to track progress toward the salt reduction goals. | To improve public health by decreasing the population's salt consumption. | All food products | Salt |  | Hellenic Food Authority (EFET) direct PDF: Στρατηγική Μείωσης Αλατιού 2016-2020: <https://www.efet.gr/files/stratigiki_meiosis_alatiou.pdf> WHO NLiS/GINA Greece profile entry listing Salt Reduction Strategy 2016-2020: <https://www.who.int/docs/default-source/nutritionlibrary/nlis-pdf-reports/nlis-profile-grc.pdf?sfvrsn=b90812dc_2> | Official agency strategy PDF; WHO policy database profile |
| **GR_7** | Offer healthy food and set standards in public institutions and other specific settings | Program for the promotion of fruit and vegetable consumption in schools | 2016 | Policy | This policy sets out the targets to gradually increase the portion of fruits and vegetables in children's diets during the formative years of their eating habits to promote heathy lifestyle. | This policy aims to boost both short-term and long-term fruit and vegetable consumption among children, thereby enhancing their overall dietary habits. | Children food products (fruits and vegetables) | Not specified | X | European Commission / Greece school scheme strategy 2017-2023 PDF: <https://ec.europa.eu/info/sites/default/files/food-farming-fisheries/key_policies/documents/el-school-scheme-strategy-2017-23_en.pdf> WHO NLiS/GINA Greece profile entry listing the programme: <https://www.who.int/docs/default-source/nutritionlibrary/nlis-pdf-reports/nlis-profile-grc.pdf?sfvrsn=b90812dc_2> | Official EU/national school-scheme strategy document; WHO policy database profile |
| **GR_8** | Harness supply chain and actions across sectors to ensure coherence with health | Greece’s National Action Plan on Food Reformulation | 2017 | Policy | This plan supports food producers in creating healthier products, educates consumers through public campaigns, and enforces nutritional standards through legislation. | To reduce salt, trans fats, and added sugars in foods by setting upper limits for these nutrients. | All food products | Nutrients |  | Greek Ministry of Health direct PDF: Greece's National Action Plan on Food Reformulation: <https://www.moh.gov.gr/articles/health/dieythynsh-dhmosias-ygieinhs/metadotika-kai-mh-metadotika-noshmata/c388-egkyklioi/5614-efarmogh-sxedioy-drashs-gia-thn-anasynthesh-twn-proiontwn-trofimwn?fdl=15705> | Official government action-plan PDF |
| **HU_1** | Offer healthy food and set standards in public institutions and other specific settings | Decree 30/1981. (XII.30.) | 1981 | Other (decree) | The decree mandates food must pass tests and be assessed by an official veterinarian, with game inspection either individual or batch-based, and one-time or continuous. | To ensure the safety and quality of food products related to animal origin. | Animal-origin Food products | Not specified |  | Jogkódex specific legal text: 30/1981. (XII.30.) MÉM-EüM joint decree: <https://jogkodex.hu/jsz/1981_30_mem_eum_rendelet_4920026> Jogtár direct PDF: 30/1981. (XII.30.) MÉM-EüM joint decree: <https://net.jogtar.hu/getpdf?docid=98100030.MEM&printTitle=30%2F1981.+%28XII.+30.%29+M%C3%89M-E%C3%BCM+egy%C3%BCttes+rendelet&targetdate=19970701> | Specific national legal database page; direct legal PDF |
| **HU_2** | Offer healthy food and set standards in public institutions and other specific settings | Annex No. 43 of FVM Decree No. 15/2001 (III. 3.) | 2001 | Other (decree) | The decree supports quality game meat production and applies to authorized hunters who sell specified quantities of killed game to consumers or businesses. | To support quality game meat production and regulate its sale by authorized hunters. | Game meat | Not specified |  | Jogkódex specific legal text: 15/2001. (III.3.) FVM decree: <https://jogkodex.hu/jsz/2001_15_fvm_rendelet_5990029> | Specific national legal database page |
| **HU_3** | Offer healthy food and set standards in public institutions and other specific settings | FVM Decree No. 9/2002 (I. 23.) | 2002 | Other (decree) | The decree mandates veterinary meat inspection for both shot and farmed game intended for public consumption. | To ensure safety and quality standards. | Shot and farmed game meat | Not specified |  | FAOLEX direct PDF: 9/2002. (I.23.) FVM decree: <https://faolex.fao.org/docs/pdf/hun31851.pdf> | FAOLEX direct legal PDF |
| **HU_4** | Harness supply chain and actions across sectors to ensure coherence with health | Act No. XLVI of 2008 on food chain and its control | 2008 | Law | This law reduces risks in the food chain, promotes local food production, and addresses animal and plant health. The law also sets basic rules on food labeling and marketing. | To protect consumer health, ensure safe food production, and support international food trade. | All food products | Not specified |  | ECOLEX specific record: Act No. XLVI of 2008 on food chain and its control: <https://www.ecolex.org/details/legislation/act-no-xlvi-of-2008-on-food-chain-and-its-control-lex-faoc098048/> NÉBIH English legislation page: Act No. XLVI of 2008 on food chain safety: <https://portal.nebih.gov.hu/web/english/-/legislati-3> | ECOLEX specific legislation record; official national food-chain authority page |
| **HU_5** | Improve the nutritional quality of the food supply | Decree No. 152 of 2009 (Codex Alimentarius Hungaricus) | 2009 | Other (decree) | This Decree, part of the Codex Alimentarius Hungaricus, regulates the salt content in bread and other bakery products. It progressively reduces the maximum allowable salt levels to improve public health. | To reduce salt intake by setting mandatory limits on salt content in bread and bakery products. | Bread and other bakery products | Salt |  | Jogtár specific legal text: 152/2009. (XI.12.) FVM decree: <https://net.jogtar.hu/jogszabaly?docid=a0900152.fvm> ECOLEX specific record: Decree No. 152 of 2009 on the binding provisions of the Codex Alimentarius Hungaricus: <https://www.ecolex.org/details/legislation/decree-no-152-of-2009-xi-12-fvm-of-the-ministry-of-agriculture-and-rural-development-on-the-binding-provisions-of-the-codex-alimentarius-hungaricus-lex-faoc109822/> | Official legal database; ECOLEX specific legislation record |
| **HU_6** | Harness supply chain and actions across sectors to ensure coherence with health | Hungarian National Nutrition Policy 2010-2013 | 2010 | Policy | This policy includes actions to improve public catering standards, limit the sale of unhealthy foods in schools, promote water consumption, reduce salt, sugar, and fat content in foods, and regulate trans fats. It also emphasizes consumer information and integrates nutrition education into school curricula. | To improve public health by enhancing food quality in public catering, promoting healthier food choices, and reducing obesity-related diseases. | All food products | Nutrients | X | WHO NLiS/GINA Hungary profile entry: Hungarian National Nutrition Policy 2010-2013: <https://apps.who.int/nutrition/landscape/report.aspx?iso=hun&print=1> Public Health journal PDF discussing Hungary’s 2010-2013 National Nutrition Policy Action Plan: <https://nepegeszsegugyi-egyesulet.hu/sites/default/files/2020-08/91_2_2013.pdf> | WHO policy database profile; peer-reviewed/public-health journal source |
| **HU_7** | Use economic tools to address food affordability and purchase incentives | Hungarian National Public Health Product Tax (NETA) | 2011 | Law | This tax applied to food products with unhealthy levels of sugar, salt, and stimulants: Salty snacks with more than 1 g of salt per 100 g, condiments with more than 5 g of salt per 100 g, and flavorings with more than 15 g of salt per 100 g are taxed at a rate of HUF 250/kg (US$ 0.89/kg). | To reduce the consumption of unhealthy foods and promote healthier nutrition | All food products | Sugar, salt, and stimulants |  | Jogtár specific legal text: Act CIII of 2011 on Public Health Product Tax: <https://net.jogtar.hu/jogszabaly?docid=a1100103.tv> NJT historical legal text: 2011. évi CIII. törvény: <https://njt.hu/jogszabaly/2011-103-00-00.1> | Official legal database; National Legislation Database |
| **HU_8** | Improve the nutritional quality of the food supply | Codex Alimentarius Hungaricus; Foods preserved by way of heat treatment; Modified Salt Content for Bread and Bakery Products | 2012 | Policy | This policy regulates the salt content in heat-treated vegetables preserved in brine and bread. For green peas, green beans, and sweet corn, the content must not exceed 1.5%, while for other heat-treated vegetables, it must not exceed 2.0%. For white bread, salt content must be between 1.3% and 2.5% from 2015 and reduced to a maximum of 2.35% by 2018. | To regulate the sodium chloride (NaCl) content in heat-treated vegetables and bread products ensuring quality standards for salt concentration. | Heat-treated vegetables and bread products | Salt |  | Jogtár specific legal text: 152/2009. (XI.12.) FVM decree: <https://net.jogtar.hu/jogszabaly?docid=a0900152.fvm> Hungarian Food Codex bakery-products specification PDF (Annex 39 / MÉ 1-3/16-1): <https://elelmiszerlanc.kormany.hu/download/4/4a/02000/1_3_16_1%20M%C3%89%20S%C3%BCt%C5%91ipari%20term%C3%A9kek_%202018%20janu%C3%A1r%201_m%C3%B3dos%C3%ADt%C3%A1s.pdf> | Official legal database; official Food Codex specification PDF |
| **HU_9** | Improve the nutritional quality of the food supply | Decree No. 71/2013 (Trans Fat Regulation) | 2014 | Other (decree) | The decree bans food products with over 2% trans-fat. For processed foods, trans fat must not exceed 4 grams per 100 grams if total fat is under 20%, and 10 grams if under 3%. | To set the maximum allowed trans-fat content in food products, reducing harmful trans fatty acid intake. | Processed food products | Trans fatty acids |  | NÉBIH direct PDF / guidance citing 71/2013. (XI.20.) EMMI decree: <https://portal.nebih.gov.hu/documents/10182/406632/4-6-4_Transzzs%C3%ADrsavak.pdf/e289ba45-3002-44cf-b4a6-6878713f46f8> WHO NLiS/GINA Hungary profile entry listing 71/2013 EMMI decree: <https://apps.who.int/nutrition/landscape/report.aspx?iso=hun&print=1> | Official food-chain authority PDF/guidance; WHO policy database profile |
| **HU_10** | Offer healthy food and set standards in public institutions and other specific settings | Decree on nutrition and health regulations for public catering | 2015 | Other (decree) | The policy provides guidelines for menu planning and meal preparation to ensure balanced, age-appropriate meals that comply with food safety and display allergens and nutritional information. | To ensure public meals are nutritious, meet dietary standards, and suit all age groups' energy needs. While the decree does not directly mandate labeling requirements, it does include provisions that indirectly affect how food information is presented to consumers. | All food products | Not specified |  | Jogtár specific legal text: 37/2014. (IV.30.) EMMI decree: <https://net.jogtar.hu/jogszabaly?docid=a1400037.emm> NJT specific legal text: 37/2014. (IV.30.) EMMI decree: <https://njt.hu/jogszabaly/2014-37-20-5H.7> | Official legal database; National Legislation Database |
| **HU_11** | Harness supply chain and actions across sectors to ensure coherence with health | National Plant Protection Action Plan 2019-2023. | 2019 | Policy | This plan establishes quantitative objectives and measures to reduce the risks of pesticide use on human health and the environment. It promotes integrated pest management and alternative approaches to minimize agricultural risks from plant protection products. | To ensure food security by reducing the risks associated with pesticide use. | Plant protection products | Pesticides |  | NÉBIH direct PDF: Nemzeti Növényvédelmi Cselekvési Terv 2019-2023: <https://portal.nebih.gov.hu/documents/10182/998660/NCST_%2B2019.pdf/dc1dd23c-f47a-318c-d623-31d44683247c> | Official national authority action-plan PDF |
| **HU_12** | Harness supply chain and actions across sectors to ensure coherence with health | Decree on the production and placing on the market of food and materials and articles intended to come in contact with food. | 2021 | Other (decree) | This Decree establishes safety and hygiene standards for the production and placing on the market of food products and materials intended to come in contact with food, aligning with EU regulations to ensure compliance and public health protection. | To ensure the safety of food and materials, harmonizing with European food safety standards. | Food-contact materials | Not specified |  | Jogtár specific legal text: 20/2021. (V.17.) AM decree: <https://net.jogtar.hu/jogszabaly?docid=a2100020.am> FAOLEX direct PDF: 20/2021. (V.17.) AM decree: <https://faolex.fao.org/docs/pdf/hun205022.pdf> ECOLEX specific record: Decree on production and placing on the market of food and food-contact materials: <https://www.ecolex.org/fr/details/legislation/decree-on-the-production-and-placing-on-the-market-of-food-and-materials-and-articles-intended-to-come-into-contact-with-food-lex-faoc205022/> | Official legal database; FAOLEX direct legal PDF; ECOLEX specific legislation record |
| **IE_1** | Harness supply chain and actions across sectors to ensure coherence with health | Health Act 1947 | 1947 | Law | This legislation, within Part V, is dedicated to food and drinks (excluding drugs and water). It establishes a framework to maintain and improve public health standards, empowering health authorities to regulate and act in the interest of community health. | To create a robust legal framework for protecting public health and regulating health services. | All food products | Not specified |  | [Irish Statute Book: Health Act, 1947](https://www.irishstatutebook.ie/eli/1947/act/28/enacted/en/html); [Law Reform Commission Revised Act: Health Act 1947](https://revisedacts.lawreform.ie/eli/1947/act/28/revised/en/html) | Official national legal database; revised statute consolidation |
| **IE_2** | Harness supply chain and actions across sectors to ensure coherence with health | Changing cardiovascular health | 2010 | Policy | This policy sets targets to improve cardiovascular health by promoting healthier eating habits. Goals include increasing fruit and vegetable intake, reducing fat and sugar consumption, and limiting salt intake. It aims to reduce saturated fats and trans fats, with specific goals to lower daily salt intake to 6 grams for adults and focus on reducing salt in children. | To improve public health by promoting healthier eating habits and reducing salt consumption | All food products | Nutrients | X | [Department of Health: Changing Cardiovascular Health, National Cardiovascular Health Policy 2010-2019](https://www.gov.ie/en/department-of-health/publications/changing-cardiovascular-health-national-cardiovascular-health-policy-2010-2019/); [Official PDF: changing_cardiovascular_health.pdf](https://health.gov.ie/wp-content/uploads/2016/04/changing_cardiovascular_health.pdf) | Official government policy page; official policy PDF |
| **IE_3** | Improve the nutritional quality of the food supply | Salt Reduction Programme (SRP) | 2010 | Policy | This program sets sodium content limits for various foods: cooked uncured meat products (600 mg Na/100 g), black and white puddings (600 mg Na/100 g), bacon (max. 1300 mg Na/100 g), sausages (550 mg Na/100 g), bread (450 mg Na/100 g on average), and burgers (400 mg Na/100 g). It also includes salt equivalency labeling and advises against the use of salt substitutes. | To standardize sodium content in food products and improve public health through consistent product labeling. | Cooked uncured meat, puddings, bacon, sausages, bread and burgers | Sodium |  | [Lenus/HSE record: Salt reduction programme 2010-2011](https://www.lenus.ie/entities/publication/256abd03-db28-4f77-a328-582b09f0d456); [FSAI PDF: Salt and Health review noting FSAI salt reduction programme](https://www.fsai.ie/getattachment/ab9a196e-258a-40d5-b857-7f0308bb96de/10507) | National health repository record; Food Safety Authority of Ireland scientific/policy PDF |
| **IE_4** | Restrict food advertising and other forms of commercial promotion | Children’s Commercial Communications Code | 2010 | Policy | This policy sets standards for commercials targeting children, preventing the promotion of unhealthy lifestyles and misleading nutritional information. It includes specific rules for foods high in fat, sugar and salt, fast food, and confectionery, as well as restrictions on the use of celebrities in children's ads to promote responsible advertising and support a balanced diet. | To protect children from inappropriate or harmful commercial communications. | Children food products | Nutrients | X | [Coimisiún na Meán: Children’s Commercial Communications Code](https://www.cnam.ie/industry-and-professionals/codes-legislation/codes-and-rules/childrens-commercial-communications-code/); [Official PDF: Children’s Commercial Communications Code](https://www.cnam.ie/app/uploads/2024/12/Childrens-Commercial-Communications-Code-December-2024.pdf) | Official media regulator code page; official code PDF |
| **IE_5** | Restrict food advertising and other forms of commercial promotion | Code of Standards for Advertising and Marketing Communications | 2016 | Law | This law defines that marketing communications for food must avoid misleading claims, promote healthy consumption, accurately represent product characteristics, and ensure that advertising for high-fat, salt, and sugar (HFSS) products does not target or appeal to children under 15. | To regulate food marketing and ensure that advertisements promote healthy consumption and avoid targeting children with unhealthy products. | Children food products | Nutrients | X | [Advertising Standards Authority Ireland: The Code](https://adstandards.ie/asa-code/); [Ad Standards Ireland PDF: Code of Standards for Advertising and Marketing Communications, 7th edition](https://adstandards.ie/wp-content/uploads/2024/03/ASAI-CODE_7th-Edition_Revision_2021.pdf) | Self-regulatory authority code page; direct code PDF |
| **IE_6** | Offer healthy food and set standards in public institutions and other specific settings | Nutrition Standards for School Meals | 2018 | Policy | This policy sets strict guidelines to limit high-fat, high-sugar, and high-salt foods in school meals, including processed and fried items. It excludes sugar-sweetened drinks, promotes milk and water, and limits fruit juice to one serving per day. | To promote healthier food choices and improve the quality of meals provided in schools or organizations funded by the School Meals (Local Projects) Scheme. | School food products | Fat, Sugar and Salt | X | [Department of Social Protection: School Meals](https://www.gov.ie/en/department-of-social-protection/publications/school-meals/); [Official PDF: Nutrition Standards for School Meals](https://assets.gov.ie/static/documents/nutritional-standards-for-school-meals.pdf) | Official government programme page; official standards PDF |
| **IE_7** | Improve the nutritional quality of the food supply | European Union (Food Intended for Infants and Young Children, Food for Special Medical Purposes, and Total Diet Replacement for Weight Control) (Amendment) Regulations 2022 (S.I. No. 111/2022). | 2022 | Other (regulation) | This regulation authorizes the use of calcium-L-methylfolate as a source of folate in infant formula, follow-on formula, processed cereal-based food, and baby food. | To improve the nutritional quality of food products for infants and young children. | Infant food products | Calcium-L-methylfolate | X | [Irish Statute Book: S.I. No. 111/2022](https://www.irishstatutebook.ie/eli/2022/si/111/made/en/print); [Irish Statute Book PDF: S.I. No. 111/2022](https://www.irishstatutebook.ie/eli/2022/si/111/made/en/pdf) | Official national legal database; statutory instrument PDF |
| **IT_1** | Harness supply chain and actions across sectors to ensure coherence with health | Law 30 April 1962, n. 283 (amendment of articles 242, 243, 247, 250 and 262 of the consolidated text of sanitary laws, approved with royal decree 27 July 1934, n. 1265) - Hygienic requirements for the production and sale of food substances and beverages. | 1962 | Law | This law defines hygienic and sanitary rules for the production, processing, and sale of food and beverages. | This law defines hygienic and sanitary rules for the production, processing, and sale of food and beverages. | All food products | Not specified |  | [Normattiva: Legge 30 aprile 1962, n. 283](https://www.normattiva.it/uri-res/N2Ls?urn%3Anir%3Astato%3Alegge%3A1962-04-30%3B283=); [Normattiva consolidated text: Legge 30 aprile 1962, n. 283](https://www.normattiva.it/eli/id/1962/06/04/062U0283/CONSOLIDATED/20210311) | Official national legal database; consolidated legal text |
| **IT_2** | Nutrition label standards and regulations on the use of claims and implied claims on food | Decree of June 1, 1998, No. 518 Regulation implementing Directive 96/4/EC of the Commission of February 26, 1996, which amends Directive 91/321/EEC on infant formulae and follow-on formulae | 1999 | Other (decree) | The decree mandates that labeling for these products must include specific information on energy value, protein, carbohydrate, and fat content, as well as the average amounts of minerals and vitamins. For follow-on formulae, labeling may also include nutritional information in percentage terms if the levels meet specific reference values. Products conforming to previous regulations may be sold until March 31, 1999, or until existing stock is depleted. | The aim of this decree is to update regulations to ensure that infant formulae and follow-on formulae are safe and properly labeled in compliance with European standards. | Infant food products | Nutrients | X | [Normattiva: Decreto 1 giugno 1998, n. 518](https://www.normattiva.it/uri-res/N2Ls?urn%3Anir%3Aministero.sanita%3Adecreto%3A1998-06-01%3B518=) | Official national legal database |
| **IT_3** | Nutrition label standards and regulations on the use of claims and implied claims on food | Decreto 19 novembre 2020 | 2020 | Other (regulation) | NutrInform Battery visually shows the percentage of energy and nutrients in a food relative to the recommended portion size. | To provide a clear visual representation of energy and nutrient percentages relative to portion size. | All food products | Nutrients |  | [Gazzetta Ufficiale: Decreto 19 novembre 2020, NutrInform Battery](https://www.gazzettaufficiale.it/atto/serie_generale/caricaDettaglioAtto/originario?atto.codiceRedazionale=20A06617&atto.dataPubblicazioneGazzetta=2020-12-07&elenco30giorni=false); [Gazzetta Ufficiale issue n. 304, 7 December 2020](https://www.gazzettaufficiale.it/gazzetta/serie_generale/caricaDettaglio?dataPubblicazioneGazzetta=2020-12-07&numeroGazzetta=304) | Official gazette legal record |
| **IT_4** | Nutrition label standards and regulations on the use of claims and implied claims on food | Decree of 28/12/2021 of the Minister of Agricultural Food and Forestry Policy in agreement with the Minister of Economic Development and the Minister of Health. | 2021 | Other (decree) | The decree mandates labeling for rice, durum wheat pasta, tomato products, tomato-based sauces, all milk and dairy products, minced and mechanically separated pork, pork preparations, and pork products. | To mandate labeling for specific food products to ensure transparency. | Rice, durum wheat pasta, tomato products, tomato-based sauces, all milk and dairy products, minced and mechanically separated pork, pork preparations, and pork products. | Not specified |  | [Gazzetta Ufficiale: Decreto 28 dicembre 2021 - Proroga della etichettatura di origine obbligatoria](https://www.gazzettaufficiale.it/eli/id/2022/02/08/22A00804/sg); [Gazzetta Ufficiale article view: Decreto 28 dicembre 2021](https://www.gazzettaufficiale.it/atto/serie_generale/caricaArticolo?art.codiceRedazionale=22A00804&art.dataPubblicazioneGazzetta=2022-02-08&art.flagTipoArticolo=0&art.idArticolo=2&art.idGruppo=0&art.idSottoArticolo=1&art.idSottoArticolo1=10&art.progressivo=0&art.versione=1) | Official gazette legal record |
| **IT_5** | Harness supply chain and actions across sectors to ensure coherence with health | Legislative Decree No. 27/2021 concerning conversion into national legislation of the provisions of Regulation (EU) 2017/625 pursuant to article 12, letters a), b), c), d) and e) of Law No. 117/2019. | 2021 | Other (decree) | The decree outlines methods for official controls to ensure food safety, animal, and plant health, and specifies how national laws must align with European regulations. | To align Italy's regulations with EU standards on food safety and agriculture. | All food products | Not specified |  | [Normattiva: Decreto legislativo 2 febbraio 2021, n. 27](https://www.normattiva.it/uri-res/N2Ls?urn%3Anir%3Astato%3Adecreto.legislativo%3A2021-02-02%3B27=); [Istituto Superiore di Sanità PDF: D.Lgs. 27/2021](https://www.iss.it/documents/20126/5606021/Dlgs%2B27%2B2021.pdf/f2a0e495-2049-e510-7396-e7fb090599bf?t=1619505447383) | Official national legal database; public institute legal PDF |
| **IT_6** | Harness supply chain and actions across sectors to ensure coherence with health | Law 1 April 2022, No. 30 Provisions promoting local small agri-food productions. | 2022 | Law | This law sets rules for production methods, quality standards, labeling, and marketing of locally sourced agri-food products, with a focus on small-scale producers. | To encourage the production, distribution, and consumption of locally sourced agri-food products from small-scale producers. | Locally sourced agri-food products, | Not specified |  | [Normattiva: Legge 1 aprile 2022, n. 30](https://www.normattiva.it/uri-res/N2Ls?urn%3Anir%3Astato%3Alegge%3A2022%3B30=) | Official national legal database |
| **IT_7** | Harness supply chain and actions across sectors to ensure coherence with health | Law 9 March 2022, No. 23 Provisions for the protection, development and competitiveness of agricultural, agri-food and aquaculture production with organic methods. | 2022 | Law | This law establishes standards for organic production, processing, labeling, and certification, ensuring transparency and traceability to promote market access and consumer confidence in organic products. | To protect, develop, and enhance the competitiveness of organic agricultural, agri-food, and aquaculture production. | Organic food | Not specified |  | [Normattiva: Legge 9 marzo 2022, n. 23](https://www.normattiva.it/uri-res/N2Ls?urn%3Anir%3Astato%3Alegge%3A2022-03-09%3B23=); [Normattiva article view: Legge 9 marzo 2022, n. 23](https://www.normattiva.it/uri-res/N2Ls?urn%3Anir%3Astato%3Alegge%3A2022%3B23~art13=) | Official national legal database |
| **IT_8** | Use economic tools to address food affordability and purchase incentives | Legge di Bilancio 2024 | 2023 | Law | This law introduces a tax on foods with high sugar content (≥100g), aiming to discourage consumption of sugary products and address public health concerns such as obesity and diabetes. | To reduce health issues related to high sugar intake by creating financial disincentives for sugary food purchases. | All food products | Sugar |  | [Normattiva: Legge 30 dicembre 2023, n. 213](https://www.normattiva.it/uri-res/N2Ls?urn%3Anir%3Astato%3Alegge%3A2023-12-30%3B213=); [Normattiva detail: Bilancio di previsione dello Stato 2024](https://www.normattiva.it/atto/caricaDettaglioAtto?atto.codiceRedazionale=23G00223&atto.dataPubblicazioneGazzetta=2023-12-30&bloccoAggiornamentoBreadCrumb=true&classica=true&dataVigenza=22%2F01%2F2024&generaTabId=true&qId=&tabID=&tipoDettaglio=singolavigenza&title=lbl.dettaglioAtto) | Official national legal database |
| **LV_1** | Offer healthy food and set standards in public institutions and other specific settings | The Food Circulation Supervision Law | 2006 | Law | This policy sets nutritional standards for students in educational institutions, clients in long-term social care and rehabilitation facilities, and patients in medical institutions. | To ensure the provision of healthy and balanced diets that meet the specific dietary needs of these groups. | School food products | Nutrients | X | [State Language Centre: Law on the Supervision of the Handling of Food](https://www.vvc.gov.lv/en/laws-and-regulations-republic-latvia-english/law-supervision-handling-food-amendments-07052020); [WIPO Lex: Law on the Supervision of the Handling of Food](https://www.wipo.int/wipolex/en/legislation/details/21790) | Official State Language Centre translation; WIPO legal record |
| **LV_2** | Harness supply chain and actions across sectors to ensure coherence with health | Cabinet Regulation No. 104 of 2010 on Procedures for the Recognition and Registration of Food Establishments | 2010 | Other (regulation) | The Cabinet Regulation outlines the steps and requirements for recognizing and registering food establishments, ensuring compliance with EU regulations on food of animal origin and general hygiene. | To ensure food establishments in Latvia comply with EU hygiene standards and regulations. | All food products | Not specified |  | [State Language Centre: Cab. Reg. No. 104](https://www.vvc.gov.lv/en/laws-and-regulations-republic-latvia-english/cab-reg-no-104-procedures-recognition-and-registration-food-establishments-amendments-03052022); [FAOLEX PDF: Cabinet Regulation No. 104](https://faolex.fao.org/docs/pdf/lat206600.pdf) | Official State Language Centre regulation page; FAOLEX direct PDF |
| **LV_3** | Improve the nutritional quality of the food supply | Regulations on Food-Grade Salt | 2015 | Other (regulation) | The regulations govern the distribution and use of iodized and non-iodized salt in food production in Latvia, specifying iodizing and fluoridating agents, as well as labeling requirements for food salt. | To establish mandatory safety, quality, classification, and labeling requirements for food-grade salt. | Food salt | Iodized and non-iodized salt, iodizing and fluoridating agents. |  | [State Language Centre: Cab. Reg. No. 696 - Regulations Regarding Edible Salt](https://www.vvc.gov.lv/en/laws-and-regulations-republic-latvia-english/cab-reg-no-696-regulations-regarding-edible-salt); [FAOLEX PDF: Regulations Regarding Edible Salt](https://faolex.fao.org/docs/pdf/lat206621.pdf) | Official State Language Centre regulation page; FAOLEX direct PDF |
| **LV_4** | Improve the nutritional quality of the food supply | Regulations No. 461 of the Cabinet of Ministers: Requirements for Food Quality Schemes, Procedures for Their Implementation, Operation, Monitoring, and Control. | 2016 | Other (regulation) | The regulations limit added salt to 1.8 grams per 100 grams in beef, pork, goat, sheep, and poultry meat products, and 1.25 grams per 100 grams in bread. | To limit the content of salt in some animal based foodstuffs. | Beef, pork, goat, sheep, poultry meat products and bread | Salt |  | [State Language Centre: Cab. Reg. No. 461](https://www.vvc.gov.lv/en/laws-and-regulations-republic-latvia-english/cab-reg-no-461-requirements-food-quality-schemes-procedures-implementation-operation-monitoring-and-control-thereof-amendments-11102016); [FAOLEX PDF: Requirements of the National Food Quality Scheme](https://faolex.fao.org/docs/pdf/lat178462.pdf) | Official State Language Centre regulation page; FAOLEX direct PDF |
| **LV_5** | Improve the nutritional quality of the food supply | Latvian national legislation on Trans Fatty Acids (TFAs) | 2018 | Law | This law sets a maximum trans fatty acid content of 2 g per 100 g of total fat in food products. Exceptions include products with less than 3% total fat, where the limit is 10 g per 100 g, and products with 3% to 20% total fat, with a limit of 4 g per 100 g. | To limit trans fatty acids in food products and improve overall public health by reducing harmful fats in the food supply. | All food products | Trans fatty acid |  | [State Language Centre: Cab. Reg. No. 301 - Maximum Permitted Amount of Trans Fatty Acids](https://www.vvc.gov.lv/en/laws-and-regulations-republic-latvia-english/cab-reg-no-301-regulations-regarding-maximum-permitted-amount-trans-fatty-acids-food-products); [FAOLEX PDF: Regulations Regarding Maximum Permitted Amount of Trans Fatty Acids](https://faolex.fao.org/docs/pdf/lat178459.pdf) | Official State Language Centre regulation page; FAOLEX direct PDF |
| **LV_6** | Harness supply chain and actions across sectors to ensure coherence with health | Cabinet Regulation No. 73 of 2019 on Requirements for the Handling of Small Quantities of Raw Cow and Goat Milk | 2019 | Other (regulation) | The Cabinet Regulation sets hygiene standards and permit procedures for selling limited quantities of raw cow and goat milk. | To set hygiene standards and permit procedures for the direct sale of raw cow and goat milk. | Raw cow and goat milk | Not specified |  | [FAOLEX PDF: Cabinet Regulation No. 73](https://faolex.fao.org/docs/pdf/lat206629.pdf); [European Commission/Latvia summary PDF: Raw cow and goat milk handling requirements](https://www.em.gov.lv/sites/em/files/media_file/pcp-zm-agri-mk73-en_0.pdf) | FAOLEX direct PDF; official ministry/Commission regulatory summary PDF |
| **LV_7** | Nutrition label standards and regulations on the use of claims and implied claims on food | Cabinet Regulation No. 514 of 2020 on Requirements for the Food Distribution after Expiry of the Date of Minimum Durability | 2020 | Other (regulation) | The regulation outlines procedures for distributing packaged food labeled "Best before..." after its expiry date, excluding infant food. | To provide guidelines for the safe distribution of expired, consumable food to reduce waste. | Packaged food | Not specified |  | [State Language Centre: Cab. Reg. No. 514](https://www.vvc.gov.lv/en/laws-and-regulations-republic-latvia-english/mk-noteikumi-nr-514-prasibas-partikas-izplatisanai-pec-minimala-deriguma-termina-beigam-ar-grozijumiem-lidz-28032023); [FAOLEX PDF: Requirements for the Food Distribution after Expiry of the Date of Minimum Durability](https://faolex.fao.org/docs/pdf/lat206646.pdf) | Official State Language Centre regulation page; FAOLEX direct PDF |
| **LT_1** | Harness supply chain and actions across sectors to ensure coherence with health | Law on the Consumer Protection (No. I-657) | 1994 | Law | This law defines consumer rights, establishes consumer protection authority frameworks, regulates consumer awareness and interactions with providers, and outlines out-of-court protection procedures and liability for violations. | To ensure the safety of consumers. | All food products | Not specified |  | [FAOLEX direct PDF: Republic of Lithuania Law on Consumer Protection No. I-657](https://faolex.fao.org/docs/pdf/lit26418_1994.pdf) | FAOLEX direct legal-text PDF |
| **LT_2** | Set incentives and rules to create a healthy retail and food service environment | Regulation on preschool and school nutrition | 2012 | Other (regulation) | This regulation aims to promote healthy nutrition in preschools and schools by increasing the consumption of fresh produce, reducing sugary and salty snacks, and combating obesity. Progress is tracked through dietary and health indicators. | To support healthy nutrition for children and encourage healthier eating habits. | School food products | Nutrients | X | [Official e-Seimas record: Order No. V-964 on organising children’s nutrition](https://e-seimas.lrs.lt/portal/legalAct/lt/TAD/TAIS.411986) | Official national legal database |
| **LT_3** | Nutrition label standards and regulations on the use of claims and implied claims on food | Order on the Labelling of Foodstuffs by Keyhole Symbol | 2014 | Other (ministerial order) | This order introduces voluntary Keyhole symbol labeling for pre-packaged foods with lower fat, sugar, and salt content, and higher dietary fiber. It excludes unpackaged foods, children's foods under 36 months, and large-scale catering. Producers must notify the Ministry of Health about Keyhole-labeled products. | To promote healthier eating habits by helping consumers identify foods with better nutritional profiles through clear labeling. | Pre-packaged foods | Fat, fiber, sugar, and salt | X | [Lithuanian Ministry of Health PDF: Order on the Labelling of Foodstuffs by “Keyhole” Symbol](https://sam.lrv.lt/uploads/sam/documents/files/Veiklos_sritys/visuomenes-sveikatos-prieziura/mityba-ir-fizinis-aktyvumas/rakto-skylute/Keyhole%20LT%20EN%20%28notif%29.pdf) | Official ministry legal/policy PDF |
| **LT_4** | Offer healthy food and set standards in public institutions and other specific settings | Order of Regarding the Approval of the Description of the Procedure for Organizing Children's Nutrition | 2017 | Other (ministerial order) | This order establishes guidelines for organizing meal services in schools and child care facilities, focusing on ensuring food quality and safety. It promotes a balanced diet by setting recommendations on appropriate foods and restricting unhealthy products for children. | To promote a healthy, safe diet that meets children's nutritional needs and encourages healthy eating habits. | School food products | Not specified | X | [Official e-Seimas record: 2018 Order No. V-394 amending the children’s nutrition procedure](https://e-seimas.lrs.lt/portal/legalAct/lt/TAD/558922c146d111e88151f16ae94c33e2); [Lithuanian Ministry of Health page on nutrition and physical activity, citing Order No. V-394](https://sam.lrv.lt/lt/veiklos-sritys/visuomenes-sveikatos-prieziura/mityba-ir-fizinis-aktyvumas-2/mityba) | Official national legal database; official ministry policy page |
| **LT_5** | Improve the nutritional quality of the food supply | Lithuanian order establishing maximum limits for fats | 2019 | Other (ministerial order) | The recommendations aim to meet dietary recommendations which urge to limit the intake of trans fatty acids (TFA). | To reduce the intake of harmful trans fatty acids. | All food products | Trans Fatty Acids |  | [Infolex legal text: Order No. V-1202 on maximum permitted trans fatty acid content in food products](https://www.infolex.lt/ta/436453); [Official e-TAR record: Order No. V-165 repealing Order No. V-1202 following EU trans-fat rules](https://www.e-tar.lt/portal/fr/legalAct/5ed05630608911eb9dc7b575f08e8bea) | Legal database text; official Register of Legal Acts record |
| **LT_6** | Improve the nutritional quality of the food supply | Lithuanian order establishing maximum limits for fats | 2019 | Other (ministerial order) | The order introduces a maximum limit of 2 gram of trans fatty acids per 100 gram of the total fat content. For foods with a fat content of less than 3%, the maximum limit is will be 10 g per 100g of the total fat content | To reduce the intake of harmful trans fatty acids. | All food products | Trans Fatty Acids |  | [Infolex legal text: Order No. V-1202 on maximum permitted trans fatty acid content in food products](https://www.infolex.lt/ta/436453); [Official e-TAR record: Order No. V-165 repealing Order No. V-1202](https://www.e-tar.lt/portal/fr/legalAct/5ed05630608911eb9dc7b575f08e8bea) | Legal database text; official Register of Legal Acts record |
| **LU_1** | Nutrition label standards and regulations on the use of claims and implied claims on food | Grand-Ducal Regulation of November 20, 1993, Concerning Infant Formulae and Follow-on Formulae | 1993 | Other (regulation) | This regulation sets detailed standards for the composition, labeling, and marketing of infant and follow-on formulae, including nutrient content, additives, contamination limits, and microbiological criteria. It also regulates promotional practices and advertising. | To set standards for the composition and labeling of infant formulae and follow-on formulae, ensuring compliance with the International Code of Marketing of Breast-milk Substitutes. | Infant food products | Nutrients, additives and contaminnants | X | [Legilux: Règlement grand-ducal du 20 novembre 1993 concernant les préparations pour nourrissons et les préparations de suite](https://legilux.public.lu/eli/etat/leg/rgd/1993/11/20/n3/jo) | Official national legal database |
| **LU_2** | Give nutrition education and skills | Action Plan for the Promotion of Healthy Eating and Physical Activity | 2006 | Policy | The policy promotes healthier eating and physical activity by integrating nutrition into education, reducing sugary drink consumption, improving school meals with fresh foods, and supporting local health initiatives. | To raise awareness about the importance of healthy lifestyles, emphasizing nutrition, physical activity, and tackling obesity, especially in children, adolescents, and vulnerable groups. | All food products | Nutrients | X | [Government of Luxembourg PDF: Plan d’action “Gesond iessen – méi bewegen”](https://gouvernement.lu/dam-assets/fr/actualites/articles/2006/07/05gesond_iessen/Dosser_Plan_action_sante.pdf); [GIMB public portal: 2006 action plan page](https://gimb.public.lu/fr/publications/2006/Plan-GIMB-2006.html) | Official government policy PDF/page |
| **LU_3** | Nutrition label standards and regulations on the use of claims and implied claims on food | Règlement grand-ducal du 7 mai 2021 relatif à l’utilisation du logo Nutri-Score | 2021 | Other (regulation) | This regulation governs the use of the Nutri-Score logo, providing guidelines on its design, application, and mandatory use by registered food operators. The Nutri-Score is a five-level scale that classifies food products based on their nutritional quality to help consumers make healthier choices. | To promote informed consumer decisions regarding nutritional quality by standardizing the use of the Nutri-Score logo on food products. | All food products | Nutrients |  | [Legilux: Règlement grand-ducal du 7 mai 2021 relatif à l’utilisation du logo Nutri-Score](https://legilux.public.lu/eli/etat/leg/rgd/2021/05/07/a396/jo); [Government of Luxembourg notice on publication of the Nutri-Score regulation](https://gouvernement.lu/en/actualites/agenda.gouvernement2024%2Ben%2Bactualites%2Btoutes_actualites%2Bcommuniques%2B2021%2B05-mai%2B27-reglement-nutriscore.html) | Official national legal database; official government notice |
| **LU_4** | Harness supply chain and actions across sectors to ensure coherence with health | Règlement grand-ducal du 24 décembre 2021. | 2021 | Other (regulation) | This regulation imposes fees on businesses following market inspections that reveal non-compliance with food safety or materials regulations. It covers how fees are calculated and recovered and includes provisions for appealing penalties. | To enforce compliance with food safety standards by applying financial penalties after non-compliant findings during inspections. | All food products | Not specified |  | [Legilux: Règlement grand-ducal du 24 décembre 2021 instituant la perception de taxes for food-market official controls](https://legilux.public.lu/eli/etat/leg/rgd/2021/12/24/a35/jo); [ECOLEX record: Règlement grand-ducal du 24 décembre 2021](https://www.ecolex.org/fr/details/legislation/reglement-grand-ducal-du-24-decembre-2021-instituant-la-perception-de-taxes-dans-le-cadre-des-operations-de-controle-du-marche-des-denrees-alimentaires-et-de-materiaux-et-objets-destines-a-entrer-en-contact-avec-des-denrees-alimentaires-devenues-necessaires-a-la-suite-dun-premier-controle-ayant-releve-des-manquements-aux-dispositions-europeennes-legales-ou-reglementaires-lex-faoc207875/) | Official national legal database; ECOLEX specific record |
| **LU_5** | Harness supply chain and actions across sectors to ensure coherence with health | Law of April 26, 2022, concerning official inspections of agricultural products. | 2022 | Law | The law sets rules for official controls and activities to ensure compliance with food law, animal feed regulations, and standards for animal health, welfare, plant health, and plant protection products. | To Implement effective official controls to comply with EU standards and safeguard consumer health. | Animal and plant food products | Not specified |  | [Legilux: Loi du 26 avril 2022 relative aux contrôles officiels des produits agricoles](https://legilux.public.lu/eli/etat/leg/loi/2022/04/26/a204/jo) | Official national legal database |
| **MT_1** | Harness supply chain and actions across sectors to ensure coherence with health | Fruit and Vegetables Marketing Standards Regulations, 2015 (L.N. 109 of 2005). | 2005 | Other (regulation) | This regulation sets marketing standards for fruit and vegetables, ensuring quality control and consistency in the sale and distribution of these products in accordance with national and EU guidelines. | To maintain food security by regulating the marketing standards of fruit and vegetables. | Fruit and vegetables | Not specified |  | [Legislation Malta PDF: Fruit and Vegetables Marketing Standards Regulations, S.L. 117.36](https://legislation.mt/eli/sl/117.36/eng/pdf); [ECOLEX record: Fruit and Vegetables Marketing Standards Regulations, 2015 (L.N. 109 of 2005)](https://www.ecolex.org/details/legislation/fruit-and-vegetables-marketing-standards-regulations-2015-ln-109-of-2005-lex-faoc144155/) | Official national legal database PDF; ECOLEX specific record |
| **MT_2** | Nutrition label standards and regulations on the use of claims and implied claims on food | Infant Formulae and Follow-on Formulae 2007, LN 304 of 2007 | 2007 | Other (regulation) | This regulation sets compositional and labeling standards for infant and follow-on formulae, covering purity criteria, maximum residue limits for pesticides, and other substances | To establish compositional and labeling standards for infant formulae and follow-on formulae to ensure their safety and suitability. | Infant food products | Not specified | X | [Legislation Malta: Infant Formulae and Follow-on Formulae Regulations, S.L. 449.52](https://legislation.mt/eli/sl/449.52/eng); [FAOLEX direct PDF: L.N. 304 of 2007 Infant Formulae and Follow-on Formulae Regulations](https://faolex.fao.org/docs/pdf/mlt75219.pdf) | Official national legal database; FAOLEX direct legal-text PDF |
| **MT_3** | Offer healthy food and set standards in public institutions and other specific settings | Healthy Eating Lifestyle Plan (HELP) | 2007 | Policy | The plan fosters a supportive school environment for healthy habits through a flexible curriculum, nutrition guidelines, tuck shop inspections, and monitoring of students' dietary and physical health. | To integrate comprehensive health education into Maltese schools, providing the necessary frameworks and resources to support students in adopting and maintaining healthier lifestyles. | School food products | Not specified | X | [Official HELP PDF: Healthy Eating Lifestyle Plan](https://www.hesc.org.mt/wp-content/uploads/2016/06/healty-eating-lifestyle-plan.pdf); [HELP resources PDF](https://www.hesc.org.mt/wp-content/uploads/2016/06/healty-eating-lifestyle-plan-resources.pdf) | Official education/health policy PDF |
| **MT_4** | Give nutrition education and skills | Whole School Approach to Healthy Lifestyle: Healthy Eating and Physical Activity Policy | 2015 | Policy | This policy promotes holistic education in healthy eating and physical activity, encouraging balanced lifestyles through a flexible curriculum and consistent health messaging in schools to empower students to make informed choices. | To integrate healthy eating and physical activity into education, fostering a supportive school environment that promotes lifelong healthy habits. | School food products | Not specified | X | [Official policy PDF: Whole School Approach to a Healthy Lifestyle: Healthy Eating and Physical Activity Policy](https://sustainabledevelopment.gov.mt/wp-content/uploads/2024/10/Whole-School-Approach-to-a-Healthy-Lifestyle-Healthy-Eating-and-Physical-Activity-Policy.pdf); [HESC policy copy: Whole School Approach to Healthy Living](https://www.hesc.org.mt/wp-content/uploads/2023/10/Annex-IV-A-Whole-School-Approach-to-Healthy-Living-Healthy-Eating-and-Physical-Activity-Policy.pdf) | Official government policy PDF; official education/health policy PDF |
| **MT_5** | Harness supply chain and actions across sectors to ensure coherence with health | Food and Nutrition Policy and Action Plan for Malta 2015-2020 | 2015 | Policy | This policy promotes healthy nutrition by boosting fruit and vegetable intake, reducing unhealthy foods, enhancing accessibility, and supporting water access and fiscal incentives for healthier choices. | Promote healthy diets in Malta, integrate health into food policies, address health inequalities, and focus on obesity prevention, particularly for children. | All food products | Nutrients | X | [Ministry of Health PDF: Food and Nutrition Policy and Action Plan for Malta 2015-2020](https://health.gov.mt/wp-content/uploads/2023/04/Food_and_Nutrition_Policy_and_Action_Plan_for_Malta_2015-2020_EN.pdf) | Official government policy PDF |
| **MT_6** | Offer healthy food and set standards in public institutions and other specific settings | Subsidiary Legislation 550.01: Procurement of Food for Schools Regulations, under the Healthy Lifestyle Promotion and Care of Non-Communicable Diseases Act | 2018 | Other (regulation) | This legislation requires that only food meeting the criteria set by the Advisory Council can be provided or sold in schools. It enforces a healthy eating program, prohibits the advertising or sponsorship of non-compliant foods, and mandates access to drinking water for students. | To ensure schools promote healthy eating by adhering to set standards and restricting non-compliant food promotions. | School food products | Not specified | X | [Legislation Malta PDF: Procurement of Food for Schools Regulations, S.L. 550.01](https://legislation.mt/eli/sl/550.1/eng/pdf); [Legislation Malta: Legal Notice 266 of 2018 Procurement of Food for Schools Regulations](https://legislation.mt/DownloadDocument.aspx?app=lp&itemid=29226&l=1); [Superintendence of Public Health page listing S.L. 550.01](https://superintendencepublichealth.gov.mt/en/strategy-development-and-implementation-unit/legislation/) | Official national legal database PDF; official public health legislation page |
| **MT_7** | Harness supply chain and actions across sectors to ensure coherence with health | Malta’s National Action Plan for Sustainable Use of Pesticides 2019 – 2023. | 2019 | Policy | The Plan aims to establish management strategies to reduce pesticide risks on food and promote alternative methods to lessen dependency on pesticide use. | To establish an efficient tool to support the sustainable use of pesticides in Malta. | All food products | Pesticides |  | [Official PDF: Malta’s National Action Plan for Sustainable Use of Pesticides 2019-2023](https://sustainabledevelopment.gov.mt/wp-content/uploads/2024/10/Maltas-National-Action-Plan-for-Sustainable-Use-of-Pesticides-2019-2023.pdf); [MCCAA notice: revised National Action Plan for Sustainable Use of Pesticides 2019-2023](https://mccaa.org.mt/Section/Content?contentId=3866) | Official government policy PDF; official authority notice |
| **MT_8** | Nutrition label standards and regulations on the use of claims and implied claims on food | Production and Sale of Dairy and Traditional Dairy Products Rules, 2022. (L.N. 321 of 2022). | 2022 | Other (regulation) | This regulation set a general prohibition of sale of dairy products unless they are produced and labelled as required and in accordance with their rules and from establishments approved by the Department and registered with the Commission. | To regulate the production and labeling of dairy products, ensuring food safety and compliance with established standards. | Dairy products | Not specified |  | [Legislation Malta: Production and Sale of Dairy and Traditional Dairy Products Rules, S.L. 437.110](https://legislation.mt/eli/sl/437.110/eng); [FAOLEX direct PDF: Production and Sale of Dairy and Traditional Dairy Products Rules, 2022](https://faolex.fao.org/docs/pdf/mlt213781.pdf); [ECOLEX record: Production and Sale of Dairy and Traditional Dairy Products Rules, 2022](https://www.ecolex.org/details/legislation/production-and-sale-of-dairy-and-traditional-dairy-products-rules-2022-ln-321-of-2022-lex-faoc213781/) | Official national legal database; FAOLEX direct PDF; ECOLEX specific record |
| **MD_1** | Nutrition label standards and regulations on the use of claims and implied claims on food | Law n. 78 of 18-03-2004 regarding food products | 2004 | Law | The policy regulates food production, processing, and distribution to ensure safety and contamination standards for all food items, including fortified and special nutritional foods, excluding raw materials for personal use. | To establish a comprehensive legal framework for the safety, quality, and fair trade of food products, protect consumer interests, and promote equitable practices in the food industry. | All food products | Not specified |  | [FAOLEX record: Law No. 78-XV on foodstuffs](https://www.fao.org/faolex/results/details/en/c/LEX-FAOC054769) | FAOLEX specific legal record |
| **MD_2** | Harness supply chain and actions across sectors to ensure coherence with health | Law No. 78 –XV on foodstuffs | 2004 | Law | This law establishes the legal framework for the manufacturing, processing, and distribution of foodstuffs, regulating conditions for their circulation and ensuring overall safety. | To protect population’s health, consumers’ interests on foodstuffs, and to promote an equitable practice in the foodstuff trade. | All food products | Not specified |  | [FAOLEX record: Law No. 78-XV on foodstuffs](https://www.fao.org/faolex/results/details/en/c/LEX-FAOC054769) | FAOLEX specific legal record |
| **MD_3** | Harness supply chain and actions across sectors to ensure coherence with health | Government Decree No. HG221/2009 validating the Regulation regarding microbiological criteria for food products. | 2009 | Other (decree) | This Decree establishes microbiological criteria for food products and mandates food operators to adhere to hygiene protocols, including HACCP principles, to prevent contamination during production and distribution. | To safeguard public health by ensuring food safety through stringent microbiological control, especially in ready-to-eat foods, to prevent pathogens like Listeria monocytogenes. | All food products | Contaminants |  | [FAOLEX record: Government Decree No. HG221/2009 on microbiological criteria for food products](https://www.fao.org/faolex/results/details/en/c/LEX-FAOC223557) | FAOLEX specific legal record |
| **MD_4** | Harness supply chain and actions across sectors to ensure coherence with health | Governmental Decree No. 298 Validating Veterinary and Sanitary Requirement establishing measures for the control and supervision of certain substances and their residues in live animals and their products, as well as residues of veterinary medicines in products of animal origin. | 2011 | Other (decree) | This Decree outlines veterinary and sanitary measures for controlling and monitoring specific substances and their residues in live animals and animal products. It mandates regular inspection of veterinary medications and substances to ensure food safety and compliance with established standards for animal-derived products. | To ensure the safety of food products of animal origin by monitoring residues of veterinary medicines and other substances. | Animal-origin products | Not specified |  | [FAOLEX record: Governmental Decree No. 298 on veterinary residues in live animals and animal-origin products](https://www.fao.org/faolex/results/details/en/c/LEX-FAOC209207) | FAOLEX specific legal record |
| **MD_5** | Harness supply chain and actions across sectors to ensure coherence with health | Governmental Decree No. HG113/2021 validating the List of foodstuffs originating from the short food chain. replaced by Decision 180/2012. | 2012 | Other (decree) | This Decree approves the list of foodstuffs from the short food chain, ensuring that local products occupy at least 50% of the shelf space in retail stores. This promotes the availability and visibility of locally sourced food products, supporting small-scale producers and enhancing food security. | To increase the presence of locally sourced food in retail networks and support local agriculture. | Locally sourced food products | Not specified |  | [FAOLEX record: Governmental Decree No. HG113/2021 on foodstuffs from the short food chain](https://www.fao.org/faolex/results/details/en/c/LEX-FAOC204883) | FAOLEX specific legal record |
| **MD_6** | Offer healthy food and set standards in public institutions and other specific settings | National Programme in the field of food and nutrition 2014-2020 | 2014 | Policy | The program aims to enhance nutrition through an advisory council, updated dietary guidelines, breastfeeding promotion, healthy environments, and reforms to reduce saturated fats, sugars, and sodium. | To reduce illness and death from non-communicable diseases linked to poor diet and nutrient deficiencies. | All food products | Nutrients |  | [Legis.md record: Government Decision No. 730/2014 approving the National Programme in food and nutrition 2014-2020](https://www.legis.md/cautare/getResults?doc_id=103083&lang=ro) | Official Moldovan legal text database record |
| **MD_7** | Offer healthy food and set standards in public institutions and other specific settings | Order Nr. 622 21-05-2018 Healthy diet and adequate physical activity in educational institutions | 2018 | Other (ministerial order) | The order aims to exclude unhealthy foods high in sugar, fat, salt, and calories from children's diets, promoting healthier eating habits to support their overall health and well-being. | To promote healthier eating habits supporting children's overall health and well-being. | School food products | Sugar, fat, salt, | X | [Legis.md record: Order No. 622/2018 amending healthy diet and physical activity recommendations for educational institutions](https://www.legis.md/cautare/getResults?doc_id=111307&lang=ro) | Official Moldovan legal text database record |
| **MD_8** | Nutrition label standards and regulations on the use of claims and implied claims on food | Law No. 279 “On information of consumers about foodstuffs | 2019 | Law | This law requires food labels in Moldova to display key nutritional information, including fats, carbohydrates, sugars, proteins, and salt, excluding dietary supplements and natural mineral waters. | To provide consumers in Moldova with essential nutritional information through clear labeling, including ingredient lists and nutritional declarations, to promote informed choices and support public health. | All food products | Nutrients |  | [FAOLEX record: Law No. 279 on information of consumers about foodstuffs](https://www.fao.org/faolex/results/details/en/c/LEX-FAOC183760) | FAOLEX specific legal record |
| **MD_9** | Nutrition label standards and regulations on the use of claims and implied claims on food | Governmental Decree No. HG624/2020 validating the Requirements of meat products. | 2020 | Other (decree) | This Decree establishes standards for meat products, covering quality, labeling, packaging, and transportation for both domestic and imported products. Exemptions include personal-use farm products, catering products, and items with under 5% meat content. The decree also specifies classification by processing type and meat content, requiring producers to use safety and quality management systems. | To ensure consumer safety and meat quality through standardized practices in production, labeling, and transportation. | Meat products. | Not specified |  | [FAOLEX record: Governmental Decree No. HG624/2020 on requirements for meat products](https://www.fao.org/faolex/results/details/en/c/LEX-FAOC204748) | FAOLEX specific legal record |
| **MD_10** | Harness supply chain and actions across sectors to ensure coherence with health | Governmental Decree No. 8 validating Veterinary and Sanitary Regulation setting requirements for animal health and public health when importing and supplying to the market certain products of animal origin. | 2020 | Other (decree) | This Decree establishes veterinary and sanitary requirements to ensure animal health and public safety when importing and marketing specific products of animal origin. The decree outlines specific rules for different products, such as meat and animal-derived items, ensuring hygiene and safety compliance upon entry to the market. | To ensure compliance with animal health and public safety standards for imported and marketed animal-origin products. | Animal-origin products | Not specified |  | [FAOLEX record: Governmental Decree No. 8 on import and market supply of certain animal-origin products](https://www.fao.org/faolex/results/details/en/c/LEX-FAOC196596) | FAOLEX specific legal record |
| **MD_11** | Nutrition label standards and regulations on the use of claims and implied claims on food | Governmental Decree No. HG483/2020 validating General Procedure of registration of food additives, food enzymes and food flavorings. | 2020 | Other (decree) | This Decree sets forth a structured procedure for registering, assessing, and authorizing food additives, enzymes, and flavorings. This ensures these substances meet safety and compliance standards before their use in food products. | To maintain food safety by regulating the use of food additives, enzymes, and flavorings in accordance with national and international standards. | All food products | Additives |  | [FAOLEX record: Governmental Decree No. HG483/2020 on registration of food additives, enzymes and flavourings](https://www.fao.org/faolex/results/details/en/c/LEX-FAOC204891) | FAOLEX specific legal record |
| **MD_12** | Harness supply chain and actions across sectors to ensure coherence with health | Governmental Decree No. HG671/2020 validating the Sanitary Regulation on extraction solvents used in the manufacturing of foodstuffs and food ingredients. | 2020 | Other (decree) | This Decree regulates the use of extraction solvents in manufacturing foodstuffs and ingredients, including imported solvents, with specific exclusions for additives and export-only products. | To ensure safe and regulated use of extraction solvents in food manufacturing. | All food products | Extraction solvents |  | [FAOLEX record: Governmental Decree No. HG671/2020 on extraction solvents](https://www.fao.org/faolex/results/details/en/c/LEX-FAOC204749) | FAOLEX specific legal record |
| **MD_13** | Harness supply chain and actions across sectors to ensure coherence with health | Governmental Decree No. HG815/2020 validating Requirements for quality of apiculture products, including beeswax, propolis, royal jelly and pollen, intended for consumption by the population. | 2020 | Other (decree) | This Decree establish criteria for production, collection, processing, packaging, storage, transportation, and marketing of beekeeping products, both domestically produced and imported. | Government Decree No. HG815/2020 establishes standards for the quality of apiculture products such as beeswax, propolis, royal jelly, and pollen, ensuring their suitability for consumption by the public. | All food products | Not specified |  | [FAOLEX record: Governmental Decree No. HG815/2020 on quality of apiculture products](https://www.fao.org/faolex/results/details/en/c/LEX-FAOC204698) | FAOLEX specific legal record |
| **MD_14** | Harness supply chain and actions across sectors to ensure coherence with health | Order No. OMSMPS1003/2020 of the Ministry of Public Health, Labour and Social Protection "On state registration of food additives." da Nr 109 a Nr 116 ordini facenti parte di un regolamento nazionale sugli additivi alimentari (no documento ufficiale) | 2020 | Other (ministerial order) | This Order authorizes the registration and use of the food additive "Max Multiwitamina Colfarm" in Moldova, produced by Zakłady Farmaceutyczne Colfarm S.A., Poland. | To officially register and regulate the use of the "Max Multiwitamina Colfarm" food additive within Moldova, ensuring compliance with safety standards. | All food products | Additives |  | [FAOLEX record: Order No. OMSMPS1003/2020 on state registration of food additives](https://www.fao.org/faolex/results/details/en/c/LEX-FAOC204740) | FAOLEX specific legal record |
| **MD_15** | Harness supply chain and actions across sectors to ensure coherence with health | Order No. OMSMPS1004/2020 of the Ministry of Public Health, Labour and Social Protection "On state registration of food additives." | 2020 | Other (ministerial order) | This Order registers the food additives "Noflat" and "Flora Protect Baby" by Gricar Chemical S.R.L., Italy, for use in Moldova, adding them to the official list of registered food additives. | To authorize and regulate the use of specific food additives within Moldova, ensuring compliance with national health standards. | Infant food products | Additives | X | [FAOLEX record: Order No. OMSMPS1004/2020 on state registration of food additives](https://www.fao.org/faolex/results/details/en/c/LEX-FAOC204741) | FAOLEX specific legal record |
| **MD_16** | Harness supply chain and actions across sectors to ensure coherence with health | Order No. OMSMPS1005/2020 of the Ministry of Public Health, Labour and Social Protection "On state registration of food additives." | 2020 | Other (ministerial order) | This Order authorizes and registers several food supplements, including Propoli plus EPID, Spirulina, Vitavit Magnesium & Potassium, Ginkgo Plus, Pausa Night & Day, and Serenotte Plus, manufactured by Specchiasol S.R.L., Italy. | To ensure the safe use and compliance of registered food supplements within Moldova by maintaining a regulated list. | dietary supplements | Not specified |  | [FAOLEX record: Order No. OMSMPS1005/2020 on state registration of food additives](https://www.fao.org/faolex/results/details/en/c/LEX-FAOC204744) | FAOLEX specific legal record |
| **MD_17** | Harness supply chain and actions across sectors to ensure coherence with health | Order No. OMSMPS1006/2020 of the Ministry of Public Health, Labour and Social Protection "On state registration of food additives." | 2020 | Other (ministerial order) | This Order authorizes the registration of several food supplements, including Phytalgic Myoconfort, Cystiregul Plus, Skinsublim Hyaluronic, among others, manufactured by Laboratoires Nutreov Physcience, France. | To regulate the use and ensure safety compliance of listed food supplements within Moldova by maintaining a government-approved registry. | dietary supplements | Not specified |  | [FAOLEX record: Order No. OMSMPS1006/2020 on state registration of food additives](https://www.fao.org/faolex/results/details/en/c/LEX-FAOC204745) | FAOLEX specific legal record |
| **MD_18** | Harness supply chain and actions across sectors to ensure coherence with health | Order No. OMSMPS1008/2020 of the Ministry of Public Health, Labour and Social Protection "On state registration of food additives." | 2020 | Other (ministerial order) | This Order registers and authorizes the use of the food additive "Sage Forte with licorice and menthol" by Bioleki OOO, Belarus, in Moldova. The product is added to the official registry of approved food additives. | To ensure safety and regulatory compliance for Sage Forte with licorice and menthol within Moldova’s food industry. | All food products | Additives |  | [FAOLEX record: Order No. OMSMPS1008/2020 on state registration of food additives](https://www.fao.org/faolex/results/details/en/c/LEX-FAOC204711) | FAOLEX specific legal record |
| **MD_19** | Harness supply chain and actions across sectors to ensure coherence with health | Order No. OMSMPS1009/2020 of the Ministry of Public Health, Labour and Social Protection "On state registration of food additives." | 2020 | Other (ministerial order) | This Order authorizes the use of food additives "Sirop de Cimbrișor-Farmaco" and "Silymar" produced by O.M. Farmaco S.A., Moldova. These products are included in the national registry of approved food additives. | To regulate and ensure the safe use of registered food additives in Moldova. | All food products | Additives |  | [FAOLEX record: Order No. OMSMPS1009/2020 on state registration of food additives](https://www.fao.org/faolex/results/details/en/c/LEX-FAOC204708) | FAOLEX specific legal record |
| **MD_20** | Harness supply chain and actions across sectors to ensure coherence with health | Order No. OMSMPS1010/2020 of the Ministry of Public Health, Labour and Social Protection "On state registration of food additives." | 2020 | Other (ministerial order) | This Order authorizes the use of the food additives "Soluro Duo" (Latvia) and "Enhydria" (Italy) within Moldova. These products are officially included in the national registry of approved food additives. | To ensure safe use and proper registration of food additives (Soluro Duo) for consumer protection. | All food products | Additives |  | [FAOLEX record: Order No. OMSMPS1010/2020 on state registration of food additives](https://www.fao.org/faolex/results/details/en/c/LEX-FAOC204712) | FAOLEX specific legal record |
| **MD_21** | Harness supply chain and actions across sectors to ensure coherence with health | Order No. OMSMPS1011/2020 of the Ministry of Public Health, Labour and Social Protection "On state registration of food additives." | 2020 | Other (decree) | This Order authorizes the registration and use of the food additive "Spirulina 500 mg," produced by SC Alevia S.R.L., Romania, within Moldova. This decree ensures the additive is included in the official registry for approved use in food products. | To formalize the use of Spirulina 500 mg as a registered food additive to maintain safety and regulatory compliance. | All food products | Additives |  | [FAOLEX record: Order No. OMSMPS1011/2020 on state registration of food additives](https://www.fao.org/faolex/results/details/en/c/LEX-FAOC204710) | FAOLEX specific legal record |
| **MD_22** | Harness supply chain and actions across sectors to ensure coherence with health | Order No. OMSMPS1012/2020 of the Ministry of Public Health, Labour and Social Protection "On state registration of food additives." | 2020 | Other (decree) | This decree authorizes the food additives Wermiplant, Apivit, and Calmoplant by Depofarm S.R.L. in Moldova. | To authorize and register the food additives Wermiplant, Apivit, and Calmoplant for use in the Republic of Moldova | All food products | Additives |  | [FAOLEX record: Order No. OMSMPS1012/2020 on state registration of food additives](https://www.fao.org/faolex/results/details/en/c/LEX-FAOC204707) | FAOLEX specific legal record |
| **MD_23** | Harness supply chain and actions across sectors to ensure coherence with health | Order No. OMSMPS1013/2020 of the Ministry of Public Health, Labour and Social Protection "On state registration of food additives." | 2020 | Other (decree) | This decree authorizes and registers the food additives Vita Grapes capsule and Eurosept by Eurofarmaco S.A. in Moldova | To authorize and register the food additives Vita Grapes capsule and Eurosept for use in the Republic of Moldova. | All food products | Additives |  | [FAOLEX record: Order No. OMSMPS1013/2020 on state registration of food additives](https://www.fao.org/faolex/results/details/en/c/LEX-FAOC204709) | FAOLEX specific legal record |
| **MD_24** | Harness supply chain and actions across sectors to ensure coherence with health | Order No. OMSMPS401/2020 of the Ministry of Public Health, Labour and Social Protection "On state registration of food additives." | 2020 | Other (decree) | This decree registers and authorizes the food additives Leni Complex, Lenimyr, Ferrogreen Plus (tablets and syrup), and Serenote in Moldova, ensuring their safety and quality. | To authorize and ensure the state registration of specific food additives for use in Moldova. | All food products | Additives |  | [FAOLEX record: Order No. OMSMPS401/2020 on state registration of food additives](https://www.fao.org/faolex/results/details/en/c/LEX-FAOC204882) | FAOLEX specific legal record |
| **MD_25** | Harness supply chain and actions across sectors to ensure coherence with health | Order No. OMSMPS419/2020 of the Ministry of Public Health, Labour and Social Protection "On state registration of food additives." | 2020 | Other (decree) | This decree registers and authorizes B!tonic Roza Spirit, Calmogen Plant Complex, and Ascovit Imunitate Syrup in Moldova, ensuring their safety and quality. | To authorize and ensure the state registration of specific food additives for use in Moldova. | All food products | Not specified |  | [FAOLEX record: Order No. OMSMPS419/2020 on state registration of food additives](https://www.fao.org/faolex/results/details/en/c/LEX-FAOC204881) | FAOLEX specific legal record |
| **MD_26** | Harness supply chain and actions across sectors to ensure coherence with health | Order No. OMSMPS731/2020 of the Ministry of Public Health, Labour and Social Protection "On state registration of food additives." | 2020 | Other (decree) | The decree registers and authorizes the food additive Enzylit in Moldova, ensuring it meets safety and quality standards. | To authorize and ensure the state registration of the food additive Enzylit for use in Moldova. | All food products | Additives |  | [FAOLEX record: Order No. OMSMPS731/2020 on state registration of food additives](https://www.fao.org/faolex/results/details/en/c/LEX-FAOC204868) | FAOLEX specific legal record |
| **MD_27** | Harness supply chain and actions across sectors to ensure coherence with health | Order No. OMSMPS732/2020 of the Ministry of Public Health, Labour and Social Protection "On state registration of food additives." | 2020 | Other (decree) | This decree registers and authorizes the food additive Fermentozim forte by Kvadrat-S LLC in Moldova. | To authorize and register the food additive Fermentozim forte for use in the Republic of Moldova. | All food products | Additives |  | [FAOLEX record: Order No. OMSMPS732/2020 on state registration of food additives](https://www.fao.org/faolex/results/details/en/c/LEX-FAOC204867) | FAOLEX specific legal record |
| **MD_28** | Harness supply chain and actions across sectors to ensure coherence with health | Order No. OMSMPS733/2020 of the Ministry of Public Health, Labour and Social Protection "On state registration of food additives." | 2020 | Other (decree) | This decree registers and authorizes the food additives Ferty Biotic Man and Ferty Vital by Fertypharm SL from Barcelona, Spain. | To authorize and register the food additives Ferty Biotic Man and Ferty Vital for use in the Republic of Moldova. | All food products | Additives |  | [FAOLEX record: Order No. OMSMPS733/2020 on state registration of food additives](https://www.fao.org/faolex/results/details/en/c/LEX-FAOC204869) | FAOLEX specific legal record |
| **MD_29** | Harness supply chain and actions across sectors to ensure coherence with health | Order No. OMSMPS734/2020 of the Ministry of Public Health, Labour and Social Protection "On state registration of food additives." | 2020 | Other (decree) | This decree registers and authorizes the food additive Gripalin by SC Alevia SRL from Fălticeni, Romania. | To authorize and register the food additive Gripalin for use in the Republic of Moldova | All food products | Additives |  | [FAOLEX record: Order No. OMSMPS734/2020 on state registration of food additives](https://www.fao.org/faolex/results/details/en/c/LEX-FAOC204873) | FAOLEX specific legal record |
| **MD_30** | Harness supply chain and actions across sectors to ensure coherence with health | Order No. OMSMPS735/2020 of the Ministry of Public Health, Labour and Social Protection "On state registration of food additives." | 2020 | Other (decree) | This decree registers and authorizes Ismi-Kid, Lacto-G kids, Omniphage, and Respiphage as food additives. | To authorize and register the food additives Ismi-Kid, Lacto-G kids, Omniphage, and Respiphage for use in the Republic of Moldova. | All food products | Additives |  | [FAOLEX record: Order No. OMSMPS735/2020 on state registration of food additives](https://www.fao.org/faolex/results/details/en/c/LEX-FAOC204872) | FAOLEX specific legal record |
| **MD_31** | Harness supply chain and actions across sectors to ensure coherence with health | Order No. OMSMPS736/2020 of the Ministry of Public Health, Labour and Social Protection "On state registration of food additives." | 2020 | Other (regulation) | This regulation establishes the registration and authorization process for Lactofiltrum® Eco by SA AVVA RUS in Kirov, Russia, including its listing as a registered food supplement. | To register and authorize the use of the food supplement Lactofiltrum® Eco in the Republic of Moldova. | dietary supplements | Not specified |  | [FAOLEX record: Order No. OMSMPS736/2020 on state registration of food additives](https://www.fao.org/faolex/results/details/en/c/LEX-FAOC204874) | FAOLEX specific legal record |
| **MD_32** | Harness supply chain and actions across sectors to ensure coherence with health | Order No. OMSMPS737/2020 of the Ministry of Public Health, Labour and Social Protection "On state registration of food additives." | 2020 | Other (regulation) | This regulation mandates the registration of food additives, including Ladiecs Care Essence, Fabenol, Forksoglii soft capsules, and others, in the List of Registered Food Supplements. | To establish state registration and authorization of specific food additives in the Republic of Moldova. | dietary supplements | Additives |  | [FAOLEX record: Order No. OMSMPS737/2020 on state registration of food additives](https://www.fao.org/faolex/results/details/en/c/LEX-FAOC204879) | FAOLEX specific legal record |
| **MD_33** | Harness supply chain and actions across sectors to ensure coherence with health | Order No. OMSMPS738/2020 of the Ministry of Public Health, Labour and Social Protection "On state registration of food additives." | 2020 | Other (regulation) | This regulation establishes the registration and authorization of food additives Levasil-70 and Levasil-140 by Micro Labs Limited from Karnataka, India, for use in Moldova. | To register and authorize specific food additives for use in the Republic of Moldova | All food products | Additives |  | [FAOLEX record: Order No. OMSMPS738/2020 on state registration of food additives](https://www.fao.org/faolex/results/details/en/c/LEX-FAOC204866) | FAOLEX specific legal record |
| **MD_34** | Harness supply chain and actions across sectors to ensure coherence with health | Order No. OMSMPS739/2020 of the Ministry of Public Health, Labour and Social Protection "On state registration of food additives." | 2020 | Other (ministerial order) | This order registers and authorizes the dietary supplement Lipoatrin forte by Eurofarmaco SA in Ialoveni, Moldova, including it in the List of registered dietary supplements. | To register and authorize the use of a dietary supplement in the Republic of Moldova. | dietary supplements | Not specified |  | [FAOLEX record: Order No. OMSMPS739/2020 on state registration of food additives](https://www.fao.org/faolex/results/details/en/c/LEX-FAOC204751) | FAOLEX specific legal record |
| **MD_35** | Harness supply chain and actions across sectors to ensure coherence with health | Order No. OMSMPS740/2020 of the Ministry of Public Health, Labour and Social Protection "On state registration of food additives." | 2020 | Other (ministerial order) | This order registers and authorizes NBL Probiotic Gold by Cell Biotech Co Ltd in Beylikduzu, Korea, in Moldova's list of registered food supplements. | To register and authorize the use of a food supplement in the Republic of Moldova. | dietary supplements | Not specified |  | [FAOLEX record: Order No. OMSMPS740/2020 on state registration of food additives](https://www.fao.org/faolex/results/details/en/c/LEX-FAOC204875) | FAOLEX specific legal record |
| **MD_36** | Harness supply chain and actions across sectors to ensure coherence with health | Order No. OMSMPS741/2020 of the Ministry of Public Health, Labour and Social Protection "On state registration of food additives." | 2020 | Other (ministerial order) | This order registers and authorizes the food additive Noflat by Gricar Chemical SRL in Brigherio, Italy, for use in Moldova, including it in the list of registered food additives. | To register and authorize the use of a food additive in the Republic of Moldova. | All food products | Additives |  | [FAOLEX record: Order No. OMSMPS741/2020 on state registration of food additives](https://www.fao.org/faolex/results/details/en/c/LEX-FAOC204863) | FAOLEX specific legal record |
| **MD_37** | Harness supply chain and actions across sectors to ensure coherence with health | Order No. OMSMPS742/2020 of the Ministry of Public Health, Labour and Social Protection "On state registration of food additives." | 2020 | Other (ministerial order) | This order registers and authorizes the dietary supplements Optivit Vit Shine and Ora Aid Shine oral sprays by Influx Healthcare in Moldova. | To register and authorize the use of specific dietary supplements in the Republic of Moldova. | dietary supplements | Not specified |  | [FAOLEX record: Order No. OMSMPS742/2020 on state registration of food additives](https://www.fao.org/faolex/results/details/en/c/LEX-FAOC204752) | FAOLEX specific legal record |
| **MD_38** | Harness supply chain and actions across sectors to ensure coherence with health | Order No. OMSMPS743/2020 of the Ministry of Public Health, Labour and Social Protection "On state registration of food additives." | 2020 | Other (ministerial order) | This order registers and authorizes the food supplement ProxSil Original by Eytelia Sprl in Moldova. | To register and authorize the use of the food supplement ProxSil Original in the Republic of Moldova. | dietary supplements | Not specified |  | [FAOLEX record: Order No. OMSMPS743/2020 on state registration of food additives](https://www.fao.org/faolex/results/details/en/c/LEX-FAOC204864) | FAOLEX specific legal record |
| **MD_39** | Harness supply chain and actions across sectors to ensure coherence with health | Order No. OMSMPS744/2020 of the Ministry of Public Health, Labour and Social Protection "On state registration of food additives." | 2020 | Other (ministerial order) | This order registers and authorizes the dietary supplement Sennadelax Plus by Farmaco SA in Chișinău, Moldova, for use in the country. | To register and authorize the use of the dietary supplement Sennadelax Plus in the Republic of Moldova | dietary supplements | Not specified |  | [FAOLEX record: Order No. OMSMPS744/2020 on state registration of food additives](https://www.fao.org/faolex/results/details/en/c/LEX-FAOC204754) | FAOLEX specific legal record |
| **MD_40** | Harness supply chain and actions across sectors to ensure coherence with health | Order No. OMSMPS745/2020 of the Ministry of Public Health, Labour and Social Protection "On state registration of food additives." | 2020 | Other (ministerial order) | This order registers and authorizes the food additive Azinc Pregnancy by Laboratoires Arkopharma in Carros Cedes, France, for use in Moldova. | To register and authorize the use of the food additive Azinc Pregnancy in the Republic of Moldova. | All food products | Additives |  | [FAOLEX record: Order No. OMSMPS745/2020 on state registration of food additives](https://www.fao.org/faolex/results/details/en/c/LEX-FAOC204876) | FAOLEX specific legal record |
| **MD_41** | Harness supply chain and actions across sectors to ensure coherence with health | Order No. OMSMPS746/2020 of the Ministry of Public Health, Labour and Social Protection "On state registration of food additives." | 2020 | Other (ministerial order) | This order mandates the registration of various food additives, including Syrup Sabelnik and Shark Cartilage, in Moldova's List of Registered Food Supplements. | To establish state registration requirements for specific food additives in Moldova. | dietary supplements | Additives |  | [FAOLEX record: Order No. OMSMPS746/2020 on state registration of food additives](https://www.fao.org/faolex/results/details/en/c/LEX-FAOC204880) | FAOLEX specific legal record |
| **MD_42** | Harness supply chain and actions across sectors to ensure coherence with health | Order No. OMSMPS747/2020 of the Ministry of Public Health, Labour and Social Protection "On state registration of food additives." | 2020 | Other (ministerial order) | This order registers and authorizes dietary supplements by SC Laboratoarele Fares Bio Vital SRL from Orăștie, Romania, ensuring safety and quality compliance. | To register and authorize specific dietary supplements in the Republic of Moldova. | dietary supplements | Not specified |  | [FAOLEX record: Order No. OMSMPS747/2020 on state registration of food additives](https://www.fao.org/faolex/results/details/en/c/LEX-FAOC204753) | FAOLEX specific legal record |
| **MD_43** | Harness supply chain and actions across sectors to ensure coherence with health | Order No. OMSMPS748/2020 of the Ministry of Public Health, Labour and Social Protection "On state registration of food additives." | 2020 | Other (ministerial order) | This order registers and authorizes the dietary supplement Digestiv by Balkan Pharmaceuticals SRL in Chișinău, Moldova | To register and authorize the dietary supplement Digestiv in the Republic of Moldova. | dietary supplements | Not specified |  | [FAOLEX record: Order No. OMSMPS748/2020 on state registration of food additives](https://www.fao.org/faolex/results/details/en/c/LEX-FAOC204755) | FAOLEX specific legal record |
| **MD_44** | Harness supply chain and actions across sectors to ensure coherence with health | Order No. OMSMPS749/2020 of the Ministry of Public Health, Labour and Social Protection "On state registration of food additives." | 2020 | Other (ministerial order) | This order registers and authorizes the dietary supplement Delapis manufactured by Pharmacy Laboratories SC in Warsaw | To register and authorize the dietary supplement Delapis in the Republic of Moldova. | dietary supplements | Not specified |  | [FAOLEX record: Order No. OMSMPS749/2020 on state registration of food additives](https://www.fao.org/faolex/results/details/en/c/LEX-FAOC204756) | FAOLEX specific legal record |
| **MD_45** | Harness supply chain and actions across sectors to ensure coherence with health | Order No. OMSMPS750/2020 of the Ministry of Public Health, Labour and Social Protection "On state registration of food additives." | 2020 | Other (ministerial order) | This order registers and authorizes the dietary supplement Biopure Max by Agettis Supplement Ltd in Limassol, Cyprus | To register and authorize the dietary supplement Biopure Max in the Republic of Moldova. | dietary supplements | Not specified |  | [FAOLEX record: Order No. OMSMPS750/2020 on state registration of food additives](https://www.fao.org/faolex/results/details/en/c/LEX-FAOC204757) | FAOLEX specific legal record |
| **MD_46** | Harness supply chain and actions across sectors to ensure coherence with health | Order No. OMSMPS751/2020 of the Ministry of Public Health, Labour and Social Protection "On state registration of food additives." | 2020 | Other (ministerial order) | This order registers several food additives, including Cotipsilium Kinetic and Ferrogreen Plus, as food supplements. | To establish the state registration of specific food additives in the Republic of Moldova | dietary supplements | Additives |  | [FAOLEX record: Order No. OMSMPS751/2020 on state registration of food additives](https://www.fao.org/faolex/results/details/en/c/LEX-FAOC204877) | FAOLEX specific legal record |
| **MD_47** | Harness supply chain and actions across sectors to ensure coherence with health | Order No. OMSMPS752/2020 of the Ministry of Public Health, Labour and Social Protection "On state registration of food additives." | 2020 | Other (ministerial order) | This order registers the dietary supplement Bonflor by SC Fiterman Pharma SRL (Romania) for use in Moldova to ensure compliance and safety. | To establish the state registration of the dietary supplement Bonflor in the Republic of Moldova. | dietary supplements | Not specified |  | [FAOLEX record: Order No. OMSMPS752/2020 on state registration of food additives](https://www.fao.org/faolex/results/details/en/c/LEX-FAOC204758) | FAOLEX specific legal record |
| **MD_48** | Harness supply chain and actions across sectors to ensure coherence with health | Order No. OMSMPS990/2020 of the Ministry of Public Health, Labour and Social Protection "On state registration of food additives." | 2020 | Other (decree) | This decree mandates the registration of the food additive Ammivit by O.C.S. Natur Bravo S.A. for use in Moldova, adding it to the registered food additives list for food safety compliance. | To establish the state registration of the food additive Ammivit in the Republic of Moldova. | All food products | Additives |  | [FAOLEX record: Order No. OMSMPS990/2020 on state registration of food additives](https://www.fao.org/faolex/results/details/en/c/LEX-FAOC204703) | FAOLEX specific legal record |
| **MD_49** | Harness supply chain and actions across sectors to ensure coherence with health | Order No. OMSMPS991/2020 of the Ministry of Public Health, Labour and Social Protection "On state registration of food additives." | 2020 | Other (decree) | This decree registers food additives from Pharmalife Research (Italy) and Thien Duoc/DHG Pharmaceuticals (Vietnam), including Appetito Concentrato Fluido, Biomelatonin, Isilax Mama, Immuno, Crila Forte, and Eyelight Ganat. | To establish the state registration of multiple food additives for authorized use in the Republic of Moldova. | All food products | Additives |  | [FAOLEX record: Order No. OMSMPS991/2020 on state registration of food additives](https://www.fao.org/faolex/results/details/en/c/LEX-FAOC204705) | FAOLEX specific legal record |
| **MD_50** | Harness supply chain and actions across sectors to ensure coherence with health | Order No. OMSMPS992/2020 of the Ministry of Public Health, Labour and Social Protection "On state registration of food additives." | 2020 | Other (decree) | This decree registers food additives BTY, MLS, ALT, and GRW from MP KSS S.R.L. in Chișinău, adding them to the List of Registered Food Additives for safety and quality | To establish the state registration and authorization of specific food additives enriched with vitamins for use in the Republic of Moldova. | All food products | Additives |  | [FAOLEX record: Order No. OMSMPS992/2020 on state registration of food additives](https://www.fao.org/faolex/results/details/en/c/LEX-FAOC204704) | FAOLEX specific legal record |
| **MD_51** | Harness supply chain and actions across sectors to ensure coherence with health | Order No. OMSMPS995/2020 of the Ministry of Public Health, Labour and Social Protection "On state registration of food additives." | 2020 | Other (ministerial order) | This order registers the food additive Digex Forte by Fiterman Pharma S.R.L. in Romania for use in Moldova, including it in the List of Registered Food Additives to ensure safety and quality. | To register and authorize the food additive Digex forte for use in the Republic of Moldova. | All food products | Additives |  | [FAOLEX record: Order No. OMSMPS995/2020 on state registration of food additives](https://www.fao.org/faolex/results/details/en/c/LEX-FAOC204699) | FAOLEX specific legal record |
| **MD_52** | Harness supply chain and actions across sectors to ensure coherence with health | Order No. OMSMPS996/2020 of the Ministry of Public Health, Labour and Social Protection "On state registration of food additives." | 2020 | Other (ministerial order) | This order registers food additives Eucalivit and Lamivit-T by O.M. Vitapharm Com S.R.L. in Moldova, including them in the List of Registered Food Additives to ensure safety and quality. | To register and authorize the food additives Eucalivit and Lamivit-T for use in the Republic of Moldova. | All food products | Additives |  | [FAOLEX record: Order No. OMSMPS996/2020 on state registration of food additives](https://www.fao.org/faolex/results/details/en/c/LEX-FAOC204701) | FAOLEX specific legal record |
| **MD_53** | Harness supply chain and actions across sectors to ensure coherence with health | Order No. OMSMPS997/2020 of the Ministry of Public Health, Labour and Social Protection "On state registration of food additives." | 2020 | Other (decree) | This decree registers and authorizes the food additive Fitolizyna Nefrocaps Plus from Polpharma S.A. for safety and quality compliance in Moldova. | To register and authorize the food additive Fitolizyna Nefrocaps Plus for use in the Republic of Moldova. | All food products | Additives |  | [FAOLEX record: Order No. OMSMPS997/2020 on state registration of food additives](https://www.fao.org/faolex/results/details/en/c/LEX-FAOC204739) | FAOLEX specific legal record |
| **MD_54** | Harness supply chain and actions across sectors to ensure coherence with health | Order No. OMSMPS998/2020 of the Ministry of Public Health, Labour and Social Protection "On state registration of food additives." | 2020 | Other (ministerial order) | This order regulates the registration of food supplements in Moldova, including Ginger, Turmeric & Bromelain; EchinEeze; Pure Collagen; and Apple Cider Vinegar from Les 3 Chenes. | To register and authorize the use of various food supplements in the Republic of Moldova. | dietary supplements | Not specified |  | [FAOLEX record: Order No. OMSMPS998/2020 on state registration of food additives](https://www.fao.org/faolex/results/details/en/c/LEX-FAOC204746) | FAOLEX specific legal record |
| **MD_55** | Harness supply chain and actions across sectors to ensure coherence with health | Order No. OMSMPS999/2020 of the Ministry of Public Health, Labour and Social Protection "On state registration of food additives." | 2020 | Other (decree) | This decree regulates the registration of food additives in Moldova, including Glucosamine 2000 from DHC Corporation, ensuring health and safety compliance. | To register and authorize the use of specific food additives in the Republic of Moldova. | All food products | Additives |  | [FAOLEX record: Order No. OMSMPS999/2020 on state registration of food additives](https://www.fao.org/faolex/results/details/en/c/LEX-FAOC204742) | FAOLEX specific legal record |
| **MD_56** | Harness supply chain and actions across sectors to ensure coherence with health | Governmental Decree No. 768 validating the National Program for the Development of Apiculture in the Republic of Moldova for 2021-2025 and the Action Plan for 2021-2022 for its implementation. | 2020 | Other (decree) | The program expands Moldovan apiculture internationally, enhances cooperation, improves resources for beekeepers, promotes honey branding, and addresses certification challenges to boost revenue and public health. | To enhance Moldovan apiculture competitiveness, promote sustainability, and improve honey quality and safety for public health and state revenue. | All food products | Not specified |  | [FAOLEX record: Governmental Decree No. 768 on the National Programme for Development of Apiculture 2021-2025](https://www.fao.org/faolex/results/details/en/c/LEX-FAOC204810) | FAOLEX specific policy/legal record |
| **MD_57** | Nutrition label standards and regulations on the use of claims and implied claims on food | Government Decision No. HG442/2023 amending Governmental Decree No. HG538/2009 validating Sanitary Regulation on food additives. | 2023 | Law | This law revises the definition and regulatory framework for food additives in Moldova. It outlines specific dosage forms, including capsules and powders, and establishes criteria for exclusion from the Register of Food Additives. Management of notifications, registrations, and safety assessments is handled by the National Agency for Public Health. | To improve food additive regulations, ensuring safety and compliance within the food sector. | All food products | Additives |  | [FAOLEX record: Government Decision No. HG442/2023 amending sanitary regulation on food additives](https://www.fao.org/faolex/results/details/en/c/LEX-FAOC224288) | FAOLEX specific legal record |
| **MD_58** | Harness supply chain and actions across sectors to ensure coherence with health | Government Decree No. HG14/2023 "On the organization and functioning of the National Agency on Food Safety." | 2023 | Other (decree) | This Decree establishes the National Agency on Food Safety in Moldova. The agency oversees food safety and quality, regulates the production of ethyl alcohol and alcoholic beverages, and ensures compliance with plant and animal health regulations. | To implement state policies for food safety and ensure comprehensive oversight in the food sector, including animal and plant health protection. | All food products | Not specified |  | [FAOLEX record: Government Decree No. HG14/2023 on the National Agency on Food Safety](https://www.fao.org/faolex/results/details/en/c/LEX-FAOC223064) | FAOLEX specific legal record |
| **MD_59** | Nutrition label standards and regulations on the use of claims and implied claims on food | Government Decree No. HG941/2023 amending Government Resolution No. 229/2013 validating Sanitary Regulations on Food Additives. | 2023 | Other (decree) | This Decree amends Government Resolution No. 229/2013 regarding food additives. It updates the lists of food additives in line with European regulations and allows pre-existing products to remain on the market until their expiration or stock depletion. | To align food additive regulations with European standards and ensure the continued availability of existing products until their natural market expiration. | All food products | Additives |  | [FAOLEX record: Government Decree No. HG941/2023 amending Government Resolution No. 229/2013 on food additives](https://www.fao.org/faolex/results/details/en/c/LEX-FAOC225157) | FAOLEX specific legal record |
| **MD_60** | Harness supply chain and actions across sectors to ensure coherence with health | Order No. OANSA149/2023 of the National Agency for Food Safety validating the Instruction on withdrawal and recall of foodstuffs, not corresponding to applicable in the food sector regulations, and on the notification of the National Agency for Food Safety. | 2023 | Other  (ministerial order) | This Order establishes protocols for the withdrawal and recall of non-compliant food products. It details actions for operators to manage unsafe items, including those with pathogens, chemical or physical hazards, or undeclared allergens. | To ensure consumer safety by guiding food operators on managing recalls of hazardous products through cooperation with the National Agency for Food Safety. | All food products | Not specified |  | [FAOLEX record: Order No. OANSA149/2023 on withdrawal and recall of non-compliant foodstuffs](https://www.fao.org/faolex/results/details/en/c/LEX-FAOC223130) | FAOLEX specific legal record |
| **MD_61** | Harness supply chain and actions across sectors to ensure coherence with health | Order No. OMAIA57/2023 of the Ministry of Agriculture and Food Industry validating control checklists of the National Food Safety Agency. | 2023 | Other (ministerial order) | This Order establishes control checklists for the National Food Safety Agency, ensuring regulatory compliance across various food industry sectors. | To standardize and validate food safety control processes to enhance oversight and regulatory compliance in the national food industry. | All food products | Not specified |  | [FAOLEX PDF: Order No. OMAIA57/2023 validating control checklists of the National Food Safety Agency](https://faolex.fao.org/docs/pdf/mol224940.pdf) | FAOLEX-hosted official legal PDF |
| **MD_62** | Nutrition label standards and regulations on the use of claims and implied claims on food | Law No. LP237/2023 "On ecological production and labelling of ecological products". | 2023 | Law | This law sets requirements for meal production and marketing, including temperature restrictions for oils and fats and microbiological standards for food safety. | Ensure sustainable ecological production, fair competition, proper market function, and consumer confidence in ecological products. | Edible oils and fats. | Fat |  | [FAOLEX PDF: Law No. LP237/2023 on ecological production and labelling of ecological products](https://faolex.fao.org/docs/pdf/mol220924.pdf) | FAOLEX-hosted official legal PDF |
| **ME_1** | Improve the nutritional quality of the food supply | Ordinance on the quality of cereals, mill and bakery products, pasta and quick-frozen dough | 2013 | Other(ordinance) | This ordinance sets limits on sodium hydroxide in bakery products and salt content in pasta to enhance food safety and ensure consistent product formulation, protecting consumer health. | To ensure safety and consistency in bakery and pasta products by regulating the allowable levels of specific chemicals and salt content. | Infant food products | Salt/sodium |  | [ECOLEX record: Regulation on methods of physical and chemical analysis on the quality of cereals, milling and bakery products, pasta and frozen dough](https://www.ecolex.org/details/legislation/regulation-on-methods-of-physical-and-chemical-analysis-on-the-quality-of-cereals-milling-and-bakery-products-pasta-and-frozen-dough-lex-faoc141650/) | ECOLEX/FAOLEX specific legal record |
| **ME_2** | Improve the nutritional quality of the food supply | Regulation on vitamins, minerals and other food additives. | 2016 | Other (regulation) | This regulation defines permissible food additives, specifies allowable vitamins and minerals, prohibits harmful substances, restricts trans fats, and sets limits to protect consumer health. | To establish standards and conditions for adding vitamins, minerals, and other substances to food products, ensuring they meet specific requirements for market placement. | All food products | Trans Fatty Acids |  | [ECOLEX record: Regulation on vitamins, minerals and other food additives](https://www.ecolex.org/details/legislation/regulation-on-vitamins-minerals-and-other-food-additives-lex-faoc168160/) | ECOLEX/FAOLEX specific legal record |
| **ME_3** | Set incentives and rules to create a healthy retail and food service environment | Action Plan for Nutrition 2017-2018 | 2017 | Policy | The program improves nutrition, strengthens healthcare, reduces salt intake, develops guidelines, promotes physical activity, regulates food advertising, and supports breastfeeding. | To create an environment that promotes proper nutrition, focusing on fostering conditions that encourage healthy eating habits across various settings and populations. | All food products | Salt/sodium |  | [Official Ministry of Health PDF: Programme of measures for improving nutrition status and diet with action plan](https://wapi.gov.me/download/8da8ccec-645a-42e1-ab50-f6609b33d4d2?version=1.0) | Official government policy PDF |
| **ME_4** | Harness supply chain and actions across sectors to ensure coherence with health | Regulation amending the Regulation on additives for animal feed and premixtures which can be placed on the market. | 2019 | Other (regulation) | This regulation specifies permitted animal food additives, ensuring products meet safety standards to protect animal health and promote safe livestock feeding practices. | To establish a standardized list of approved additives and premixtures for animal feed to ensure safety and compliance within the market. | All food products | Additives |  | [ECOLEX record: Regulation amending the Regulation on additives for animal feed and premixtures](https://www.ecolex.org/details/legislation/regulation-amending-the-regulation-on-additives-for-animal-feed-and-premixtures-which-can-be-placed-on-the-market-lex-faoc178779/) | ECOLEX/FAOLEX specific legal record |
| **ME_5** | Harness supply chain and actions across sectors to ensure coherence with health | General crisis plan for crisis management when food poses a risk to human health. | 2019 | Policy | This Plan details procedures for managing severe, uncontrollable risks in the food and feed chain, addressing major health threats to Montenegro and neighboring regions. | To establish a national crisis management framework for addressing food safety risks that threaten human health. | All food products | Not specified |  | [Official Government PDF: General crisis plan for crisis management when food poses a risk to human health](https://wapi.gov.me/download/f4d96cc0-3c2a-49f1-882f-1b99d250d242?version=1.0) | Official government policy PDF |
| **ME_6** | Harness supply chain and actions across sectors to ensure coherence with health | Regulation amending the Regulation on the manner of performing official controls on non-animal origin food and feed. | 2020 | Other (regulation) | This regulation updates the Rulebook for food and animal feed under enhanced control, detailing inspection frequency, product categories, origins, risks, and sampling percentages for safety. | To amend the Rulebook regarding the official control of food of non-animal origin and food for animals of non-animal origin, enhancing safety monitoring practices. | All food products | Not specified |  | [ECOLEX record: Regulation amending the Regulation on official controls on non-animal-origin food and feed](https://www.ecolex.org/details/legislation/regulation-amending-the-regulation-on-the-manner-of-performing-official-controls-on-non-animal-origin-food-and-feed-lex-faoc194933/) | ECOLEX/FAOLEX specific legal record |
| **ME_7** | Harness supply chain and actions across sectors to ensure coherence with health | Regulation on measures to determine the presence of genetically modified rice and rice products originating in China upon import. | 2020 | Other (regulation) | This regulation mandates lab reports and health certificates for rice shipments from China, confirming no genetically modified rice, excluding non-rice and personal use items. | To regulate the importation and market placement of rice and rice products from China, ensuring that they are free from genetically modified organisms (GMOs) through laboratory testing and certification. | All food products | Not specified |  | [ECOLEX record: Regulation on genetically modified rice and rice products originating in China upon import](https://www.ecolex.org/details/legislation/regulation-on-measures-to-determine-the-presence-of-genetically-modified-rice-and-rice-products-originating-in-china-upon-import-lex-faoc195082/) | ECOLEX/FAOLEX specific legal record |
| **ME_8** | Improve the nutritional quality of the food supply | Regulation on the minimum quality of salt for human consumption and food production. | 2020 | Other (regulation) | This regulation sets quality and labeling requirements for salt, ensuring it is primarily sodium chloride, and mandates labeling on sourcing, origin, granulation size, and iodization. | To prescribe the minimum quality standards for salt intended for human consumption and food production, along with the conditions for its market placement. | All food products | Salt/sodium |  | [ECOLEX record: Regulation on the minimum quality of salt for human consumption and food production](https://www.ecolex.org/details/legislation/regulation-on-the-minimum-quality-of-salt-for-human-consumption-and-food-production-lex-faoc195084/) | ECOLEX/FAOLEX specific legal record |
| **ME_9** | Harness supply chain and actions across sectors to ensure coherence with health | Order prohibiting the import and transit of consignments of poultry, birds and their products in order to prevent the introduction of highly pathogenic avian influenza. | 2021 | Other (ministerial order) | This Order bans the import and transport of poultry, birds, eggs, and related products from countries with suspected or confirmed highly pathogenic avian influenza to ensure public health and animal safety. | To prevent the entry and spread of highly pathogenic avian influenza in Montenegro through stringent import and transit regulations. | Poultry meat | Not specified |  | [ECOLEX record: Order prohibiting import and transit of poultry, birds and related products to prevent highly pathogenic avian influenza](https://www.ecolex.org/details/legislation/order-prohibiting-the-import-and-transit-of-consignments-of-poultry-birds-and-their-products-in-order-to-prevent-the-introduction-of-highly-pathogenic-avian-influenza-lex-faoc202479/) | ECOLEX/FAOLEX specific legal record |
| **ME_10** | Nutrition label standards and regulations on the use of claims and implied claims on food | Regulation amending the Regulation on new foods that can be used and placed on the market. | 2021 | Other (regulation) | This amendment updates novel food regulations, detailing usage, labeling, and protective measures to align Montenegrin law with EU standards for consumer safety | To amend the Regulation on new foods in Montenegro to ensure alignment with European Union standards and facilitate the safe market placement of novel foods. | All food products | Not specified |  | [ECOLEX record: Regulation amending the Regulation on new foods that can be used and placed on the market](https://www.ecolex.org/details/legislation/regulation-amending-the-regulation-on-new-foods-that-can-be-used-and-placed-on-the-market-lex-faoc206977/) | ECOLEX/FAOLEX specific legal record |
| **ME_11** | Harness supply chain and actions across sectors to ensure coherence with health | Food and feed safety measures Programme for 2021. | 2021 | Policy | The Programme mandates checks on live animals and products for illicit substances, contaminants, and genetically modified materials, including evaluations of pesticide residues and food safety standards. | To ensure the safety of food, feed, and materials that come into contact with them by monitoring residues of prohibited substances, veterinary drugs, and contaminants. | All food products | Contaminants |  | [ECOLEX record: Food and feed safety measures Programme for 2021](https://www.ecolex.org/details/legislation/food-and-feed-safety-measures-programme-for-2021-lex-faoc204531/) | ECOLEX/FAOLEX specific policy record |
| **ME_12** | Harness supply chain and actions across sectors to ensure coherence with health | Nitrate monitoring Programme in food of plant origin for 2021. | 2021 | Policy | This programme outlines responsibilities for monitoring and sampling nitrate levels in foods like spinach, lettuce, arugula, and infant cereals. | To ensure food safety by controlling and monitoring nitrate levels in specific plant-based foods, thereby protecting consumer health and complying with national and EU regulations. | Infant food products | Nitrates | X | [ECOLEX record: Nitrate monitoring Programme in food of plant origin for 2021](https://www.ecolex.org/details/legislation/nitrate-monitoring-programme-in-food-of-plant-origin-for-2021-lex-faoc204429/) | ECOLEX/FAOLEX specific policy record |
| **ME_13** | Harness supply chain and actions across sectors to ensure coherence with health | Pesticide residue monitoring Programme in food of plant and animal origin for 2022. | 2021 | Policy | The Programme sets guidelines for monitoring pesticide residues, detailing sampling priorities, laboratory tests, control methods, and sample handling. | To monitor pesticide residue levels in food of plant and animal origin in 2022, ensuring compliance with EU regulations. | All food products | Pesticides/residues |  | [ECOLEX record: Pesticide residue monitoring Programme in food of plant and animal origin for 2022](https://www.ecolex.org/details/legislation/pesticide-residue-monitoring-programme-in-food-of-plant-and-animal-origin-for-2022-lex-faoc210153/) | ECOLEX/FAOLEX specific policy record |
| **ME_14** | Harness supply chain and actions across sectors to ensure coherence with health | Regulation on conditions for import and export of food and feed in order to monitor the level of radioactivity due to the incident at the Chernobyl nuclear power plant. | 2022 | Other (regulation) | This regulation sets import and export standards for food and animal feed, monitoring cesium-137 contamination from Chernobyl to ensure safety and public health. | To ensure food safety by establishing maximum permissible levels of radioactivity in food and animal feed imported into or exported from Montenegro, specifically addressing cesium-137 contamination levels. | All food products | Not specified |  | [Official Gazette record: Regulation on import/export conditions for food and feed following the Chernobyl incident](https://www.sluzbenilist.me/propisi/32A34B1D-1148-4E35-B979-0980531807C9) | Official Montenegrin gazette legal record |
| **ME_15** | Use economic tools to address food affordability and purchase incentives | Law on Temporary Measures to Limit the Prices of Products of Special Importance for Human Life and Health | 2022 | Law | This law regulates temporary price controls on essential goods like food and hygiene items, excluding those covered by other laws, and allows price caps, reductions, and margin controls during disruptions. | To prevent market disruptions and eliminate the negative effects of price increases on essential products. | All food products | Not specified |  | [ECOLEX record: Law on temporary measures to limit prices of products of special importance for human life and health](https://www.ecolex.org/details/legislation/law-on-temporary-measures-to-limit-the-prices-of-products-of-special-importance-for-human-life-and-health-lex-faoc210083/) | ECOLEX/FAOLEX specific legal record |
| **ME_16** | Harness supply chain and actions across sectors to ensure coherence with health | Programme of safety and quality measures for food and feed for 2022. | 2022 | Policy | This program details procedures for hazard monitoring, sampling, and testing of food and feed, including responsibilities, funding, corrective measures, and non-compliance protocols. | To ensure food and feed safety by systematically monitoring contaminants, residues, and environmental hazards in food, feed, and materials that come into contact with them. | All food products | Not specified |  | [Official Gazette record: Programme of safety and quality measures for food and feed for 2022](https://www.sluzbenilist.me/propisi/292999) | Official Montenegrin gazette programme record |
| **ME_17** | Harness supply chain and actions across sectors to ensure coherence with health | Nitrate monitoring programme in food of plant origin for 2022. | 2022 | Policy | The program establishes 2022 nitrate monitoring tasks under Montenegrin food safety law to assess nitrate levels in leafy vegetables and processed cereals, protecting public health and addressing accumulation factors. | To assess and mitigate the health risks associated with nitrate consumption in plant-based foods. | All food products | Nitrates |  | [ECOLEX record: Nitrate monitoring programme in food of plant origin for 2022](https://www.ecolex.org/details/legislation/nitrate-monitoring-programme-in-food-of-plant-origin-for-2022-lex-faoc210079/) | ECOLEX/FAOLEX specific policy record |
| **ME_18** | Offer healthy food and set standards in public institutions and other specific settings | Programme for the improvement of the availability of food (fruits, vegetables, milk and dairy products) for schools. | 2022 | Policy | This policy enhances access to nutritious foods for primary school students, promoting health and local agriculture through educational engagement. Products must meet specific quality criteria, such as no added sugars, salts, fats, sweeteners, or artificial flavor enhancers. | To promote understanding of agriculture, healthy eating habits, and local food chains. | School food products | Salt/sodium | X | [ECOLEX record: Programme for the improvement of the availability of food for schools](https://www.ecolex.org/details/legislation/programme-for-the-improvement-of-the-availability-of-food-fruits-vegetables-milk-and-dairy-products-for-schools-lex-faoc210667/) | ECOLEX/FAOLEX specific policy record |
| **ME_19** | Harness supply chain and actions across sectors to ensure coherence with health | Decree on conditions, method and dynamics of implementation of agricultural policy measures for the year 2023-Agro-budget | 2023 | Other (decree) | This initiative allocates funds for agricultural and rural development, focusing on food and feed safety, and improving animal health and phytosanitary protections. | To enhance food security through targeted funding and support for various agricultural sectors. | All food products | Not specified |  | [ECOLEX record: Agro-budget decree for agricultural policy measures for 2023](https://www.ecolex.org/details/legislation/decree-on-conditions-method-and-dynamics-of-implementation-of-agricultural-policy-measures-for-the-year-2023-agro-budget-lex-faoc215256/) | ECOLEX/FAOLEX specific legal record |
| **NL_1** | Harness supply chain and actions across sectors to ensure coherence with health | "Warenwet" (Commodity Act) | 1953 | Law | This legislation establishes general rules and material-specific substance lists for food contact materials, including coatings and colorants, while promoting risk assessment practices for non-listed substances. | To regulate packaging and consumer articles that come into contact with food to ensure safety and compliance with health standards. | All food products | Not specified |  | [Wetten.nl record: Warenwet (Commodity Act)](https://wetten.overheid.nl/BWBR0001969) | Official Dutch legal database record |
| **NL_2** | Nutrition label standards and regulations on the use of claims and implied claims on food | Decree No. BWBR0005758 of 10 December 1992, on the Preparation and Treatment of Foodstuffs under the Commodities Act. | 1992 | Other (decree) | This decree establishes hygiene and quality rules for food and beverages, prohibiting non-compliant practices to ensure adherence to community standards. | To establish regulations for the preparation, processing, and handling of foodstuffs to ensure food safety and protect public health. | All food products | Not specified |  | [Wetten.nl record: Warenwetbesluit Bereiding en behandeling van levensmiddelen](https://wetten.overheid.nl/BWBR0005758) | Official Dutch legal database record |
| **NL_3** | Nutrition label standards and regulations on the use of claims and implied claims on food | Decree of June 4, 1998 | 1998 | Other (decree) | The decree outlines bread labeling requirements, specifying ingredients and standards for moisture, salt content, and the inclusion of bran or milk components, depending on the type of bread. | To establish labeling standards for bread, ensuring clarity on ingredients and composition. | Dairy products | Salt/sodium |  | [Wetten.nl record: Warenwetbesluit Meel en brood](https://wetten.overheid.nl/BWBR0009669) | Official Dutch legal database record |
| **NL_4** | Harness supply chain and actions across sectors to ensure coherence with health | Decree no. BWBR0018823 of 3 October 2005, establishing the Food Hygiene Commodities Act Decree | 2005 | Other (decree) | This decree restricts raw cow's milk trade for direct consumption, establishes hygiene codes, and sets food safety requirements for bulk transport of oils, fats, and sugar. | To promote food hygiene through the implementation of good hygiene practices and HACCP principles while regulating the trade of raw cow's milk intended for direct human consumption. | Dairy products | Sugar and fat |  | [Wetten.nl record: Warenwetbesluit hygiene van levensmiddelen](https://wetten.overheid.nl/BWBR0018823/) | Official Dutch legal database record |
| **NL_5** | Nutrition label standards and regulations on the use of claims and implied claims on food | The Choices Programme | 2006 | Policy | The Choices logo is a positive front-of-pack label for food and beverages, reflecting levels of saturated fats, trans fats, added sugars, salt, and fiber compared to similar products. | To promote healthier food choice and provide consumers with more information about nutritional content of the products. | All food products | Salt/sodium |  | [World Obesity/GINA policy action PDF: The Choices Programme](https://data.worldobesity.org/country/netherlands-153/actions.pdf) | Policy database-derived programme record |
| **NL_6** | Harness supply chain and actions across sectors to ensure coherence with health | Decree no. BWBR0026325 of 19 August 2009, containing rules in connection with Regulations (EC) no. 1332/2008, 1333/2008 and 1334/2008 (Commodities Act Decree on additives, flavorings and enzymes in foodstuffs). | 2009 | Other (decree) | This regulation establishes a framework for using food additives, enzymes, flavorings, and specific flavoring ingredients in food, aligning national practices with European standards. | To enhance food safety by ensuring that all permitted substances meet rigorous safety assessments and regulatory requirements. | All food products | Additives |  | [Wetten.nl record: Warenwetbesluit additieven, aromas en enzymen in levensmiddelen](https://wetten.overheid.nl/BWBR0026325) | Official Dutch legal database record |
| **NL_7** | Set incentives and rules to create a healthy retail and food service environment | Regulations on fat products | 2016 | Other (regulation) | This regulation prohibits the sale and preparation of fats not compliant with EU Regulation 1308/2013, limiting erucic acid to 5% and total erucic acid and isomers to 6.5%. | To regulate the composition and trade of certain fat and oil products to ensure compliance with specific standards set by EU regulations, thereby maintaining food quality and safety. | All food products | Fat |  | [Wetten.nl record: Warenwetbesluit Smeerbare vetproducten](https://wetten.overheid.nl/BWBR0010734) | Official Dutch legal database record |
| **NL_8** | Restrict food advertising and other forms of commercial promotion | Dutch Advertising Code for Food Products | 2019 | Policy | The Code bans unhealthy eating ads in children's media, restricts misleading health claims, and sets standards to protect children from exploitation and unhealthy lifestyles. | To ensure that all food advertisements are truthful, not misleading, and provide clear information about nutritional content. | All food products | Not specified | X | [Dutch Advertising Code: Food Products advertising code](https://www.reclamecode.nl/engels/dutch-advertising-code/special-advertising-codes/) | Self-regulatory advertising code / policy document |
| **NL_9** | Set incentives and rules to create a healthy retail and food service environment | National Product Improvement Approach: New criteria for product improvement | 2022 | Policy | The policy categorizes food products by composition, production methods, and shelf life, setting limits on salt, sugar, and saturated fat to promote healthier choices. | To make healthy food choices easier for Dutch citizens, addressing the high rate of overweight individuals through product improvement and informed choices. | All food products | Salt/sodium |  | [RIVM record: National Approach to Product Improvement](https://www.rivm.nl/en/food-and-nutrition/food-environment/national-approach-to-product-improvement) | Official public health institute policy page |
| **MK_1** | Improve the nutritional quality of the food supply | Regulation on the Quality of Food Salt | 1999 | Other (regulation) | This policy mandates food salt to contain at least 97% sodium chloride, less than 7% moisture, and be free from unauthorized impurities, with iodization at specific iodine levels and detailed labeling on origin, iodization, additives, and net weight. | To set requirements for the composition, iodization, and labeling of food salt. | All food products | Salt/sodium |  | [Official Gazette PDF: Rulebook on quality of food salt](https://slvesnik.com.mk/Issues/C85F8E52862347059E0491A99C7F70FD.pdf) | Official Gazette legal text PDF |
| **MK_2** | Restrict food advertising and other forms of commercial promotion | Law on safety of foodstuffs and products and materials coming into contact with foodstuffs | 2002 | Law | This policy restricts labeling and advertising of infant and young children's food that discourages breastfeeding, prohibiting manufacturers and traders from providing information that undermines it. | To regulate and restrict the marketing and labeling of infant and young child food products to protect breastfeeding and ensure accurate information. | Infant food products | Not specified | X | [WTO accession PDF: Law on safety of foodstuffs and contact materials](https://www.wto.org/english/thewto_e/acc_e/mkd_e/wtaccmkd24a3_leg_2.pdf) | WTO accession legal text PDF / official legal translation |
| **MK_3** | Harness supply chain and actions across sectors to ensure coherence with health | Law on organic farming | 2004 | Law | The law applies to agricultural products, processed foods, aquaculture products, seeds, seedlings, and yeasts used as food or feed. | To regulate the production, processing, and control of organic products in North Macedonia. | fishery and aquaculture products | Not specified |  | [ECOLEX/FAOLEX record: Law on organic farming](https://www.ecolex.org/details/legislation/law-on-organic-farming-lex-faoc152500/?q=23%2F2018&type=legislation) | ECOLEX/FAOLEX specific legal record |
| **MK_4** | Nutrition label standards and regulations on the use of claims and implied claims on food | Regulation on nutritional and health claims for commercial purposes in labeling, presentation and marketing of foods | 2005 | Other (regulation) | This policy aligns national rules with EU Regulation (EC) No. 1924/2006 to ensure nutrition and health claims on food products are clear, accurate, substantiated, and promote fair competition. | To align national regulations with EU Regulation (EC) No. 1924/2006 concerning nutrition and health claims made on food products. | All food products | Nutrients |  | [FVA PDF: Rulebook on nutrition and health claims](https://fva.gov.mk/images/2013_65-pravilnik_nutritivni_i_zdarvstveni_tvrdenja.pdf) | Official food authority legal text PDF |
| **MK_5** | Nutrition label standards and regulations on the use of claims and implied claims on food | Regulation on Food-Related Information | 2015 | Other (regulation) | This regulation updates national food labeling to align with EU Regulation (EU) No. 1169/2011, revising nutrition and health claims and replacing outdated rules for clearer, more consistent labeling across the EU. | The aim is to ensure clear, accurate, and consistent food labeling across the EU. | All food products | Nutrients |  | [FVA record: 2015-150 Rulebook on food-related information](https://fva.gov.mk/mk/zakon-bezbednost-hrana-pravilnici-odluki?page=1) | Official food authority legal text record |
| **MK_6** | Harness supply chain and actions across sectors to ensure coherence with health | Food and Nutrition action plan in the Republic of Macedonia | 2016 | Policy | This action plan aims to reduce premature death and diet-related diseases through a cross-sector approach focused on food system improvements and risk factor reduction. | To encourage healthy eating all citizens. | All food products | Not specified |  | [WHO NLiS/Gina PDF: North Macedonia nutrition policy profile](https://www.who.int/docs/default-source/nutritionlibrary/nlis-pdf-reports/nlis-profile-mkd.pdf?sfvrsn=76d3c95c_2) | WHO NLiS/GINA policy profile PDF |
| **PL_1** | Nutrition label standards and regulations on the use of claims and implied claims on food | Regulation on detailed requirements for commercial quality of honey. | 2003 | Other (regulation) | The regulation sets detailed requirements for honey's commercial quality, classifying it by origin, use, production method, and presentation, with specified physicochemical standards. | To establish clear standards for honey's quality, classification, and composition. | All food products | Not specified |  | [ELI record: Regulation on detailed requirements for commercial quality of honey](https://eli.gov.pl/eli/DU/2003/1773/ogl) | Official Polish ELI legal record |
| **PL_2** | Improve the nutritional quality of the food supply | Regulation on Food Fortification | 2003 | Other (regulation) | This regulation sets rules for added vitamins and minerals in foods, capping nutrient levels at 50% of the RDI per 100 g or serving, with allowances for Vitamin C and folate adjustments | To define the vitamins and minerals that must be added to specific foods and establish the minimum and maximum levels for these nutrients. | All food products | Nutrients |  | [ELI record: Regulation on substances enriching foods](https://eli.gov.pl/eli/DU/2003/237/ogl) | Official Polish ELI legal record |
| **PL_3** | Harness supply chain and actions across sectors to ensure coherence with health | Act on products of animal origin | 2005 | Law | The Act sets hygiene and control standards for animal-origin products, covering organ hygiene, market entry requirements, production standards, and official control methods for compliance. | To regulate various aspects related to the production, hygiene, and market introduction of products of animal origin, in alignment with EU standards. | Animal-origin products | Not specified |  | [ELI record: Act on products of animal origin](https://eli.gov.pl/eli/DU/2006/127/ogl) | Official Polish ELI legal record |
| **PL_4** | Harness supply chain and actions across sectors to ensure coherence with health | Act on safety of food and nutrition | 2006 | Law | The Act establishes hygiene and control standards for animal-origin products, detailing organ hygiene, market entry, production standards, and compliance controls | To ensure comprehensive food safety and nutrition by establishing clear sanitary and hygienic standards. | Animal-origin products | Not specified |  | [ELI record: Act on food and nutrition safety](https://eli.gov.pl/eli/DU/2006/1225/ogl) | Official Polish ELI legal record |
| **PL_5** | Nutrition label standards and regulations on the use of claims and implied claims on food | Regulation on the composition and labeling of dietary supplements | 2007 | Other (regulation) | The regulation outlines approved vitamins, minerals, and their chemical forms for dietary supplements, along with labeling requirements. | To ensure the safety, quality, and proper labeling of dietary supplements. | dietary supplements | Nutrients |  | [ELI record: Regulation on composition and labelling of dietary supplements](https://eli.gov.pl/eli/DU/2007/1425/ogl) | Official Polish ELI legal record |
| **PL_6** | Nutrition label standards and regulations on the use of claims and implied claims on food | Regulation on mushrooms authorized for marketing or production of mushroom preserves, foodstuffs containing mushrooms and qualifications of a mushroom classifier and mushroom expert. | 2008 | Other (regulation) | This regulation sets marketing and labeling standards for mushrooms to ensure accurate packaging and compliance with safety and quality standards for consumer protection. | To establish regulations for the marketing and labeling of mushrooms to ensure food safety. | All food products | Not specified |  | [ELI record: Regulation on mushrooms authorised for marketing/processing](https://eli.gov.pl/eli/DU/2008/1399/ogl) | Official Polish ELI legal record |
| **PL_7** | Harness supply chain and actions across sectors to ensure coherence with health | Regulation establishing veterinary requirements for the production of meat intended for the personal use | 2010 | Other (regulation) | This regulation enforces strict veterinary standards for meat production to safeguard public health and reduce foodborne illness risks while ensuring traceability. | To ensure the safety and quality of meat produced for personal use while maintaining animal health and welfare standards. | Meat products. | Not specified |  | [ISAP PDF: Regulation on veterinary requirements for meat for own use](https://isap.sejm.gov.pl/isap.nsf/download.xsp/WDU20102071370/O/D20101370.pdf) | Official Polish legal text PDF |
| **PL_8** | Harness supply chain and actions across sectors to ensure coherence with health | National Health Program for 2016 - 2020 | 2016 | Policy | The policy promotes breastfeeding, provides obesity support through nutritional counseling, and offers comprehensive healthcare services. | To improve public health by enhancing nutrition, physical activity, and promoting healthy aging. | All food products | Nutrients |  | [ISAP PDF: National Health Program 2016-2020](https://isap.sejm.gov.pl/isap.nsf/download.xsp/WDU20160001492/O/D20161492.pdf) | Official Polish legal text PDF |
| **PL_9** | Harness supply chain and actions across sectors to ensure coherence with health | Regulation on the running by the Agency for Restructuring and Modernization of Agriculture tasks related to the establishment of exceptional market support measures in the eggs and poultry meat sectors. | 2019 | Other (regulation) | The regulation outlines tasks for the Agency for Restructuring and Modernization of Agriculture (ARMA) in Poland to implement extraordinary market support measures in the egg and poultry meat sectors, as per EU Regulation 2018/1507. | To implement extraordinary market support measures for the egg and poultry meat sector. | Poultry meat | Not specified |  | [ECOLEX/FAOLEX record: Exceptional market support in eggs and poultry meat sectors](https://www.ecolex.org/details/legislation/regulation-on-the-running-by-the-agency-for-restructuring-and-modernization-of-agriculture-tasks-related-to-the-establishment-of-exceptional-market-support-measures-in-the-eggs-and-poultry-meat-sectors-lex-faoc192315/) | ECOLEX/FAOLEX specific legal record |
| **PL_10** | Nutrition label standards and regulations on the use of claims and implied claims on food | Act on Labeling Products as Free from Genetically Modified Organisms and the Use of Graphic Signs for Food and Animal Feed | 2019 | Law | The Act establishes labeling rules for food and feed, outlines obligations for marketing GMO-free products, compliance monitoring, liability for violations, and conditions for GMO-free labeling according to EU regulations | Establish clear labeling rules for food and feed, ensure compliance with GMO-free claims, and define obligations for marketing these products. | All food products | Not specified |  | [ELI record: Act on GMO-free product labelling](https://eli.gov.pl/eli/DU/2019/1401/ogl) | Official Polish ELI legal record |
| **PL_11** | Harness supply chain and actions across sectors to ensure coherence with health | Regulation on the collection of market data. | 2021 | Other (regulation) | The Regulation establishes a framework for collecting comprehensive market data in the agricultural sector, specifying data types, reporting entities, and reporting methods and deadlines. | To collect accurate market data on agricultural products. | All food products | Not specified |  | [ECOLEX/FAOLEX record: Regulation on the collection of market data](https://www.ecolex.org/fr/details/legislation/regulation-on-the-collection-of-market-data-lex-faoc206892/) | ECOLEX/FAOLEX specific legal record |
| **PT_1** | Improve the nutritional quality of the food supply | Law no. 75/2009 - Portugal Salt Reduction Program for Bread | 2009 | Law | This law sets a maximum of 1.4 g salt per 100 g of bread, exempting imported and traditional varieties, and mandates clear labeling of pre-packaged foods with visible salt content. | To reduce sodium intake among the population by decreasing salt levels in bread. | Bread/bakery products | Salt/sodium |  | [Diário da República: Law No. 75/2009 on salt reduction in bread](https://diariodarepublica.pt/dr/detalhe/lei/75-2009-493513) | Official Portuguese gazette legal record |
| **PT_2** | Use economic tools to address food affordability and purchase incentives | Value-Added Tax (VAT) Policy on Processed Foods | 2012 | Policy | Portugal imposes a value-added tax (VAT) on salty processed foods, while non-processed foods enjoy a reduced VAT rate. | To reduce salt in foodstuff. | All food products | Salt/sodium |  | [Diário da República: Law No. 64-A/2011, State Budget for 2012](https://diariodarepublica.pt/dr/detalhe/lei/64-a-2011-243768) | Official Portuguese gazette legal record |
| **PT_3** | Offer healthy food and set standards in public institutions and other specific settings | Order No. 7516-A/2016 limiting products harmful to health in vending machines | 2016 | Other (ministerial order) | Order No. 7516-A/2016 prohibits vending machines in Ministry of Health institutions/including health centers and hospitals/from selling high-fat, high-sugar, or high-salt items. | To promote public health, encouraging healthier eating habits and overall health improvement. | All food products | Salt/sodium |  | [Diário da República: Order No. 7516-A/2016 on vending machines](https://diariodarepublica.pt/dr/detalhe/despacho/7516-a-2016-74604818) | Official Portuguese gazette legal record |
| **PT_4** | Set incentives and rules to create a healthy retail and food service environment | National Program for the Promotion of Healthy Eating | 2017 | Policy | The initiative focuses on improving public health by reducing sugar and salt levels by 10%, limiting trans fats to under 2%, increasing fruit and vegetable consumption by 5%, raising awareness of the Mediterranean diet by 20%, and combating childhood obesity by 2020. | To improve public health by addressing dietary factors that contribute to chronic diseases and obesity. | All food products | Salt/sodium |  | [DGS/PNPAS PDF: National Program for the Promotion of Healthy Eating](https://comum.rcaap.pt/bitstreams/af1f6349-acd0-4fa7-9fce-da10b2c2b0e0/download) | Official public-health programme PDF |
| **PT_5** | Set incentives and rules to create a healthy retail and food service environment | Integrated Strategy for the Promotion of Healthy Eating | 2017 | Policy | The policy aims to monitor and limit trans-fat content in foods like cookies, pastries, and margarine while setting nutritional standards for salt and sugar to meet EU guidelines. | To enhance public health by increasing access to healthier food choices and reducing unhealthy options. | All food products | Salt/sodium |  | [Diário da República: Order No. 11418/2017 approving EIPAS](https://diariodarepublica.pt/dr/detalhe/despacho/11418-2017-114424591) | Official Portuguese gazette legal record |
| **PT_6** | Offer healthy food and set standards in public institutions and other specific settings | Portugal's 2018 Prohibition on High Fat, Sugar, and Salt Food Products in Government Health Institutions | 2018 | Other (decree) | Prohibits the sale of food products containing high levels of fat, sugar, and salt within all government health institutions. | To promote healthier dietary choices and improve public health in all government health institutions. | All food products | Salt/sodium |  | [Diário da República: Order No. 11391/2017 on food offer in health institutions](https://diariodarepublica.pt/dr/detalhe/despacho/11391-2017-114412574) | Official Portuguese gazette legal record |
| **PT_7** | Restrict food advertising and other forms of commercial promotion | Law No. 30/2019 on Advertising Food and Beverages High in Sugar, Fat, or Salt | 2019 | Law | This law prohibits advertising of foods and beverages high in sugar, fat, or salt during children’s TV programs, on children’s websites, and near schools. | To protect children from exposure to unhealthy food advertisements and promote healthier eating habits. | School food products | Salt/sodium | X | [Diário da República: Law No. 30/2019 on food advertising restrictions](https://diariodarepublica.pt/dr/detalhe/lei/30-2019-122151046) | Official Portuguese gazette legal record |
| **PT_8** | Nutrition label standards and regulations on the use of claims and implied claims on food | Law No. 3/2020 amending marketing rules for rice and broken rice intended for human consumption. | 2020 | Law | This law establishes standards for Oryza sativa L. and broken rice, clarifies labeling rules for specialty types, amends definitions for "Long" and "Extra" class rice, and updates amylose content determination standards. | To establish clear standards and labeling regulations for rice | All food products | Not specified |  | [Diário da República: Decree-Law No. 3/2020 on rice and broken rice marketing rules](https://diariodarepublica.pt/dr/detalhe/decreto-lei/3-2020-129113818) | Official Portuguese gazette legal record |
| **PT_9** | Harness supply chain and actions across sectors to ensure coherence with health | Resolution of the Council of Ministers No. 132/2021 approving the National Food and Nutrition Security Strategy. | 2021 | Policy | The Strategy promotes an integrated and sustainable food system to ensure the human right to adequate food, focusing on vulnerable populations and effective governance. | To ensure the realization of the human right to adequate food by promoting an integrated and sustainable food system that improves access to safe and nutritionally adequate food, particularly for vulnerable populations. | All food products | Not specified |  | [Diário da República: Resolution of the Council of Ministers No. 132/2021](https://diariodarepublica.pt/dr/detalhe/resolucao-conselho-ministros/132-2021-171183636) | Official Portuguese gazette policy/legal record |
| **RO_1** | Harness supply chain and actions across sectors to ensure coherence with health | Ministers' Order No. 387 on foods for special dietary uses | 2002 | Other (ministerial order) | The Order sets standards for foods with special nutritional purposes and mandates enforcement by responsible agencies. | To establish standards for foods with special nutritional purposes and ensure enforcement. | All food products | Nutrients |  | [Portal Legislativ: Order No. 387/251/2002 on foods for special dietary uses](https://legislatie.just.ro/Public/DetaliiDocument/103243) | Official Romanian legislative portal record |
| **RO_2** | Harness supply chain and actions across sectors to ensure coherence with health | Ministers’ Order nr. 1764 on foods for special dietary uses | 2007 | Other (ministerial order) | The policy regulates foods for special nutritional purposes, setting standards for infant formulas, labeling, pesticide limits, and restricting advertising to promote breastfeeding. | To regulate foods for special nutritional purposes, ensuring safety, proper labeling, and promoting breastfeeding. | Infant food products | Pesticides/residues | X | [Portal Legislativ: Order No. 1764/2007 amending special dietary-use food norms](https://legislatie.just.ro/Public/FormaPrintabila/00000G07AYX0B1ZKISM0JF5B36KLQSL8) | Official Romanian legislative portal printable legal text |
| **RO_3** | Offer healthy food and set standards in public institutions and other specific settings | Order nr. 1563 for approval List of non-recommended foods in schools | 2008 | Other(ordinance) | The ordinance restricts unhealthy food sales in schools, mandates nutritional labeling, hygiene compliance, and sets guidelines for daily nutrition, with enforcement by health authorities. | To restrict unhealthy food sales in schools and ensure proper nutrition and hygiene standards. | School food products | Nutrients | X | [Portal Legislativ: Order No. 1563/2008 on foods not recommended in schools](https://legislatie.just.ro/Public/FormaPrintabila/00000G2GXBJY4E7J5DB1W4CWDHSQPZMP) | Official Romanian legislative portal printable legal text |
| **RO_4** | Offer healthy food and set standards in public institutions and other specific settings | Legislation on prohibited food in schools (Law no. 123/2008) | 2008 | Law | Legislation sets maximum levels for sugar (15g), fat (20g), and salt (1.5g or 0.6g sodium) in food sold in schools. Soft drinks are banned, and drinking water must be accessible. | To prohibit some categories of foodstuffs in schools. | School food products | Salt/sodium | X | [Chamber of Deputies record: Law No. 123/2008 on healthy eating in pre-university education](https://www.cdep.ro/pls/legis/legis_pck.htp_act?ida=79764&pag=2) | Official parliamentary legal record |
| **RO_5** | Offer healthy food and set standards in public institutions and other specific settings | Ordinance 24/2010 for the Implementation of Fruit scheme in schools | 2010 | Other(ordinance) | The ordinance mandates free fruit distribution to students in grades I-VIII in Romanian state and accredited private schools, starting with apples for up to 100 school days. The Government will annually adjust the fruit type, frequency, and cost. | To provide free fruit to students and adjust the program annually. | School food products | Not specified | X | [Romanian Legislative Portal: Emergency Ordinance No. 24/2010 on the school fruit programme](https://legislatie.just.ro/Public/FormaPrintabila/00000G1PMIXPF0F2MEZ0ZPIPL80NZNPU) | Official national legislative text |
| **RO_6** | Harness supply chain and actions across sectors to ensure coherence with health | Order No. 152 for the safety of food of non-animal origin. | 2020 | Other (ministerial order) | The order ensures food safety through official controls on non-animal food products and ingredients during trade, with required accompanying documents. | To ensure food safety through official controls on non-animal food products during trade. | All food products | Not specified |  | [Romanian Legislative Portal: Order No. 152/2020 on food of non-animal origin](https://legislatie.just.ro/Public/DetaliiDocument/232219) | Official national legal database record |
| **RO_7** | Improve the nutritional quality of the food supply | Law 182/2020 | 2021 | Law | This law limits trans fats to 2g per 100g of fat and applies to both domestic and imported products. | To limit trans fatty acid content in  food. | All food products | Trans Fatty Acids |  | [Romanian Chamber of Deputies record: Law No. 182/2020 on trans fatty acids](https://www.cdep.ro/pls/legis/legis_pck.htp_act?ida=167419) | Official parliamentary legislation record |
| **RO_8** | Harness supply chain and actions across sectors to ensure coherence with health | Law No. 56 "On food additives." | 2021 | Law | The law establishes a framework for dietary supplements in Romania, detailing classification, regulatory oversight, market introduction, and covered substances like vitamins and minerals. | To protect consumer health, provide accurate information, and prevent unfair commercial practices. | dietary supplements | Nutrients |  | [FAOLEX PDF: Law No. 56/2021 on food supplements](https://faolex.fao.org/docs/pdf/rom205194.pdf) | FAOLEX hosted legal text PDF |
| **SK_1** | Set incentives and rules to create a healthy retail and food service environment | Food Act no. 152/1995 on foodstuffs | 1995 | Law | The Act regulates food production, processing, and marketing, setting obligations for food businesses to protect human health and consumer interests. | To ensure food safety, quality, and proper labeling, protecting public health and consumer rights. | All food products | Not specified |  | [FAOLEX record: Food Act No. 152/1995 on foodstuffs](https://www.fao.org/faolex/results/details/en/c/LEX-FAOC183196) | FAOLEX specific legal record |
| **SK_2** | Offer healthy food and set standards in public institutions and other specific settings | Decree of the Ministry of Education of the Slovak Republic of August 14, 2009 about school catering facilities | 2009 | Other (decree) | This policy sets standards for school catering, ensuring meal quality and nutrition, including staffing, food preparation, and quality control. | To establish standards for school catering that ensure high-quality, nutritious meals. | School food products | Nutrients | X | [JRC School Food Policy Factsheet: Slovakia](https://joint-research-centre.ec.europa.eu/system/files/2017-07/jrc-school-food-policy-factsheet-slovakia_en.pdf) | European Commission JRC policy factsheet |
| **SK_3** | Improve the nutritional quality of the food supply | Requirements for edible salt in foodstuffs | 2015 | Policy | The policy sets requirements for added salt in foods, excluding naturally occurring salt. | To regulate permissible levels of added salt in foods. | All food products | Salt/sodium |  | [ECOLEX record: Decree on spices, table salt, dehydrated meals and soup preparations](https://www.ecolex.org/details/legislation/decree-on-spices-table-salt-dehydrated-meals-and-soup-preparations-lex-faoc169011/) | ECOLEX/FAOLEX specific legal record |
| **SK_4** | Nutrition label standards and regulations on the use of claims and implied claims on food | Decree no. 83/2016 on meat products | 2016 | Other (decree) | This decree outlines the requirements for the production, handling, and marketing of meat products within Slovakia. | To outline requirements for the production, handling, labeling and marketing of meat products. | Meat products. | Not specified |  | [FAOLEX record: Decree No. 83/2016 on meat products](https://www.fao.org/faolex/results/details/en/c/LEX-FAOC169020) | FAOLEX specific legal record |
| **Sl_1** | Harness supply chain and actions across sectors to ensure coherence with health | Health Inspection Act | 1999 | Law | The law sets health inspection rules to protect public health in areas like food safety, infectious diseases, cosmetics, tobacco, and alcohol. | To protect public health through health inspections across various sectors. | All food products | Not specified |  | [FAOLEX record: Health Inspection Act](https://www.fao.org/faolex/results/details/en/c/LEX-FAOC211775) | FAOLEX specific legal record |
| **Sl_2** | Restrict food advertising and other forms of commercial promotion | Slovenian Code of Advertising Practice | 2009 | Policy | The policy prohibits food and beverage ads that undermine healthy living, make false health claims, or encourage overconsumption. | To regulate the advertising of food and beverages directed at children under the age of 16. | All food products | Not specified | X | [Slovenian Advertising Chamber PDF: Slovenski oglaševalski kodeks](https://www.soz.si/sites/default/files/soz_sok_slo.pdf) | Self-regulatory advertising code PDF |
| **Sl_3** | Improve the nutritional quality of the food supply | National action plan for reducing salt intake in the diet of the population of Slovenia for the period 2010-2020 | 2010 | Policy | The policy promotes collaboration to reduce salt in key foods, educate the public, and set targets (2010–2020) to reduce cardiovascular and salt-related health issues in Slovenia. | To reduce salt intake and related health issues through collaboration and public education. | All food products | Salt/sodium |  | [World Action on Salt: Slovenia National Action Plan on Reduced Salt Intake](https://www.worldactiononsalt.com/worldaction/europe/slovenia/) | Policy documentation / salt-reduction programme page |
| **Sl_4** | Offer healthy food and set standards in public institutions and other specific settings | School Nutrition Act | 2013 | Law | The policy emphasizes compliance with nutritional guidelines and prohibits vending machines for food and drinks in school areas, allowing them only in staff rooms for hot beverages. | To ensure high-quality school meals that promote student development. | School food products | Nutrients | X | [FAOLEX record: School Nutrition Act](https://www.fao.org/faolex/results/details/en/c/LEX-FAOC208544) | FAOLEX specific legal record |
| **Sl_5** | Set incentives and rules to create a healthy retail and food service environment | National Programme for Nutrition and Physical Activity 2015-2025 | 2015 | Policy | The policy aims to improve nutrition and physical activity, reduce obesity and chronic diseases, and address health disparities by promoting healthy eating, physical activity, and access to nutritious food. | To enhance health and quality of life in Slovenia by promoting better dietary and physical activity habits. | All food products | Nutrients |  | [Dober tek Slovenija PDF: National Programme for Nutrition and Physical Activity 2015-2025](https://www.dobertekslovenija.si/wp-content/uploads/2018/06/brosura_DTS_angl_A4_za_print.pdf) | Official national programme PDF / implementation brochure |
| **Sl_6** | Improve the nutritional quality of the food supply | Slovenia's Ban on Industrially Produced Trans Fatty Acids (iTFAs) | 2018 | Law | This law imposed voluntary or mandatory measures to minimize the content of industrial TFAs (iTFAs) in the food supply. | To eliminate iTFAs from the food supply. | All food products | Trans Fatty Acids |  | [PISRS record: Regulation on maximum permitted trans-fatty-acid content in foods](https://pisrs.si/pregledPredpisa?id=PRAV13448) | Official Slovenian legal information system record |
| **Sl_7** | Harness supply chain and actions across sectors to ensure coherence with health | Order on Programme for the promotion of the fruit sector for the period 2021-2023. | 2021 | Other (regulation) | The regulation establishes Slovenia's Fruit Sector Promotion Program for 2021–2023, mandating promotional activities, "selected quality" participation for 15% of orchards, and financial contributions for fruit sector products. | To promote Slovenia's fruit sector through key activities and financial contributions. | All food products | Not specified |  | [FAOLEX record: Programme for promotion of fruit sector 2021-2023](https://www.fao.org/faolex/results/details/en/c/LEX-FAOC206345) | FAOLEX specific legal record |
| **Sl_8** | Offer healthy food and set standards in public institutions and other specific settings | Order on the day of the implementation of the project Traditional Slovenian Breakfast. | 2021 | Other (ministerial order) | The Order sets the implementation date for the Traditional Slovenian Breakfast project, promoting local foods and healthy eating habits in schools and institutions for children with special needs. | To promote Slovenian cuisine and healthy eating habits in schools and institutions. | School food products | Not specified | X | [FAOLEX record: Traditional Slovenian Breakfast Day order](https://www.fao.org/faolex/results/details/en/c/LEX-FAOC206505) | FAOLEX specific legal record |
| **ES_1** | Restrict food advertising and other forms of commercial promotion | Law 34/1988 - Ley General de Publicidad | 1988 | Law | Applicable law in Spain about advertising. It establishes the requirement about legal and illegal advertising (misleading, unfair, subliminal) in product labeling. | To regulate advertising in general, including food | All food products | Not specified |  | [BOE record: Ley 34/1988, General Advertising Law](https://www.boe.es/buscar/act.php?id=BOE-A-1988-26156) | Official gazette legal text |
| **ES_2** | Nutrition label standards and regulations on the use of claims and implied claims on food | Royal Decree 930/1992 of 17 July 1992 on the labeling of foodstuffs for human consumption.National transposition  of Directive 90/ 496. | 1992 | Other (decree) | The Royal Decree sets labeling requirements for food products in Spain to ensure clear and accurate information for consumers. | To standardize food labeling for consumer health and informed choices. | All food products | Not specified |  | [BOE record: Royal Decree 930/1992 on nutrition labelling](https://www.boe.es/buscar/act.php?id=BOE-A-1992-18639) | Official gazette legal text |
| **ES_3** | Inform people about food and nutrition through public awareness | Spanish strategy for nutrition, physical activity and prevention of obesity (NAOS) | 2005 | Policy | The strategy aims to improve dietary habits and physical activity through comprehensive policies, public awareness campaigns, and school education. | To combat obesity by promoting healthy eating and physical activity. | School food products | Not specified | X | [AESAN PDF: NAOS Strategy](https://www.aesan.gob.es/AECOSAN/docs/documentos/nutricion/NAOS_Strategy.pdf) | Official government strategy PDF |
| **ES_4** | Restrict food advertising and other forms of commercial promotion | Codigo (PAOS) | 2005 | Policy | The PAOS Code regulates food and beverage marketing to children under 12, ensuring advertisements are responsible, accurate, and do not exploit children's inexperience. | To ensure responsible marketing of food and beverages to children under 12. | All food products | Not specified | X | [AESAN PDF: PAOS Code 2005](https://www.aesan.gob.es/AECOSAN/docs/documentos/nutricion/Codigo_PAOS_2005_ingles.pdf) | Official government-hosted code PDF |
| **ES_5** | Nutrition label standards and regulations on the use of claims and implied claims on food | Real Decreto 867/2008 RTS preparados para lactantes y de continuación | 2008 | Other (regulation) | This regulation sets standards for the composition, labeling, and advertising of infant and follow-on formulas. | To ensure the safety and proper nutrition of infants. | Infant food products | Not specified | X | [BOE record: Royal Decree 867/2008 on infant formula and follow-on formula](https://www.boe.es/buscar/doc.php?id=BOE-A-2008-9289) | Official gazette legal text |
| **ES_6** | Harness supply chain and actions across sectors to ensure coherence with health | Spanish Law on Food Safety and Nutrition (Law 17/2011 of July 5) | 2011 | Law | The law specifies requirements for food labeling, ensuring accurate information on composition, nutritional values, allergens, and other essential details for consumer safety and informed choices. | To regulate and enforce standards that guarantee the safety and nutritional adequacy of food products. | All food products | Nutrients |  | [BOE record: Law 17/2011 on food safety and nutrition](https://www.boe.es/buscar/act.php?id=BOE-A-2011-11604) | Official gazette legal text |
| **ES_7** | Nutrition label standards and regulations on the use of claims and implied claims on food | Royal Decree 126/2015 of 27 February 2015 on consumer information for the marketing of extended warranties and guarantees for consumer goods. | 2015 | Other (decree) | The Royal Decree approves rules on food information for unpackaged foods and those packaged at the point of sale or by retail owners at the buyer's request. | To standardize information on extended warranties and guarantees, promoting fair consumer practices. | All food products | Not specified |  | [BOE record: Royal Decree 126/2015 on food information for unpackaged foods](https://www.boe.es/buscar/act.php?id=BOE-A-2015-2293) | Official gazette legal text |
| **ES_8** | Harness supply chain and actions across sectors to ensure coherence with health | Collaboration plan for the improvement of the composition of food and beverages and other measures 2020 | 2017 | Policy | The policy aims to reduce added sugars by 10% and continue lowering salt, saturated, and trans fats in commonly consumed foods by 2020, ensuring no increase in caloric content. | To reduce added sugars, salt, saturated, and trans fats by 10% without increasing calories. | All food products | Salt/sodium |  | [AESAN PDF: Collaboration Plan for improving food and beverage composition 2020](https://www.aesan.gob.es/AECOSAN/docs/documentos/nutricion/PLAN_COLABORACION_2020.pdf) | Official government policy PDF |
| **ES_9** | Harness supply chain and actions across sectors to ensure coherence with health | Real Decreto 308/2019, de 26 de abril, por el que se aprueba la norma de calidad para el pan | 2019 | Other (decree) | The decree sets new quality standards for bread production and commercialization in Spain, updating previous regulations to reflect technological advancements and changing consumer habits. | To establish updated quality standards for bread production. | Bread/bakery products | Not specified |  | [BOE record: Royal Decree 308/2019 approving the quality standard for bread](https://www.boe.es/buscar/doc.php?id=BOE-A-2019-6994) | Official gazette legal text |
| **ES_10** | Harness supply chain and actions across sectors to ensure coherence with health | Real Decreto 1086/2020, de 9 de diciembre, por el que se regulan y flexibilizan determinadas condiciones de aplicación de las disposiciones de la Unión Europea en materia de higiene de la producción y comercialización de los productos alimenticios y se regulan actividades excluidas de su ámbito de aplicación. | 2020 | Other (decree) | The Real Decreto regulates EU hygiene provisions for food production and marketing, while also defining excluded activities. | To align Spain's food safety laws with European standards, while respecting traditional methods and regional needs. | All food products | Not specified |  | [BOE record: Royal Decree 1086/2020 on hygiene for production and marketing of food products](https://www.boe.es/buscar/doc.php?id=BOE-A-2020-15872) | Official gazette legal text |
| **ES_11** | Nutrition label standards and regulations on the use of claims and implied claims on food | Nutri-Score Labeling Policy | 2021 | Policy | This policy uses a color-coded system from dark green (A) to dark orange (E) to indicate food nutritional quality, based on positive (e.g., fruits) and negative (e.g., saturated fats) components per 100g/ml. | To enhance product comparability and ensure effective implementation. | All food products | Fat |  | [La Moncloa: Nutri-Score food-labelling announcement](https://www.lamoncloa.gob.es/lang/en/gobierno/news/paginas/2020/20200626food-labelling.aspx) | Official government policy announcement |
| **ES_12** | Improve the nutritional quality of the food supply | Royal Decree 308/2019, of April 26, approving the quality standard for bread | 2022 | Other (decree) | The royal decree defines bread as a product of flour and water, optionally with salt, fermented with yeast or sourdough. It sets allowable ingredients and caps salt. | To sets out basic quality standards for the production and sale of bread. | Bread/bakery products | Salt/sodium |  | [BOE record: Royal Decree 308/2019 approving the quality standard for bread](https://www.boe.es/buscar/doc.php?id=BOE-A-2019-6994) | Official gazette legal text |
| **ES_13** | Improve the nutritional quality of the food supply | Royal Decree 308/2019, of April 26, approving the quality standard for bread | 2022 | Other (decree) | This decree defines bread as a product made from flour and water, with or without salt, and fermented with yeast or sourdough. It limits salt content to a maximum of 1.31 grams per 100 grams. | To set out basic quality standards for the production and sale of bread in Spain | Bread/bakery products | Salt/sodium |  | [Ministry of Agriculture page: entry into force of bread salt limit](https://www.mapa.gob.es/es/prensa/ultimas-noticias/detalle_noticias/entra-en-vigor-la-norma-que-limita-el--contenido-m-ximo-de-sal-en-el-pan/f1a39820-dd7f-4967-9ac6-e24c88b64115) | Official government policy/legal implementation page |
| **SE_1** | Nutrition label standards and regulations on the use of claims and implied claims on food | Keyhole labeling policy | 1985 | Other (regulation) | The policy promotes Keyhole-labeled grain products with 0% VAT on Keyhole-labeled bread and cereals, 34.2% VAT on bakery goods and ready meals, and a 50% subsidy for Keyhole-labeled items. | To make it easier for consumers to find and choose healthier products. | Bread/bakery products | Not specified |  | [Swedish Food Agency: Keyhole label](https://www.livsmedelsverket.se/en/food-habits-health-and-environment/nyckelhalet/) | Official government food-agency policy page |
| **SE_2** | Harness supply chain and actions across sectors to ensure coherence with health | Swedish Plan of Action for Nutrition | 1995 | Policy | This policy aims to increase dietary fiber intake by 25-30 grams per person through fiber-rich foods and reducing fat intake to 30% of total energy needs. | To improve public health by promoting healthy diets, exercise. | All food products | Fat |  | [Riksdag/SOU documentation: Swedish national action plan for nutrition](https://www.riksdagen.se/sv/dokument-och-lagar/dokument/statens-offentliga-utredningar/halsa-pa-lika-villkor-del-4_gob391d4/html/) | Official parliamentary/SOU documentation |
| **SE_3** | Nutrition label standards and regulations on the use of claims and implied claims on food | Regulation (2005:9) on the use of a particular symbol (Keyhole) | 2005 | Other (regulation) | The Regulation amends the National Food Agency’s (Sweden) regulations (SLVFS 2005:9) regarding the use of a specific symbol. | To ensure correct use of the symbol and improve transparency for consumers. | All food products | Not specified |  | [FAOLEX PDF: Swedish Keyhole symbol regulation LIVSFS 2005:9](https://faolex.fao.org/docs/pdf/swe199505.pdf) | FAOLEX hosted legal text PDF |
| **SE_4** | Harness supply chain and actions across sectors to ensure coherence with health | Regulation on official control of foodstuffs (LIVSFS 2005:21). | 2005 | Other (regulation) | The Regulation covers facility approval, product control, meat and milk inspection, sampling, analysis, and laboratories. | To contains rules on official control of food. | Meat products. | Not specified |  | [Swedish Food Agency PDF: Regulation LIVSFS 2005:21 on official food control](https://www.livsmedelsverket.se/4aacc3/globalassets/om-oss/lagstiftning/offentlig-kontroll/livsfs-2005-21.pdf) | Official Swedish Food Agency regulation PDF |
| **SE_5** | Use economic tools to address food affordability and purchase incentives | Regulation (2006:1166) on Fees for Official Control of Food and Certain Agricultural Products | 2006 | Other (decree) | This Decree sets rules for fees payable to authorities for the official control of food and agricultural products, covering costs for public control of food and organic production. | To establish fees for official control of food and agricultural products. | All food products | Not specified |  | [Riksdag record: Regulation 2006:1166 on fees for official food control](https://www.riksdagen.se/sv/dokument-och-lagar/dokument/svensk-forfattningssamling/forordning-20061166-om-avgifter-for-offentlig_sfs-2006-1166/) | Official Swedish legal text database record |
| **SE_6** | Harness supply chain and actions across sectors to ensure coherence with health | Food Act (2006:804). | 2006 | Law | The Act applies to all stages of food production, processing, and distribution, granting the Government significant regulatory powers over food hygiene, safety, and labeling. | To assure a high level of protection of human health and consumers’ interests in relation to food. | All food products | Not specified |  | [Riksdag record: Food Act 2006:804](https://www.riksdagen.se/sv/dokument-och-lagar/dokument/svensk-forfattningssamling/livsmedelslag-2006804_sfs-2006-804/) | Official Swedish legal text database record |
| **SE_7** | Harness supply chain and actions across sectors to ensure coherence with health | Nordic plan of action on better health and quality of life through diet and physical activity | 2006 | Policy | The policy includes a biennial catalogue of Nordic health initiatives, improved nutrition and physical activity in schools and daycare centers, and collaboration with the education sector to promote healthy habits among children and youth. | To improve dietary habits and physical activity. | School food products | Nutrients | X | [Nordic Council of Ministers publication: A better life through diet and physical activity](https://www.norden.org/en/publication/better-life-through-diet-and-physical-activity) | Nordic Council of Ministers policy publication |
| **SE_8** | Nutrition label standards and regulations on the use of claims and implied claims on food | Regulation on infant formulas and supplementary nutrition (LIVSFS 2008:2). | 2008 | Other (regulation) | This Regulation covers the composition and labeling of breast-milk substitutes and supplementary foods for healthy infants. | To set rules for the composition and labeling of infant formulas and supplementary nutrition. | dietary supplements | Not specified | X | [Swedish Food Agency PDF: Regulation LIVSFS 2008:2 on infant formula and follow-on formula](https://www.livsmedelsverket.se/4927b7/globalassets/om-oss/lagstiftning/nummerordning---upphord-lagstiftning/2008/livsfs-2008-2.pdf) | Official Swedish Food Agency regulation PDF |
| **SE_9** | Restrict food advertising and other forms of commercial promotion | Swedish Marketing Act | 2008 | Law | This legislation prohibits the advertising of unhealthy foods to children through various media platforms to promote healthier dietary choices. | To reduce children's exposure to advertisements for unhealthy foods. | All food products | Not specified | X | [Riksdag record: Swedish Marketing Act 2008:486](https://www.riksdagen.se/sv/dokument-och-lagar/dokument/svensk-forfattningssamling/marknadsforingslag-2008486_sfs-2008-486/) | Official Swedish legal text database record |
| **SE_10** | Offer healthy food and set standards in public institutions and other specific settings | Education Act 2010:800 | 2010 | Law | The Education Act requires that school meals in compulsory schools be free of charge, nutritious, and based on Swedish nutritional recommendations. | To ensure that all students in Sweden have access to free, nutritious meals while at school. | School food products | Nutrients | X | [Riksdag record: Education Act 2010:800](https://www.riksdagen.se/sv/dokument-och-lagar/dokument/svensk-forfattningssamling/skollag-2010800_sfs-2010-800/) | Official Swedish legal text database record |
| **SE_11** | Nutrition label standards and regulations on the use of claims and implied claims on food | Regulation (LIVSFS 2014: 4) on food information. | 2014 | Other (regulation) | This Regulation sets guidelines for food information, covering all foods, including catering, with rules on language, packaging, quantity, non-prepackaged foods, veal, potatoes, and notifications. | To regulate food information. | All food products | Not specified |  | [Swedish Food Agency PDF: Regulation LIVSFS 2014:4 on food information](https://www.livsmedelsverket.se/48d6a4/globalassets/om-oss/lagstiftning/livsmedelsinfo-till-konsum---markning/livsfs-2014-4.pdf) | Official Swedish Food Agency regulation PDF |
| **SE_12** | Harness supply chain and actions across sectors to ensure coherence with health | Action plan for overweight and obesity (2016-2020) | 2016 | Policy | The policy aims to reduce adult overweight to below 25% and obesity to below 7%, lower overweight in 4-year-olds to under 7% and obesity to under 2%, and decrease overweight in first-time pregnant women to under 15% and obesity to under 5%. | To improve public health outcomes related to weight and dietary habits. | All food products | Not specified |  | [Stockholm public health PDF: Action plan for overweight and obesity 2016-2020](https://dok.slso.sll.se/CES/FHG/Folkhalsoarbete/Informationsmaterial/Handlingsprogram-overvikt-fetma-2016-2020.pdf) | Official public health action-plan PDF |
| **SE_13** | Harness supply chain and actions across sectors to ensure coherence with health | Regulation on the fortification of certain foods (LIVSFS 2018:5). | 2018 | Other (regulation) | The Regulation mandates the enrichment of certain foods intended for final consumers or large families. | To mandate the enrichment of certain foods for final consumers or large families. | All food products | Not specified |  | [Swedish Food Agency PDF: Regulation LIVSFS 2018:5 on fortification of certain foods](https://www.livsmedelsverket.se/globalassets/om-oss/lagstiftning/berikn---kosttillsk---livsm-spec-gr-fsmp/livsfs-2018-5-kons-2024-9.pdf) | Official Swedish Food Agency regulation PDF |
| **SE_14** | Harness supply chain and actions across sectors to ensure coherence with health | Consumer Purchase Act (2022:260) | 2022 | Law | The Act governs food purchases from traders to consumers, ensuring that goods meet quality and safety standards and providing a framework for addressing delays and defects. | To protect consumers in purchases. | All food products | Not specified |  | [Riksdag record: Consumer Purchase Act 2022:260](https://www.riksdagen.se/sv/dokument-och-lagar/dokument/svensk-forfattningssamling/konsumentkoplag-2022260_sfs-2022-260/) | Official Swedish legal text database record |
| **UA_1** | Harness supply chain and actions across sectors to ensure coherence with health | Order No. 2646 of the Ministry of Public Health validating the Regulation on Food Safety Indicators "Maximum limits (levels) of residues of active substances of veterinary drugs in foodstuffs of animal origin". | 2019 | Other (ministerial order) | The order approves the food safety indicators for the maximum permissible levels of veterinary drug residues in animal-origin food products. | To establish maximum limits for veterinary drug residues in animal-origin food products. | Animal-origin products | Pesticides/residues |  | [Verkhovna Rada record: Ministry of Health Order No. 2646 on veterinary drug residues](https://zakon.rada.gov.ua/go/z0042-20) | Official Ukrainian legal database record |
| **UA_2** | Harness supply chain and actions across sectors to ensure coherence with health | Order No. 610-21 on permissible levels of dioxins, dioxin-like polychlorinated biphenyls and non-dioxin in foodstuffs. | 2021 | Other (regulation) | Regulation on sampling methods to determine maximum dioxin and PCB levels in food for state control. | To promote food safety. | All food products | Not specified |  | [Verkhovna Rada record: Order No. 610-21 on dioxins and PCBs in foodstuffs](https://zakon.rada.gov.ua/go/z1480-21) | Official Ukrainian legal database record |
| **UA_3** | Harness supply chain and actions across sectors to ensure coherence with health | Decree No. 102 on the procedure for determining the periodicity of planned measures in organic production, circulation and labelling of organic products. | 2022 | Other (decree) | The Decree regulates organic food production, circulation, and labeling, establishing state control procedures, inspection frequencies, and monitoring to ensure compliance with organic standards. | To ensure the quality and integrity of organic food in Ukraine, protecting consumers. | All food products | Not specified |  | [Verkhovna Rada record: Cabinet Decree No. 102 on organic production controls](https://zakon.rada.gov.ua/go/102-2022-%D0%BF) | Official Ukrainian legal database record |
| **UA_4** | Harness supply chain and actions across sectors to ensure coherence with health | Order No. 209 of the Ministry of Agrarian Policy and Food validating Hygienic requirements for small-scale production and circulation of milk. | 2022 | Other (ministerial order) | These requirements set hygienic standards for milk production and circulation from small-scale farm producers. | To promote food safety. | Dairy products | Not specified |  | [Verkhovna Rada record: Ministry of Agrarian Policy Order No. 209 on small-scale milk hygiene](https://zakon.rada.gov.ua/go/z0452-22) | Official Ukrainian legal database record |
| **UA_5** | Set incentives and rules to create a healthy retail and food service environment | Order No. 2199 of the Ministry of Health validating the Requirements for materials and objects containing vinyl chloride and intended for contact with foodstuffs. | 2022 | Other (ministerial order) | The Order sets safety indicators for materials containing vinyl chloride intended for food contact. | To establish safety standards for vinyl chloride-containing materials in food contact. | All food products | Not specified |  | [Verkhovna Rada record: Ministry of Health Order No. 2199 on vinyl chloride food-contact materials](https://zakon.rada.gov.ua/go/z1641-22) | Official Ukrainian legal database record |
| **UA_6** | Harness supply chain and actions across sectors to ensure coherence with health | Law No. 2572-IX on indications for agricultural commodities and foodstuffs. | 2022 | Law | This law defines the legal and organizational principles of quality schemes for agricultural commodities and foodstuffs, | To establish legal and organizational principles for quality schemes in agricultural commodities and foodstuffs. | All food products | Not specified |  | [Verkhovna Rada record: Law No. 2572-IX on geographical indications for agricultural products and foodstuffs](https://zakon.rada.gov.ua/go/2572-20) | Official Ukrainian legal database record |
| **UA_7** | Harness supply chain and actions across sectors to ensure coherence with health | Law No. 2573-IX "On the system of public health". | 2022 | Law | This law applies to foodstuffs involved in epidemiological investigations of infectious and non-infectious diseases. | To ensure food safety by regulating foodstuffs. | All food products | Not specified |  | [Verkhovna Rada record: Law No. 2573-IX on the public health system](https://zakon.rada.gov.ua/go/2573-20) | Official Ukrainian legal database record |
| **UA_8** | Set incentives and rules to create a healthy retail and food service environment | Rules for adding vitamins, minerals and some other substances to food products | 2023 | Policy | This policy allows only specific vitamins and minerals to be added to food products and limits trans fats in consumer goods. | To regulate vitamins and minerals in food and control harmful substances, including trans fats, for consumer safety. | All food products | Trans Fatty Acids |  | [Verkhovna Rada record: Rules for adding vitamins, minerals and other substances to food products](https://zakon.rada.gov.ua/go/z0891-20) | Official Ukrainian legal database record |

**Note.** Each national policy action has been assigned to a unique identification code following the format ISO Code_n. The ISO Alpha-2 country code corresponds to the country of reference (e.g., AT for Austria), while the numeric suffix indicates the temporal order national policy action, starting from the earliest (e.g., AT_1 represents the oldest policy retrieved for Austria, while AT_4 refers to its most recent one. The countries included in the dataset, along with their respective ISO Alpha-2 codes, are provided as follows: Austria (AT), Belgium (BE), Bulgaria (BG), Croatia (HR), Cyprus (CY), Czech Republic (CZ), Denmark (DK), Estonia (EE), Finland (FI), France (FR), Germany (DE), Greece (GR), Hungary (HU), Ireland (IE), Italy (IT), Latvia (LV), Lithuania (LT), Luxembourg (LU), Malta (MT), The Netherlands (NL), Poland (PL), Portugal (PT), Romania (RO), Slovakia (SK), Slovenia (SI), Spain (ES), Sweden (SE), Montenegro (ME), Republic of Moldova (MD), North Macedonia (MK), and Ukraine (UA).

**Note.** Where multiple sources referred to the same national policy action, the action was listed once under its unique policy identifier, and supporting sources were used for verification rather than counted as separate policy entries.

**Supplementary Table S4.** Country distribution of included national food and nutrition policy actions (N = 379)

| **Country** | **Policy actions n (%)** |
| --- | --- |
| Republic of Moldova | 62 (16.4%) |
| Germany | 26 (6.9%) |
| Bulgaria | 21 (5.5%) |
| Croatia | 19 (5.0%) |
| Montenegro | 19 (5.0%) |
| France | 17 (4.5%) |
| Belgium | 14 (3.7%) |
| Spain | 14 (3.7%) |
| Sweden | 14 (3.7%) |
| Denmark | 13 (3.4%) |
| Finland | 13 (3.4%) |
| Hungary | 12 (3.2%) |
| Poland | 11 (2.9%) |
| Czech Republic | 10 (2.6%) |
| Netherlands | 9 (2.4%) |
| Portugal | 9 (2.4%) |
| Romania | 9 (2.4%) |
| Greece | 8 (2.1%) |
| Italy | 8 (2.1%) |
| Malta | 8 (2.1%) |
| Slovenia | 8 (2.1%) |
| Ukraine | 8 (2.1%) |
| Estonia | 7 (1.8%) |
| Ireland | 7 (1.8%) |
| Latvia | 7 (1.8%) |
| Lithuania | 6 (1.6%) |
| Luxembourg | 5 (1.3%) |
| North Macedonia | 5 (1.3%) |
| Austria | 4 (1.1%) |
| Slovakia | 4 (1.1%) |
| Cyprus | 3 (0.8%) |

**Note.** Country-level counts represent the number of unique national policy actions identified for each country. These counts reflect policy volume and should not be interpreted as direct measures of policy comprehensiveness, implementation strength, or breadth across NOURISHING domains. Domain breadth is assessed separately using the NOURISHING domain coverage score reported in Table 2.
